# Anion identification using silsesquioxane cages†
†Electronic supplementary information (ESI) available: Experiments, characterization and spectroscopic studies. See DOI: 10.1039/c8sc02959h


**DOI:** 10.1039/c8sc02959h

**Published:** 2018-09-25

**Authors:** Supphachok Chanmungkalakul, Vuthichai Ervithayasuporn, Patcharaporn Boonkitti, Alisa Phuekphong, Nicha Prigyai, Sumana Kladsomboon, Suda Kiatkamjornwong

**Affiliations:** a Department of Chemistry , Center of Excellence for Innovation in Chemistry (PERCH-CIC) , Center for Inorganic and Materials Chemistry , Faculty of Science , Mahidol University , Rama VI Road, Ratchathewi , Bangkok 10400 , Thailand . Email: vuthichai.erv@mahidol.ac.th ; Email: vuthichai.erv@mahidol.edu; b Department of Radiological Technology , Faculty of Medical Technology , Mahidol University , Nakhon Pathom 73170 , Thailand; c Faculty of Science , Chulalongkorn University , Phayathai Road , Bangkok 10330 , Thailand; d FRST , Division of Science , The Royal Society of Thailand , Sanam Suepa , Dusit , Bangkok 10300 , Thailand

## Abstract

Anthracene-conjugated octameric silsesquioxane (AnSQ) cages, prepared *via* Heck coupling between octavinylsilsesquioxane (OVS) and 9-bromoanthracene, thermodynamically display intramolecular excimer emissions. More importantly, these hosts are sensitive to each anionic guest, thereby resulting in change of anthracene excimer formation, displaying the solvent-dependent fluorescence and allowing us to distinguish up to four ions such as F^–^, OH^–^, CN^–^ and PO_4_^3–^ by fluorescence spectroscopy. Depending on the solvent polarity, for example, both F^–^ and CN^–^ quenched the fluorescence emission intensity in THF, but only F^–^ could enhance the fluorescence in all other solvents. The presence of PO_4_^3–^ results in fluorescence enhancements in high polarity solvents such as DMSO, DMF, and acetone, while OH^–^ induces enhancements only in low polarity solvents (*e.g.* DCM and toluene). A picture of the anion recognizing ability of AnSQ was obtained through principal component analysis (PCA) with NMR and FTIR confirming the presence of host–guest interactions. Computational modeling studies demonstrate the conformation of host–guest complexation and also the change of excimer formation. Detection of F^–^, CN^–^ and OH^–^ by AnSQ hosts in THF is noticeable with the naked eye, as indicated by strong color changes arising from charge transfer complex formation upon anion addition.

## Abstract 2

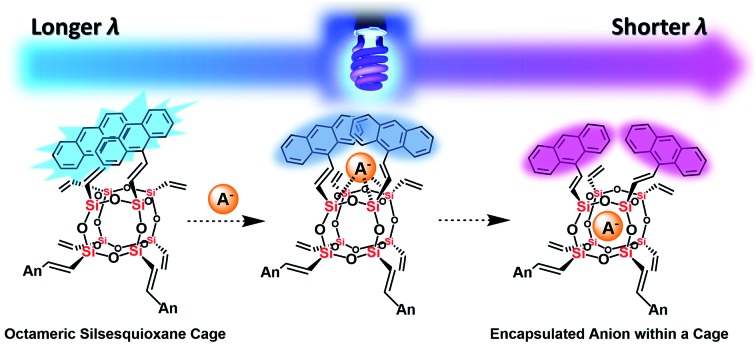
Anthracene-conjugated octameric silsesquioxane cages thermodynamically display intramolecular excimer formation, which can be used to identify anions through the change of fluorescence.

## Introduction

Silica is one of the most naturally abundant compounds on the Earth. It is used in building materials, electronic devices, in environmental remediation, and in chemical industry.^1^ Silica gel is a commonly used laboratory sorbent for chromatographic separations, the binding properties of which can be tailored through surface functionalization.^2^ For example, amine groups appended to the silica surface allow for chemical adsorption of CO_2_, which generates a chelating group allowing sequestration of metal ions.^3^,^4^ Interestingly, the mode of adsorption for organic molecules by silica is still controversial. While a mechanism has been postulated^5^ and the outstanding capacity and absorptivity properties of silica are well-known,^6^,7a very few studies have been devoted to determine the actual role of silica in the adsorption of organic species.7b,c This may relate to the poor solubility of these sorbents in water or organic solvents, hindering investigations using solution phase techniques (fluorescent emission, UV-vis absorption or nuclear magnetic resonance).^8^

Polyhedral oligomeric silsesquioxanes (POSS) or silsesquioxane (SQ) cages may be considered as representative molecules for silica due to their closely related empirical formulae (RSiO_1.5_).^9^ These systems consist of a rigid cage-like silica framework with organic groups attached to the periphery. The properties of these inorganic–organic hybrid molecules (*e.g.*, solubility) can be altered or tailored to specific applications through changing the organic functionalities.^10^ The higher solubility of SQ relative to silica allows for more facile characterization and study of these systems at the molecular level.^11^ Accordingly, technological applications of SQ as biocompatible materials,^12^ organic light emitting diodes (OLEDs),^13^ hybrid polymer^14^ porous materials for CO_2_ capture,^15^ in nitroaromatics detection,^16^ and catalysts^17^ have arisen over the last decade.

Polyhedral SQ cages can be considered as host molecules, whereby guest species (atoms, ions or molecules) can interact through encapsulation by the internal cavity, or through facial interactions. Solid state encapsulation of fluoride ion within a SQ cage^18^ led to further computational studies, which suggested that encapsulation is enhanced if the cage is electron deficient.^19^ SQs appended with conjugated pendant organic substituents (*e.g.*, vinyl, phenyl) containing electron withdrawing –CF_3_ groups exhibited diffusive negative contours at the exterior and more condensed positive contours within the cage interior. In comparison, cages appended with aliphatic electron donating substituents exhibited condensed negative contours within the interior, rendering fluoride ingress less favorable.^19^ These data have been exploited by Aziz *et al.* for the preparation of many silsesquioxane-based receptor systems,^20^ in addition to fluorescent sensors based on pyrene-conjugated silsesquioxane cages for fluoride detection.^21^

Anion recognition studies form an integral part of modern sensor design and research. While host–guest recognition processes mostly rely on weak non-covalent interactions (*e.g.*, hydrogen bonding, π–π interactions), protonation, deprotonation and nucleophilic substitution are also important modes underlying sensor function.^22^,^23^ This work highlights design of a SQ cage coupled with fluorophores as a host for anion sensing. Recognition by the approach of anions through the face of the octahedral (*O*_h_) cage causes changes in the fluorophore environment, as observed by fluorescence perturbations. Anthracene was chosen as the fluorophore based on past work by James *et al.*, who integrated it into his glucose sensor platform for monitoring of blood-sugar levels in diabetic patients.^24^ Commonly used in photochemistry,^25^ anthracene and its derivatives exhibit numerous fluorescence modes (*e.g.* types of excimers: twisted, side to face in T-shape and end-overlapped).^26^ As the fluorescence response of anthracene-containing molecules depends on the spacing of these motifs and the nature of the linkages, not on their concentration,27*a* appending multiple anthracene groups to the periphery of the octahedral SQ of the T_8_ cage allows for the possibility of significant geometry distortion (and hence sensing), on exposure to complementary anions.27*b*

In this work, the synthesis of an anthracene-conjugated octameric silsesquioxane (AnSQ) is presented. The anion recognition ability of this host was probed through monitoring of fluorescence emission and UV-vis absorption changes, on exposure. Complementary *in silico* calculations for T_8_ cages provide insights into the flexibility of anthracenes with variations in the spacer, along with electrostatic potential mapping of the AnSQ cage interior to determine the potential for, and likely mode of, anion ingress.

## Results and discussion

### Synthesis

**Scheme 1:**
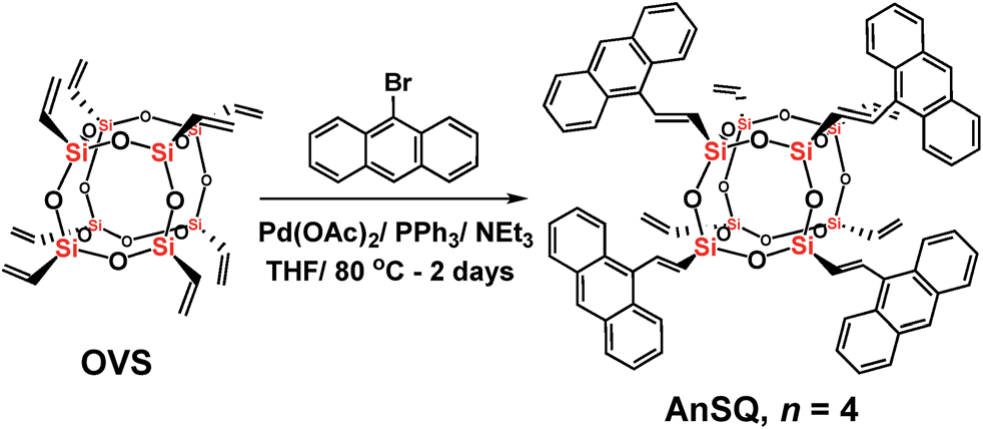
Synthesis of anthracene-conjugated octameric silsesquioxane (AnSQ) cages.

The AnSQ cage (Scheme 1) was prepared in 86% yield through a Heck-coupling reaction between octavinylsilsesquioxane (OVS) and 9-bromoanthracene. The MALDI-TOF spectrum (Fig. S1†) with *trans*-2-3-(4-*tert*-butylphenyl)-2-methyl-2-propenylidenemalononitrile (DCTB) as a matrix showed the distribution of products, with that corresponding to *n* = 4 being the most abundant. Increasing the ratio of 9-bromoanthracene to OVS under these synthetic conditions did not significantly alter the product distribution. Calcd for 4 substitutions of anthracene units in a silsesquioxane T8 cage (*n* = 4), calcd for C_72_H_56_O_12_Si_8_ + H^+^: *m*/*z* 1338.203 [M + H^+^]. Found: *m*/*z* 1338.110.

### Solvent and anion effects on excimer formation

**Fig. 1:**
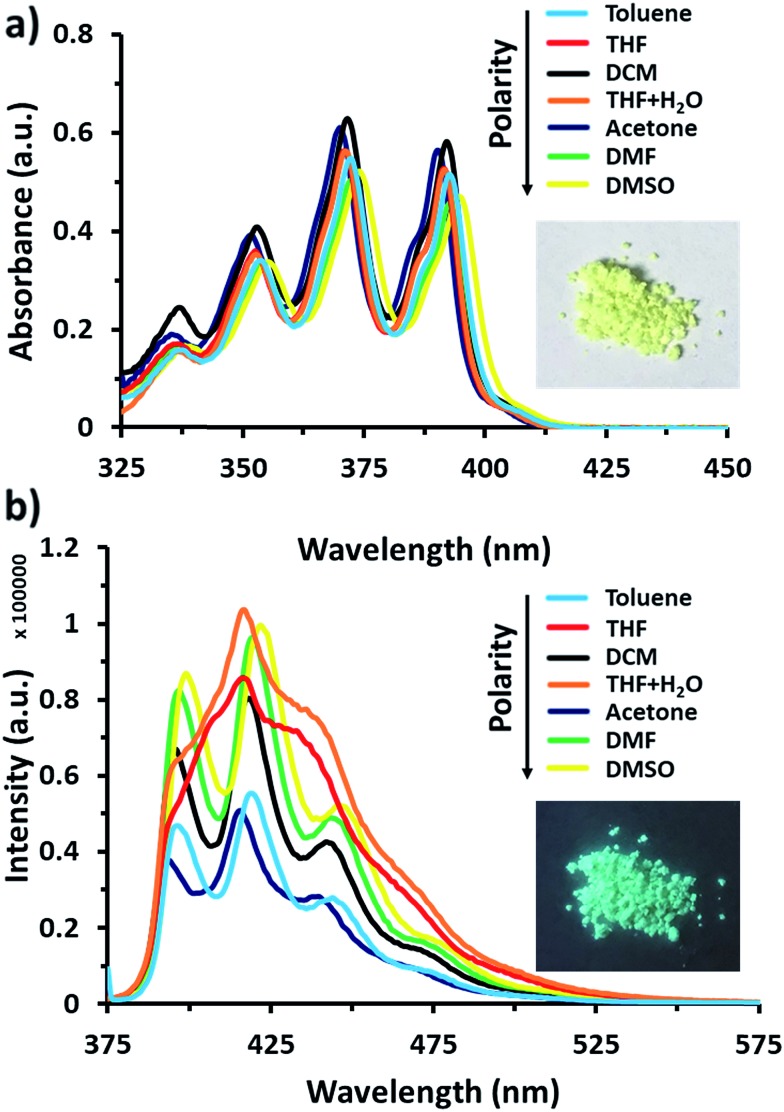
(a) UV-vis absorption of 9-bromoanthracene at 60 μM and a picture under visible light, (b) fluorescence emission of 9-bromoanthracene at 60 μM with *λ*_ex_ = 370 nm and a picture under UV light.

**Fig. 2:**
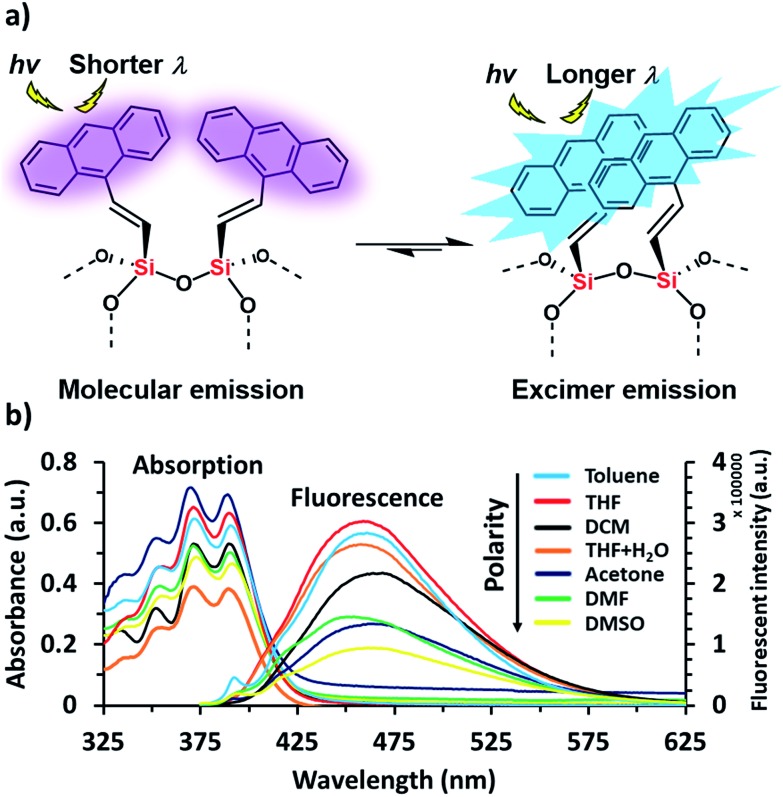
(a) Demonstration of thermodynamically stable of the excimer on AnSQ and (b) UV-vis absorption and fluorescence emission at *λ*_ex_ = 370 nm of AnSQ in various solvents at a concentration of 6 μM.

Anthracene derivatives possess solvent-dependent properties; change of solvent will slightly affect the absorption *λ*_max_ (Fig. 1a), the intensity of both absorption and emission.34c,d For example, 9-bromoanthracene used as a fluorescent labeling of AnSQ, its emissive intensity mostly increases with increase of solvent polarity as shown in Fig. 1b. Interestingly, 9-bromoanthracene in THF as a solvent showed very broad fluorescence emission. This phenomenon suggests that THF can also induce an intermolecular interaction among 9-bromoanthracene molecules leading to an aggregation with multilayer π–π stacks, even at low concentrations. Moreover, a solid state of 9-bromoanthracene shines a bright blue fluorescence (Fig. 1a) under UV radiation, confirming the presence of intermolecular excimer. However, after SQ cages coupled with anthracene functions, the fluorescence emission of AnSQ shown in Fig. 2b is significantly shifted to longer wavelengths with a larger Stokes shift (Δ*λ* = 85 nm), compared to 9-bromoanthracene (with a Stokes shift of Δ*λ* = 55 nm), as well as AnSQ in solid state emits a bright green fluorescence under UV light (Fig. 6b). This result confirms that strongly intramolecular interactions among adjacent anthracene groups (Fig. 2a) on AnSQ are thermodynamically favorable and simply found in all solvents as shown in Fig. 2b.28*a*

**Fig. 3:**
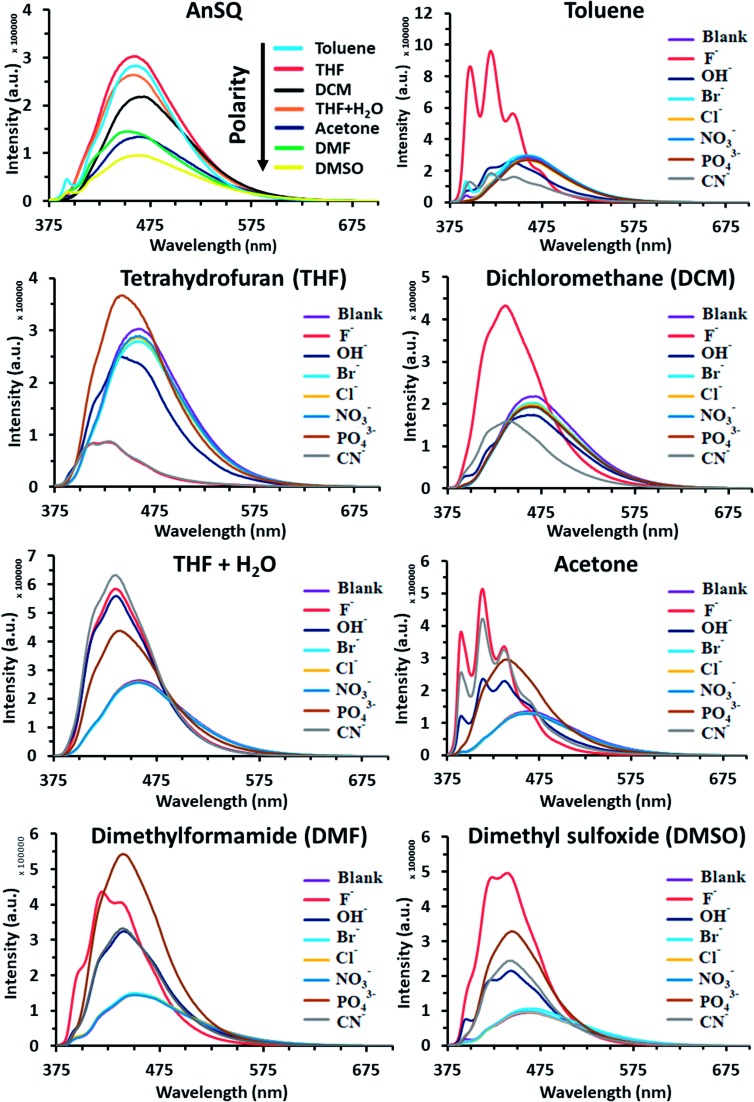
Fluorescence emission spectra (*λ*_ex_ = 370 nm) of AnSQ in various solvents before and after addition of guest anions (100 equiv.). Reaction time was set to 18 hours to ensure equilibrium was reached.

Such fluorescence emissions of AnSQ cage can be of two types: molecular and excimer emissions (Fig. 2a). The molecular emission, or π–π* fluorescence, of a fluorophore will show multiple peaks,28*b* whilst an excimer emission from an excited dimer shows a broad peak at a longer wavelength. In contrast to the solubility nature of 9-bromoanthracene, AnSQ is highly soluble in non-polar solvents, because the fluorescence intensity increased in low polar solvents (Fig. 2b). However, AnSQ tends to aggregate in high polar solvents like DMF or DMSO showing poor fluorescence intensity, as well as the intramolecular excimeric formation of adjacent anthracene functions on AnSQ can be instead interrupted by intermolecular π–π stacks of anthracene units with other AnSQ cages. To illustrate the effect of anion addition, fluorescence emission measurements of pristine AnSQ were first conducted in several solvents (Fig. 3). The emission intensities obtained could then be compared to those resulting from AnSQ in each solvent, after anion addition. As shown in Table 1, emission intensity and quantum yield generally decrease as the solvent polarity increases. Significant changes in *λ*_max_ of the AnSQ emission did not, however, correlate with solvent polarity. The trends in fluorescence intensity and quantum yield exhibited for AnSQ are reasonably consistent with those from previous work.^30^ The quantum yield measurements are provided in Fig. S3.†

**Table 1:** Fluorescence wavelength maxima (*λ*_max_) and quantum yields (*Φ*_P_) of AnSQ in various solvents at *λ*_ex_ = 370 nm before and after addition of guest anions (100 equiv.). Reaction time was set to 18 hours to ensure equilibrium was reached

Solvents	Fluorescence wavelength maxima (*λ*_max_; nm) and quantum yields (*Φ*_P_) on anion addition							
	Control	F^–^	OH^–^	Br^–^	Cl^–^	NO_3_^–^	PO_4_^3–^	CN^–^
DMSO	464, 0.158	440, 0.344	443, 0.131	460, 0.152	464, 0.136	460, 0.166	447, 0.242	444, 0.133
DMF	454, 0.283	419, 0.848	440, 0.288	449, 0.292	451, 0.314	452, 0.219	439, 0.646	440, 0.353
Acetone	464, 0.187	413, 0.460	414, 0.193	460, 0.324	459, 0.196	462, 0.224	442, 0.469	413, 0.324
THF + H_2_O	458, 0.428	435, 0.581	435, 0.405	457, 0.316	458, 0.381	457, 0.399	440, 0.528	436, 0.417
DCM	469, 0.260	436, 0.591	469, 0.396	466, 0.267	466, 0.263	465, 0.269	466, 0.277	436, 0.201
THF	457, 0.297	430, 0.004	441, 0.210	459, 0.323	458, 0.332	460, 0.322	442, 0.302	429, 0.009
Toluene	461, 0.394	419, 0.975	441, 0.475	459, 0.423	459, 0.391	460, 0.450	461, 0.351	420, 0.398

The fluorescence emission of AnSQ in the presence of 100 equiv. of anions in 6 μM of AnSQ, and all anion salts dissolved in THF except for PO_4_^3–^ which was dissolved in DI, was measured once equilibrium was reached (18 hours post-addition). Therefore, the results are highlighted as *λ*_max_ values in Table 1, in addition to quantum yields. In contrast to a previous study focusing on fluoride sensing,^21^ significant changes in AnSQ emission occur in the presence of other anions in addition to fluoride (OH^–^, PO_4_^3–^ and CN^–^). Addition of these to AnSQ in various solvents results in noticeable fluorescence emission perturbations (Fig. 3 and Table 1), manifested as the enhancement, quenching or Stokes shift of the fluorescence maxima. As an example, AnSQ in a moderately polar solvent (THF) exhibits a fluorescence wavelength maximum (*λ*_max_) at 457 nm with a quantum yield (*Φ*_P_) of 0.297. Addition of F^–^ results in lowering a Stokes shift of Δ*λ* = 60 nm with significant quenching to OFF (*Φ*_P_ = 0.004), confirming the formation of fluoride encapsulation by a SQ cage.^21^ We hypothesize that intramolecular excimer fluorescence of AnSQ was strongly disturbed by fluoride ion leading to reorganize the SQ cage framework and finally distort an excimer. This result would turn off the radiative transitions by the formation of “ion-induced aggregates” or “aggregation-caused quenching” (ACQ). Thereby, AnSQ became non-emissive in the aggregates even under highly diluted THF solution. Exposure of AnSQ to OH^–^, PO_4_^3–^ and CN^–^ results in similar *λ*_max_ shifts (to 441, 442 and 429 nm, respectively) and changes in quantum yield (*Φ*_P_ = 0.210, 0.302 and 0.009, respectively). While F^–^, OH^–^ and CN^–^ addition results in fluorescence quenching, PO_4_^3–^ affords a slight enhancement in intensity. On the other hand, in highly polar DMSO, AnSQ exhibits a *λ*_max_ at 464 nm with *Φ*_P_ = 0.158. The presence of either F^–^ or PO_4_^3–^ results in enhancing a quantum efficiency, while OH^–^ and CN^–^ addition gives lower a Stokes shift of *λ*_max_ values to shorter wavelengths, with a bare change in quantum yields.

The results in Fig. 3 and Table 1 highlight the influence of solvent polarity on fluorescence emission. This is highlighted by a comparison of the quantum yields for AnSQ in pure THF (*Φ*_P_ = 0.297) with those obtained in THF/water (95 : 5 v/v, *Φ*_P_ = 0.428). Addition of F^–^ to AnSQ in pure THF results in OFF fluorescence (*Φ*_P_ = 0.004), whilst the same guest in THF/H_2_O results in enhanced fluorescence intensity (*Φ*_P_ = 0.581) and a *λ*_max_ shift to shorter wavelengths. Similarly, AnSQ exposure to CN^–^ and OH^–^ in THF results in quenching, although enhancements occur in THF/H_2_O, mirroring the behavior in DMSO. Although CN^–^ and F^–^ quench AnSQ fluorescence in THF and enhance it in THF/H_2_O, only F^–^ affords fluorescence enhancements in toluene (*Φ*_P_ = 0.394 to 0.975) and DCM (*Φ*_P_ = 0.260 to 0.591). Hydroxide addition to AnSQ in THF results in decrease in fluorescence intensity and lowers a Stokes shift to shorter wavelengths (Δ*λ*_max_= –16 nm), with similar behavior occurring in DMSO. Phosphate, while not measurable in toluene and DCM due to insolubility, affords slight enhancements in AnSQ emission intensity in THF. Additions of PO_4_^3–^ in THF and THF/H_2_O did not provide the different spectrum. Although anion detection of AnSQ in aqueous solution is not feasible due to the precipitation of AnSQ host, use of a binary solvent system in THF/water (95 : 5 v/v) would provide the same trend of results, if it is still homogeneous, such as DMSO and DMSO/water. Only THF and THF/water provided different results.

The differences in the fluorescence behavior of AnSQ may be due to the nature of the excimer formed in each solvent system and on guest exposure, such as solubility and ion-induced molecular and excimer emissions.28*b* For example, poorly soluble solvents might lead to an aggregation of AnSQ, perturbing excimer emission within a SQ cage. A previous report found that approach of an acetonitrile solvent molecule to a SQ cage occurs from the most electronegative center (N atom),^31^ consistent with the findings by Anderson *et al.*^19^ While the approach of negatively charged species to the positive surface of silicon atoms in SQ cage should be favorable, repulsive forces between the guest and lone pairs of oxygen atoms within a T_8_ cage will become prevalent at close distances. Such repulsive forces can result in cage distortion, which in turn alters the geometry of the excimer interactions. Emission from anthracenes can occur from two types of emissive states: excimeric anthracene emission and monomeric anthracene emission.^32^,^33^ The shift between both states can be easily observed by fluorescence spectral changes, in that monomer emission will occur at the lowest Stokes shift or shorter wavelengths while excimer emission wavelength depends on the solvent type.34*a* Addition of fluoride ions to AnSQ results in fluorescence enhancements in all solvents apart from THF, which, being moderately polar, is expected to give rise to charge transfer complex formation. Some increases in fluorescence intensity coincide with lower quantum efficiencies as a consequence of the quantum measurements being based on the emission area to absorption area ratio. As suggested by Narikiyo *et al.*, excimer formation of fluorophores on SQ cages relies on system rigidity, with excimer and molecular fluorescence emission being controllable through the selection of appropriate solvents and guests.34*b*

### Response time

The effects of solvent on the interaction between AnSQ and anionic guests (*e.g.* Cl^–^, Br^–^, NO_3_^–^, F^–^, CN^–^, OH^–^, and PO_4_^3–^) were evaluated on the basis of fluorescence response. Theoretically, an increase in solvent polarity may result in rate enhancements in the case of charged reactants. Reaction rates were measured by addition of 100 equiv. of anions to 6 μM of AnSQ, ensuring pseudo 1^st^ order kinetics. Fluorescence changes were monitored at an excitation wavelength (*λ*_ex_) of 370 nm with maximum emission wavelength fixed according to Table 1. Observations were carried out for 20 min time periods at 30 s intervals, for comparative purposes. Only four anions (*e.g.* F^–^, CN^–^, OH^–^, and PO_4_^3–^) resulted in significant fluorescence intensity changes in high polarity solvents (DMSO, DMF and acetone), in contrast to slow reaction kinetics occurring in low polarity solvents (Table 2). However, F^–^, CN^–^ and PO_4_^3–^ provided fine patterns of change in fluorescence intensity (Fig. S4a and b†). Addition of OH^–^ provided a fluctuating pattern of fluorescent response suggesting that OH^–^ may induce Si–O cleavage and cage rearrangement resulting in several intermediates.9c–e,10b,10e,^11^,27b Other guest anions (Cl^–^, Br^–^ and NO_3_^–^) give rise to only minimal fluorescence responses, as shown in Fig. S4c and d† and Table 1, which change the fluorescence in the same trend, so these three anions cannot be identified with AnSQ.

**Table 2:** Kinetic constants (*k*) highlighting the fluorescence responses of AnSQ to anionic guests (100 equiv.) (*λ*_ex_ = 370 nm)

Solvent	Kinetic constant (*k*) × 10^–3^ s^–1^			
	F^–^	OH^–^	CN^–^	PO_4_^3–^
DMSO	1.3	0.2	0.7	1.3
DMF	2.2	0.3	0.7	0.8
Acetone	2.2	0.5	1.2	0.9
THF + H_2_O	0.5	0.3	0.6	0.3
DCM	1.6	0.3	0.3	0.1
THF	–0.1	0.2	0.5	0.3
Toluene	1.4	0.1	0.7	0.04

### Anion identification

**Fig. 4:**
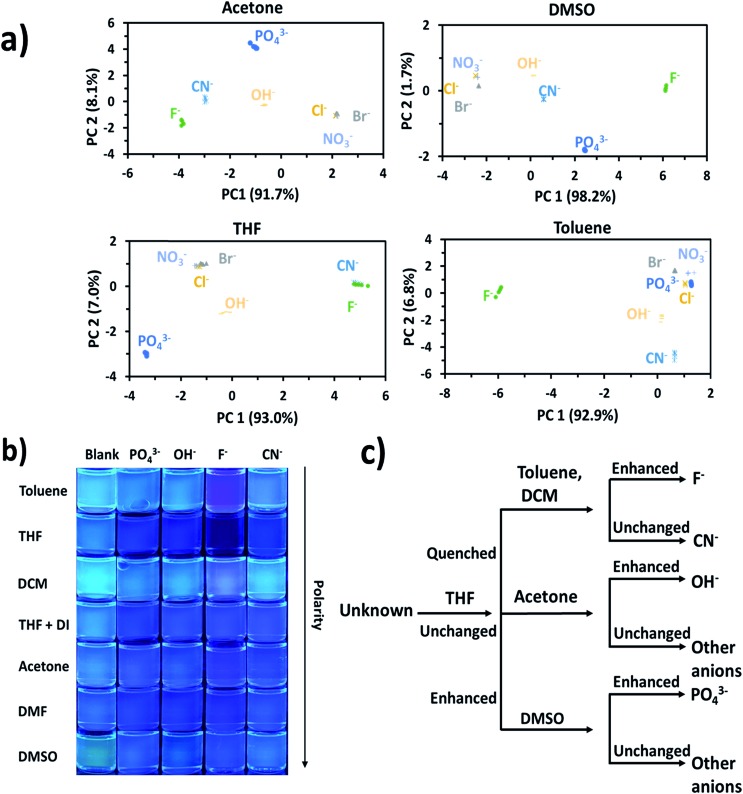
(a) PCA results for AnSQ anion association in different solvents, (b) photographs of AnSQ–guest solution under UV radiation, and (c) dendrogram of AnSQ response behaviour obtained from fluorescence spectra (Table 1).

Principal component analysis (PCA) was used to create association fingerprints for anions with AnSQ.^35^,^36^ Input data were emission spectra obtained at *λ*_ex_ = 370 nm of the sample minus the blank in each solvent (acetone, DCM, DMF, DMSO, THF, THF/H_2_O and toluene). In each solvent, spectra of 6 μM AnSQ were measured before and after addition of 100 equiv. anions (F^–^, OH^–^, NO_3_^–^, CN^–^, PO_4_^3–^, Cl^–^, Br^–^). As in previous experiments, samples were allowed to equilibrate for 18 hours prior to measurement. As expected, spectral changes occurred on anion addition, as shown in Fig. 3. The software Laboratory Virtual Instrument Engineering Workbench (LabVIEW) was used to create PCA graphs. PCA data were obtained from these spectra as the differences in fluorescence intensity (Δ*I*) from 400 to 500 nm over 10 nm intervals, with data being the result of five repeat experiments. The obtained PC contributions were PC1 = 91.7%, PC2 = 8.1% for acetone, PC1 = 98.2%, PC2 = 1.7% for DMSO, PC1 = 93.0%, PC2 = 7.0% for THF and PC1 = 92.9%, PC2 = 6.8% for toluene. The grouping in PC1 for toluene indicates that AnSQ can be used as a sensor for F^–^ in this solvent (see Fig. S6† for PCA data of all anions). So, the combination of PCA and fluorescence changes of AnSQ can be used to create a dendrogram for anion detection as shown in Fig. 4c. In practical use, the unknown anion may be tested with AnSQ in THF first; the obtained spectral change could lead to the next solvent choice. At this state, AnSQ may not be suitable for use with multiple anion solution due to some limitations: notably, that fluoride binds much more strongly than other anions in all solvents, which is considered to be a very competitive anion.

### Quantitative analysis of anion detection

**Fig. 5:**
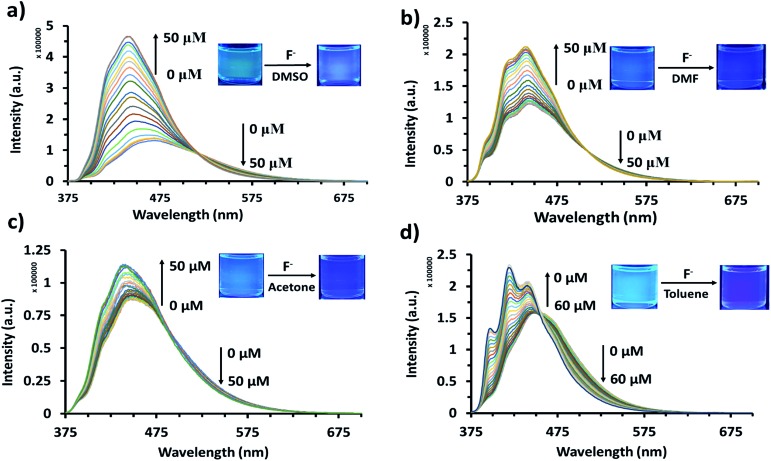
Fluorescence titration of AnSQ (6 μM) with TBAF (*λ*_ex_ = 370 nm) after 2 min of addition for each point in (a) DMSO, (b) DMF, (c) acetone, and (d) toluene after 5 min of addition for each point.

The reaction between AnSQ and anions could presumably occur *via* a coordination complex, as well as the polarity of solvents had affected to the rate of reactions.^37^ Acetone, DMF, DMSO and toluene were selected as solvents for the determination of detection limits due to the fast kinetics of anion association (Fig. 5 and S7†). For AnSQ (6.0 μM) in DMSO, the detection limit for F^–^ detection was 1.001 ppb. Responses to other anions were less marked: 5.61, 2.04 and 4.54 ppb for OH^–^, CN^–^ and PO_4_^3–^, respectively (Fig. S10†). In DMF for the same AnSQ concentration, detection limits were higher (1.65, 26.9, 12.1 and 59.5 ppb) for F^–^, OH^–^, CN^–^and PO_4_^3–^, respectively. In acetone, AnSQ detection limits for all anions apart from PO_4_^3–^ (10.2 ppb) were higher than in DMF, and in toluene only F^–^ (9.57 ppb) showed good sensitivity. Thus solvent polarity is an important factor affecting anion binding, and these results afford useful insights into solvent selection for detection of anionic analytes.

**Fig. 6:**
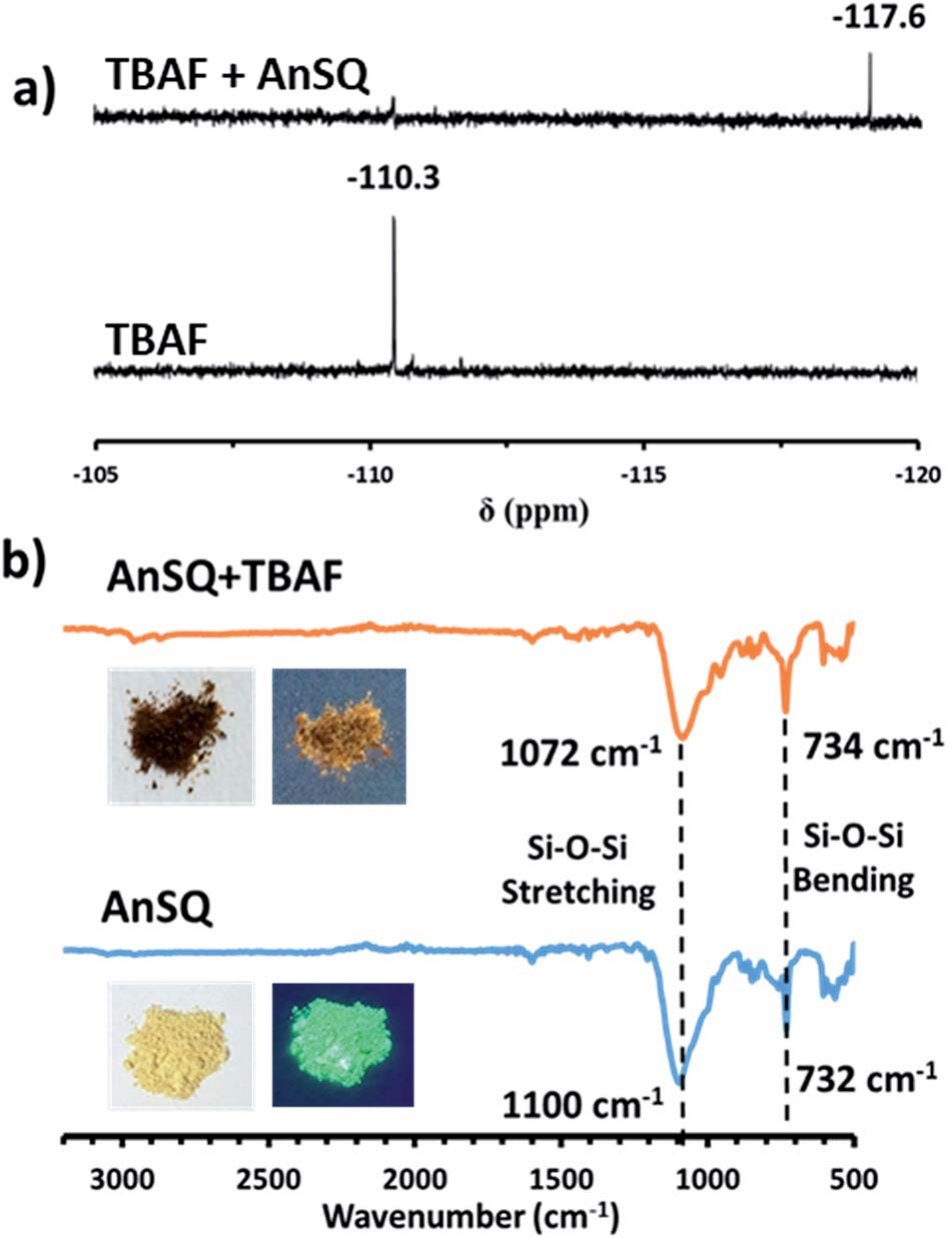
(a) ^19^F NMR spectra for titration of AnSQ with TBAF using DMSO-*d*_6_ as the solvent and CFCl_3_ as a calibration agent, (b) FTIR spectra of AnSQ before and after addition of TBAF (1 equiv.) with inset pictures of AnSQ under visible (left) and UV lights (right).

Results in Fig. 5 and S7† indicate that, with anion addition, molecular emission intensity increases at the expense of excimer emission. Anion binding strengths, as calculated from Benesi–Hildebrand plots from titration data (Fig. S9†), were 3333, 173, 263 and 697 M^–1^ for F^–^, OH^–^, CN^–^ and PO_4_^3–^, respectively. Selectivity for F^–^ can be explained on the basis of cage encapsulation, as confirmed by ^19^F NMR experiments (Fig. 6a). Addition of TBAF to AnSQ in DMSO-*d*_6_ results in substantial upfield chemical shift changes of the F^–^ resonance, relative to that of uncomplexed TBAF. So, the chemical shift further upfield in the ^19^F NMR spectra and the outstanding binding constant can confirm the encapsulation of fluoride in the cage.

**Fig. 7:**
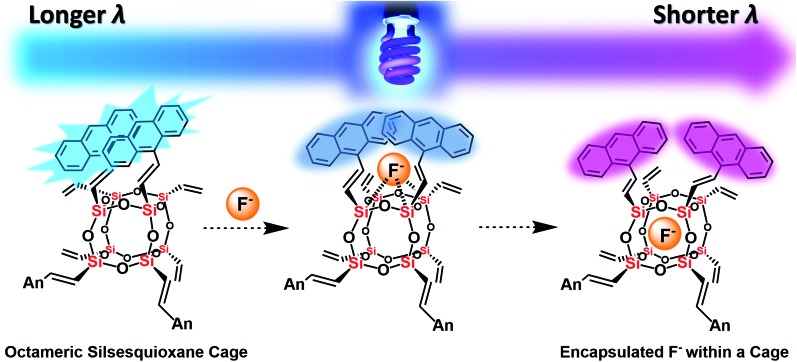
The plausible mechanism of fluoride capture by AnSQ involving facial interaction followed by encapsulation and its fluorescent characters.

FTIR result (Fig. 6b) of AnSQ in the solid state shows the key signal of Si–O–Si stretching vibrational frequency at 1100 cm^–1^, confirming the existence of a cage framework. Upon addition of 1 equiv. TBAF, an absorption band associated with Si–O bond stretching of AnSQ appears in the lower energy at 1072 cm^–1^. This result suggested the formation of [AnSQ + F^–^] complex through very strong host–guest interactions as the Si–O bond strength decreases, while Si–F bonds form. Solution studies also suggest that only fluoride is thermodynamically capable of being encapsulated by the cage (Fig. 7), whereas other anions may interact solely with the cage surface. This is because the Si–O–Si stretching vibrational frequency of AnSQ diplays minor changes with all anions except fluoride as shown in Fig. S11† confirming weaker host–guest interactions at the SQ cage.28*c*

### UV-vis spectroscopy

**Fig. 8:**
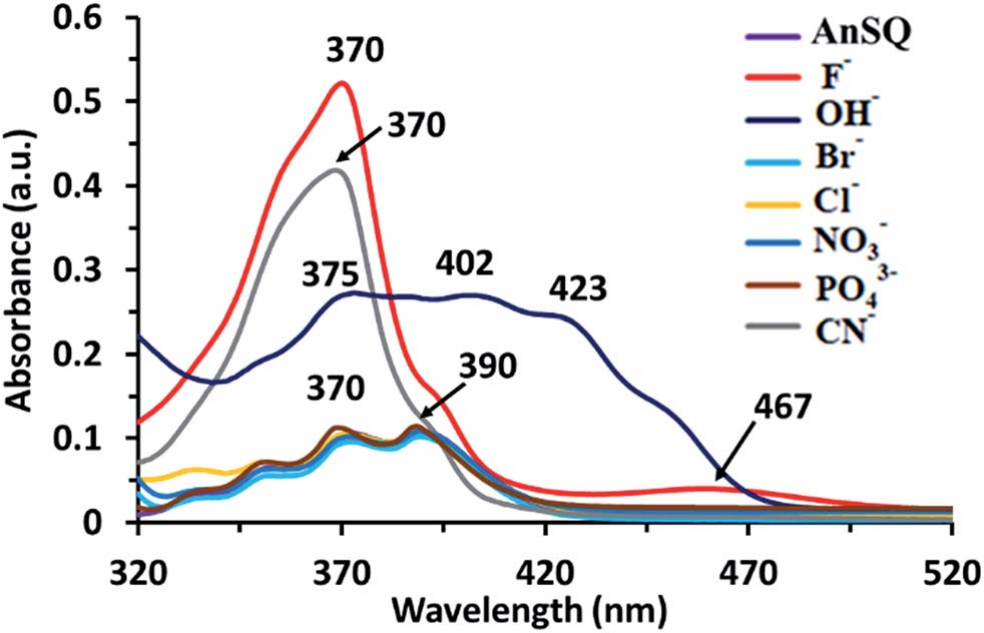
UV absorptions of AnSQ (6 μM) in THF before and after anion addition at 18 hours (100 equiv.).

**Fig. 9:**
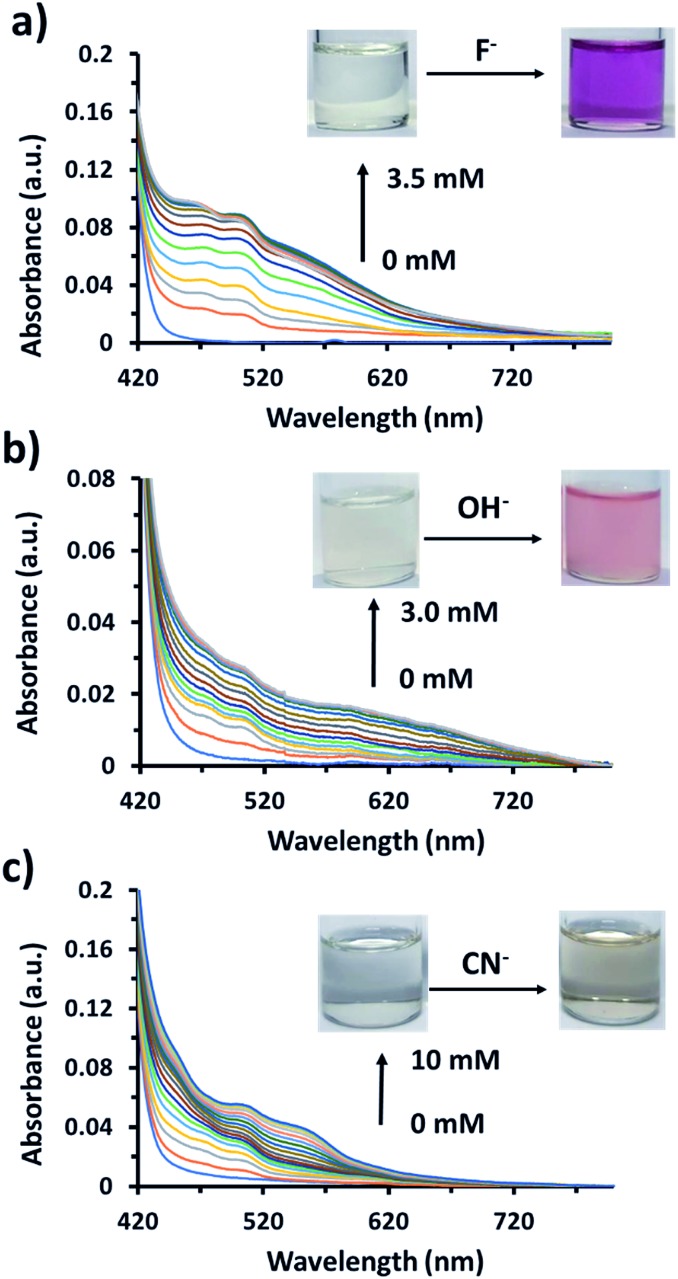
UV-visible titration of 60 μM of AnSQ in THF upon addition of (a) fluoride, (b) hydroxide, and (c) cyanide ions within 2 min of addition for each point.

UV-visible absorption measurements of AnSQ in THF with various anions supported the change of anthracene excimer type on association (Fig. 8). 100 equiv. of the anions was added to 6 μM of AnSQ and left for 18 hours. The UV-vis absorption spectra of AnSQ are most perturbed in the presence of F^–^, CN^–^ and OH^–^, as a consequence of excimer changes.^29^ Absorbance of AnSQ shows two major peaks at *λ*_abs_ = 370 and 390 nm, and after addition of F^–^ and CN^–^ the peak at *λ*_abs_ = 370 nm alone predominates, while *λ*_abs_ = 375, 402 and 423 nm are prevalent on OH^–^ addition. The flexibility of SQ allows for distortion to occur on anion association, giving rise to various types of anthracene excimers as reflected by the number and position of absorption bands. These absorptions can provide insights into anion discrimination by host systems.^25^,^29^ Interestingly, absorbance bands in the visible region for OH^–^ show a red shift to *λ*_abs_ = 402 and 423 nm, while F^–^ addition also results in visible light absorbance at 467 nm. This suggests that F^–^, CN^–^ and OH^–^ led to reorganize of SQ framework forming charge transfer complexes; such complexes have been documented for anthracene itself in mid-polarity solvents.^38^ SQs appended with aromatic motifs are able to form charge transfer complexes, both ligand to ligand and ligand to cage types.^39^,^40^ This is evident by the naked eye detection of F^–^ and OH^–^ by AnSQ in THF, as indicated by changes in absorption in the visible region.^21^ While charge transfer complex formation in SQs can occur in the absence of anions, only F^–^, OH^–^, and CN^–^ were found to induce charge transfer complex formation in AnSQ, as evident by their purple, pink, and yellow solution colors (Fig. 9). The results from UV-vis titration of AnSQ with F^–^, OH^–^ and CN^–^ showed limits of detection being 0.452, 1.01 and 1.56 ppm respectively (Fig. S8†).

### Computational studies

As a starting point the pristine molecular T_8_ structure with hydrogen atoms as R groups was created using ArgusLab chemistry software, for use in Gaussian 03. Geometry optimizations of pristine T_8_ were done using Gaussian 03 incorporating density functional theory (DFT) including B3LYP with the LanL2MB basis set. After optimization, the cubic T_8_H_8_ structure was obtained, and the H atoms replaced with 9-vinyl anthracenes, creating the T_8_R_8_ framework. Electrostatic potential surface calculations were done utilizing density functional theory (DFT) including B3LYP with the LanL2MB basis set (Fig. S5†). Structural and energy optimizations of SQs were achieved by MM2 minimization with free bond rotation and ion mobility, while minimizing steric hindrance. Eight 9-vinyl anthracenes peripheral SQ (T_8_R_8_) was used as a representative to be the ideal molecule for studying the excimer behaviour.

**Fig. 10:**
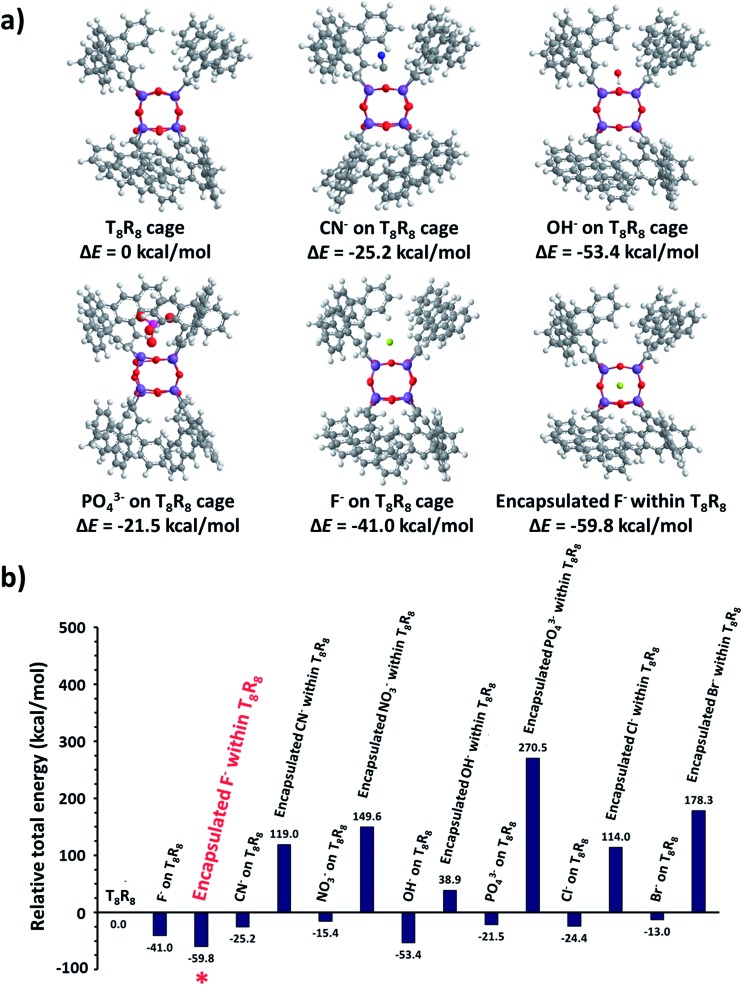
(a) Potential host–guest anion complex formation by T_8_R_8_ as determined by computational molecular modelling and (b) the relative total energy (Δ*E*) of complexes in comparison with that of T_8_R_8_.

Electrostatic potential calculations indicate that the surface of T_8_R_8_ has a positive contour, providing the opportunity for anions to approach the cage.^19^ The anthracene excimer formations in T_8_R_8_, as studied by MM2 minimization with no constraints on ion movement or bond rotation but including minimization of steric hindrance, were performed by placement of anions (F^–^, Cl^–^, Br^–^, OH^–^, CN^–^, PO_4_^3–^, NO_3_^–^) either on the cage surface or in the interior. As shown in Fig. 10 the T_8_R_8_ cage responded well in the case of encapsulated F^–^ or surface placed OH^–^ (relative total energies –59.74 and –53.31 kcalmol^–1^, respectively). Fluoride encapsulation thus involves at least 2 steps, formation of a kinetic product from the facial anion approach, and subsequent encapsulation affording the thermodynamic product (Fig. 10).^21^ The relative energies of anion-cage association events are shown in Fig. 10a and b and in surface association the results are in accordance with those of Anderson *et al.*^19^ with the addition of anions resulting in cage stabilization relative to pristine T_8_R_8_, as reflected by the negative total energies. The AnSQ minimalization studies suggest that cage distortion occurs as a consequence of two interactions: attraction between the positive surface and anionic guest, and repulsion between anions and T_8_ oxygen lone pairs. These forces thus influence anthracene excimer formation as a result of cage distortion and excimer rearrangement (Fig. 10). As suggested by Narikiyo *et al.*, the emission of fluorophores on SQs could be controlled by solvent polarity,34*b* but for more rigid SQ systems such as AnSQ emission changes also result from anion association. Anion association by AnSQ promotes molecular emission at the expense of excimer emission.

Valiev *et al.* had done the calculation on anthracene excimers. Briefly, the formation of anthracene excimers is classified into 5 types: slipped-parallel (off-set), graphite-like, T-species, X-species and end-overlapped.^26^,37b By the minimalization with no solvation, the results show that, even with no solvent effect, the addition of anions to the T_8_ cage can change from graphite-like to slipped-parallel or end-overlapped, which can explain the increase of fluorescence after anion addition in most cases of AnSQ. In practice when the solvent effects are also involved, the conformation of anthracene formation could be simply detected by the UV spectroscopy technique. The UV-vis absorption measurement of AnSQ is in agreement with those calculations. In THF, the addition of F^–^ and CN^–^ changed the UV-vis absorption from multiple peaks into one dominant peak at *λ*_max_ = 370 nm (Fig. 8), which relates to the change from slipped-parallel into graphite-like formation which has more π–π stacking, providing quenching emission. In higher polar solvents such as DMF, the UV-vis absorption of AnSQ has also increased upon anion addition indicating the formation of charge transfer complexes among anthracene units on AnSQ, in other words it changes from off-set into end-overlapped or non-excimer (Fig.S8a†).

**Fig. 11:**
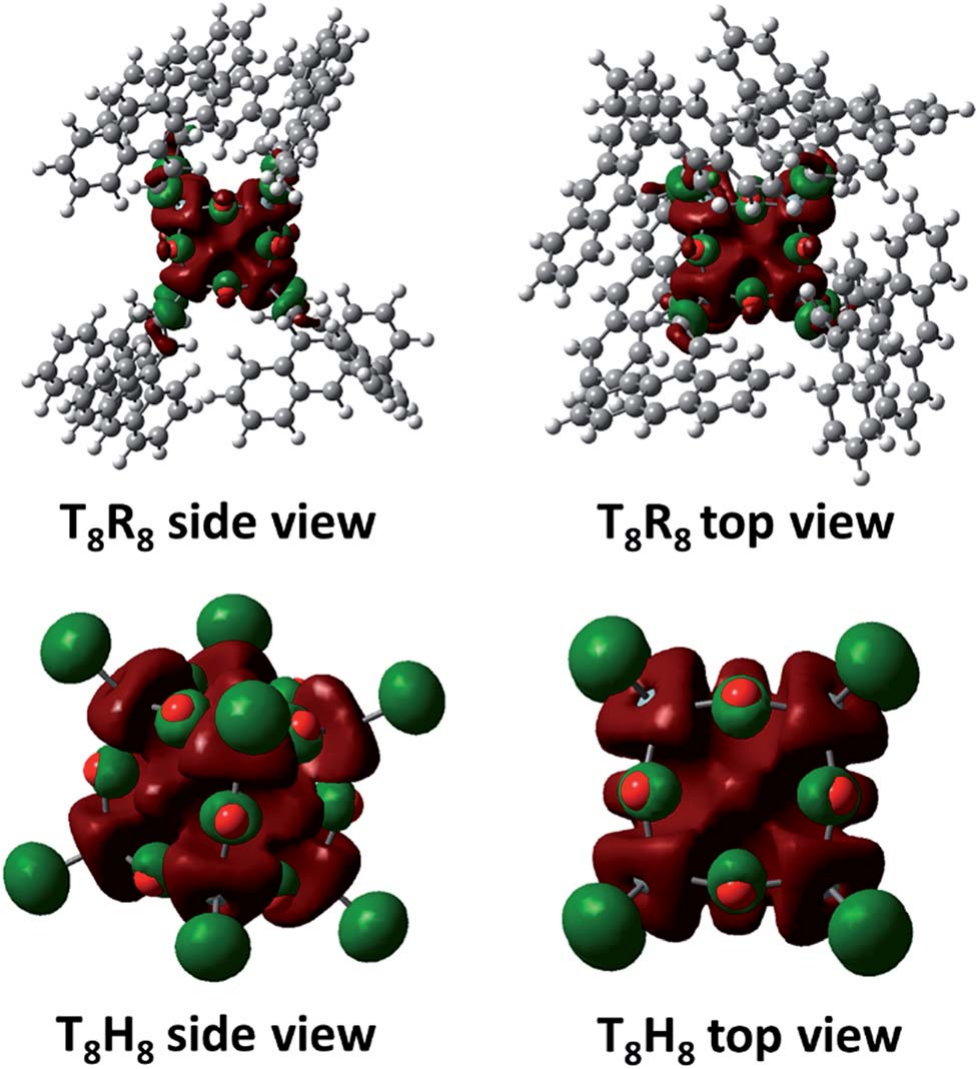
LUMO of T_8_R_8_ and T_8_H_8_ from DFT calculation.

It is worth mentioning that the LUMO state of conjugated T_8_ cages with fluorophores is highly electron deficient.^41^–^43^ The calculated LUMO of T_8_R_8_ and T_8_H_8_ also pointed out of a cage (Fig. 11), which is the reason that it is possible to hypothesize about interactions between anions and SQ cages. As suggested by the results in this work, anions such as F^–^, OH^–^, CN^–^ and PO_4_^3–^ would interact through the surface of the SQ cage, but only fluoride could be thermodynamically trapped inside the T_8_R_8_ cage.

## Conclusions

Anthracene-conjugated octameric silsesquioxane (AnSQ) cages were successfully prepared through Heck coupling reactions. These materials strongly exhibited the large Stokes shift of fluorescence emission behavior, suggesting the presence of intramolecular excimer formation. The emission intensity of AnSQ and the quantum yield show inverse relationships to solvent polarity. Kinetic studies of AnSQ fluorescence response on exposure to anions (F^–^, CN^–^, OH^–^, and PO_4_^3–^) indicate that association is more rapid in polar solvents such as DMSO, DMF and acetone than in less polar solvents. Other halide and nitrate ions, however, show no significant response in any of the solvents. Both F^–^ and CN^–^ addition results in fluorescence quenching in THF, although the presence of fluoride enhances fluorescence in all other solvents. Phosphate enhances fluorescence only in highly polar solvents such as DMSO, DMF, and acetone, whilst OH^–^ response is only enhanced in low polarity solvents (*e.g.* DCM and toluene). Emission spectral changes depend on anion association and solvent polarity, which cause shifting between monomer and excimer emission modes. Such pathways of anionic identification are rationalized by a dendrogram, formulated by principal component analysis using fluorescence intensities, anions, and solvents, as variants. Changes in the excimer type on anion addition indicated that only F^–^, OH^–^, and CN^–^ association with AnSQ facilitated naked eye detection in THF (*λ*_abs_= 498, 471 and 500 nm), respectively. These results confirmed the formation of charge transfer complexes among anthracene units in AnSQ. Electrostatic potential surface mapping of AnSQ provided a rationale for anion attraction to the cage, causing the excimer distortion with minimization affording relative total energies of anion–host combinations. Of these, only encapsulation of F^–^ was deemed feasible on energy grounds, with other anions exhibiting cage surface interactions.

This study confirmed the feasibility of functionalization of SQ with four anions in fluorescence mode and three anionic species by the naked eye. Using the SQ core as a molecular sensor thus opens up many possibilities for the development of modern functional anion recognition systems. Furthermore, the behavior of AnSQ toward fluoride in THF, with lowering of fluorescence intensity but production of intense purple color confirmed the formation of SQ charge transfer complexes. Further studies on these complexes are currently underway.

## Conflicts of interest

There are no conflicts of interest to declare.

## Supplementary Material

Supplementary information
