# Supplementary material for: Anion identification using silsesquioxane cages
Source: Chem Sci. 2018 Sep 25;9(40):7753–65. doi: 10.1039/c8sc02959h (PMC6194494; doi:10.1039/c8sc02959h)
Supplement: Supplementary file 1 [file SC-009-C8SC02959H-s001.pdf]

Electronic Supplementary Material (ESI) for Chemical Science.
This journal is © The Royal Society of Chemistry 2018

# Electronic Supplementary Information (ESI)

# Anion Identification using Silsesquioxane Cages

Supphachok Chanmungkalakul,[a] Vuthichai Ervithayasuporn,*[a] Patcharaporn Boonkitti,[a] Alisa Phuekphong,[a] Nicha Prigyai,[a] Sumana Kladsomboon,[b] and Suda Kiatkamjornwong[c,d]

[a] *Department of Chemistry, Center of Excellence for Innovation in Chemistry (PERCH-CIC), and Center for Inorganic and Materials Chemistry, Faculty of Science, Mahidol University, Rama VI Road, Ratchathewi, Bangkok 10400, Thailand.*

[b] *Department of Radiological Technology, Faculty of Medical Technology, Mahidol University, Nakhon Pathom 73170, Thailand.*

[c] *Faculty of Science, Chulalongkorn University, Phayathai Road, Bangkok 10330, Thailand.*

[d] *FRST, Division of Science, the Royal Society of Thailand, Sanam Suepa, Dusit, Bangkok 10300, Thailand.*

**Corresponding Author: Email: vuthichai.erv@mahidol.ac.th; vuthichai.erv@mahidol.edu (V.E.)*

# Table of contents

Pages

Chemicals and Instruments……………………………….……………S3

Synthesis of AnSQ……………………………………………………..S4

MALDI-TOF Spectrum of AnSQ…………….....……………………..…S6

Pictures of the solid substances …………………………….....…….S7-8

Quantum yield measurements …………………………….…………S9-36

Kinetic studies ………………………………………….……...…S37-40

Electrostatic potential surface …………………….………………….S41

Principle component analyses ………………………………………..S42

Fluorescence Titrations…………………….…………………………..S43-55

UV-Vis Titrations……………………......……………………….S56-59

Benesi-Hildebrand plots……………………..…………….……S60-61

Calibration curves…………………………………...………….…S62-70

FTIR of solid state AnSQ ……………………………….......S71-74

## Chemicals and Instruments

9-Bromoanthracene, $PPh_3$, triethylamine, tetrabutylammonium salts and $Pd(OAc)_2$ were purchased from TCI Chemicals and Sigma Aldrich, while octavinylsilsesquioxane (OVS) was prepared according to a previous literature report.[1] The list of chemicals suppliers are provided table S1. Deionized (DI) water was obtained from Ultra Clear SIEMENS with ASTM type 2. The commercial solvents of acetone, DCM, were further distilled. The AR grade of THF, Toluene, DMF and DMSO were used without purification. The silica gel (No. 60) used for column chromatography were purchased from Merck&Co., Inc. $^{19}$F NMR results were obtained using a Bruker-AV 400 high-resolution magnetic resonance spectrometer. FT-IR spectra were recorded using the attenuated total reflectance (ATR) technique on a Bruker model Alpha spectrometer. High-performance MALDI-TOF analysis was performed on a Bruker autoflexTM series instrument. UV-Vis spectroscopy was performed on a UV-Vis spectrophotometer (Shimadzu UV-2600), while all fluorescence spectra were recorded using a spectrofluorometer (Horiba FluoroMax4+, integration time 0.1 s, slit width 2 nm). These conditions were also employed for measurement of quantum yields, although such measurements required the use of a BaO coated spherical cube.

**Table S1. List of chemicals**

| Chemicals | Chemical formula | Suppliers |
|---|---|---|
| 9-Bromoanthracene | $C_{14}H_9Br$ | Tokyo Chemical Industry |
| tetrabutylammonium bromide (TBABr) | $(C_{16}H_{36}N)Br$ | Tokyo Chemical Industry |
| tetrabutylammonium chloride (TBACl) | $(C_{16}H_{36}N)Cl$ | Tokyo Chemical Industry |
| tetrabutylammonium cyanide (TBACN) | $(C_{16}H_{36}N)CN$ | Tokyo Chemical Industry |
| tetrabutylammonium fluoride (TBAF) | $(C_{16}H_{36}N)F$ | Sigma Aldrich |
| tetrabutylammonium nitrate ($TBANO_3$) | $(C_{16}H_{36}N)NO_3$ | Tokyo Chemical Industry |
| tetrabutylammonium phosphate ($TBAPO_4$) | $(C_{16}H_{36}N)_3PO_4$ | Tokyo Chemical Industry |
| tetrametylammonium hydroxide (TBAOH) | $(C_4H_{12}N)OH$ | Sigma Aldrich |
| Triethylamine | $C_6H_{15}N$ | Tokyo Chemical Industry |
| Triphenylphosphine | $P(C_6H_5)_3$ | Tokyo Chemical Industry |
| Palladium(II) acetate | $Pd(OAc)_2$ | Tokyo Chemical Industry |

## Reference

1. Harrison, P. G., & Hall, C. (1997), Main Group Metal Chemistry, 20(8), 515–530.

## Synthesis of anthracene-conjugated octameric silsesquioxane cages (AnSQ)

The AnSQ cage was prepared using a Heck coupling methodology. An oven-dried (100 $^{o}$C) 100 ml 2-necked round bottom flask was charged with 632 mg of OVS (1 mmol), 90 mg of palladium (II) acetate (0.4 mmol) and 210 mg of triphenylphosphine (0.8 mmol) along with a stirring magnetic bar. After evacuation for 30 minutes, an argon flow was introduced, followed by a THF/$Et_3N$ (30/15 ml) solution. After bubbling argon through the solution for 30 minutes, a separately prepared solution of 3.217 g (12 mmol) of 9-bromoanthracene in 15 ml of dry THF was injected into the mixture. The reaction was stirred and heated at 80 $^{o}$C for 48 hours. Work-up involved cooling to room temperature and removal of insoluble catalyst by filtration. The clear solution mixture was diluted with 200 ml of 0.5% HCl in MeOH to remove excess base, affording a precipitate which was collected and re-dissolved in the minimum volume of $CH_2Cl_2$. Flash column chromatography on silica gel using 100% $CH_2Cl_2$ as eluent afforded a residue that was purified by Soxhlet extraction, first with MeOH and then with hexane (one day per extraction). The bright yellow solid obtained 1.76 g (86 % yield) was stored under inert atmosphere in the refrigerator.

# Quantum efficiency measurement

The quantum efficiencies were measured by comparing between solvents as a blank and sample according to this equation (original from Horiba with sphere cuvette correction):

$$\phi_{\mathrm{P}} = \frac{\Delta \text{ Area under emission curve}}{\Delta \text{ Area under absorption curve}}$$

the default mode for quantum yield measurement was set at slitwidth 3 nm, integration time 1 sec and increment of emission 3 nm per step. After that parabolic spherical barium oxide was equipped to cover the quartz cell. This quantum efficiency measurement does not require reference, except for calibration using Rhodamine B. 100 equiv. of anions were added into 6 μM of AnSQ and wait 18 hours before measurement to reach the equilibrium (Fig S3, table 1).

## Quantitative Analysis of Anion Detection

A polar solvent, DMSO, was selected for anion association studies due to high complex stability, fast formation kinetics and strong excimer emission in this medium. Binding, or association, constants ($K_a$) were calculated using the Benesi-Hildebrand equation as below.

(a) for fluorescence studies and (b) for absorption studies

$$\frac{1}{I-I_0} = \frac{1}{I'-I_0} + \frac{1}{(I'-I_0)K_a[A^-]} \quad (a)$$

$$\frac{1}{A-A_0} = \frac{1}{A'-A_0} + \frac{1}{(A'-A_0)K_a[A^-]} \quad (b)$$

In equations (a) and (b), $I_0$ and I refer to fluorescence intensities prior to, and post-anion addition. Likewise, $A_0$ and $A$ refer to absorbance values prior to, and post-anion addition.

Limits of detection (LOD) and quantitation (LOQ) were obtained from titration results and calculated from the linear region using the following equations, with $S_b$ referring to the standard deviation.

The standard deviation ($S_b$) can be calculated by the below equation, where $S$ is the slope of the calibration curve.

$$S_b = \frac{s_{y/x}}{\sqrt{\sum_i (x_i - \bar{x})^2}}, \; S_{y/x} = \sqrt{\frac{\sum_i (y_i - \bar{y})^2}{n-2}}$$

In this work, the $x$ value refers to the concentration of added anions, with y reflecting the fluorescence intensity and absorbance values.

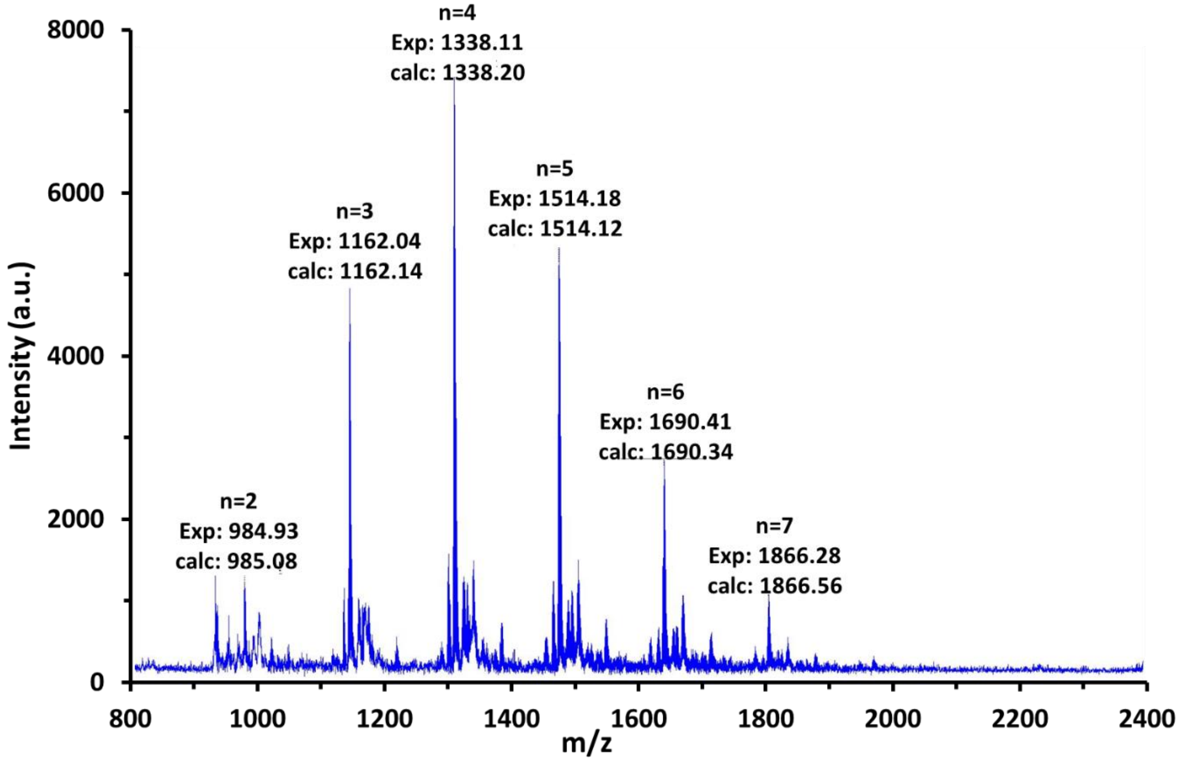


**Figure S1.** MALDI-TOF spectrum of AnSQ $[M+H]^+$ from OVS : 9-bromoanthracene = 1 : 12 *via* heck coupling reaction.

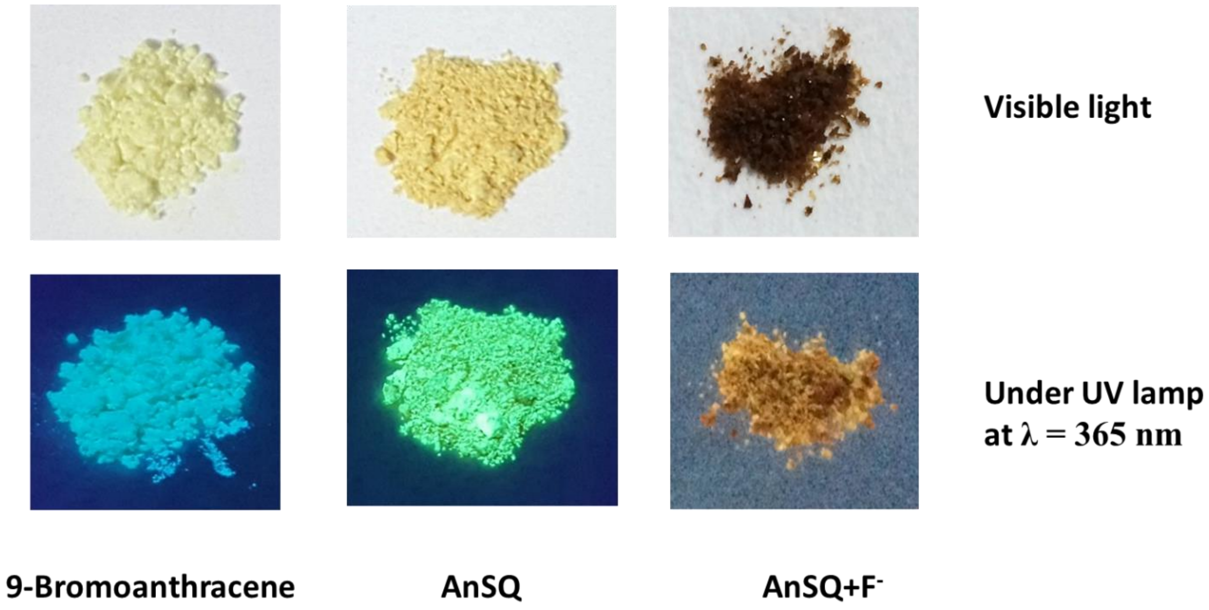


**Figure S2a**. Solid state of 9-bromoanthracene, AnSQ and AnSQ after fluoride addition at solid state under visible light and UV lamp PL series handheld split-tube lamp, model UVGL-55 at $\lambda_{ex}$ = 365 nm

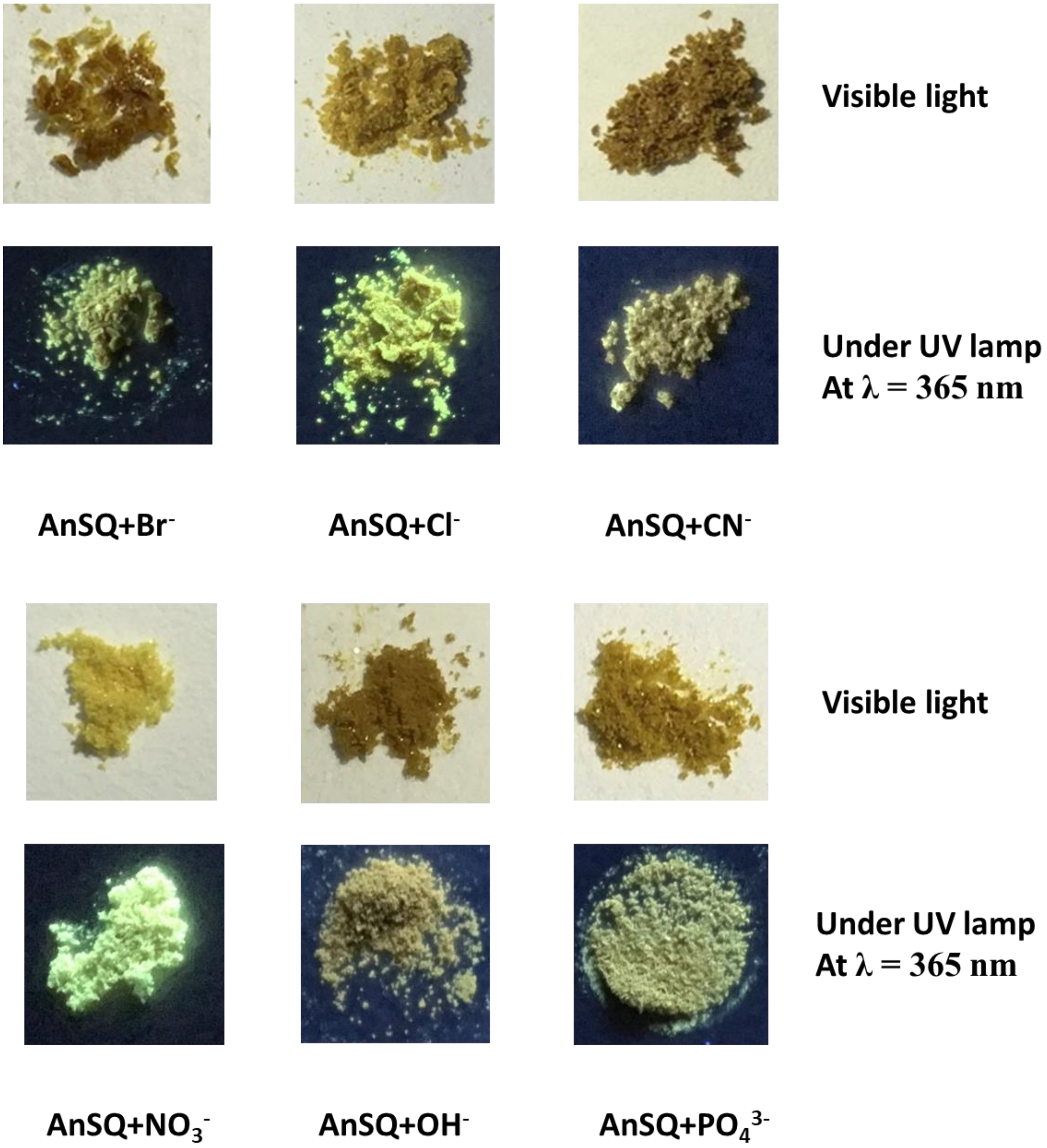


**Figure S2b.** AnSQ and AnSQ anions additions at solid state under visible light and UV lamp PL series handheld split-tube lamp, model UVGL-55 at $\lambda_{ex}$ = 365 nm

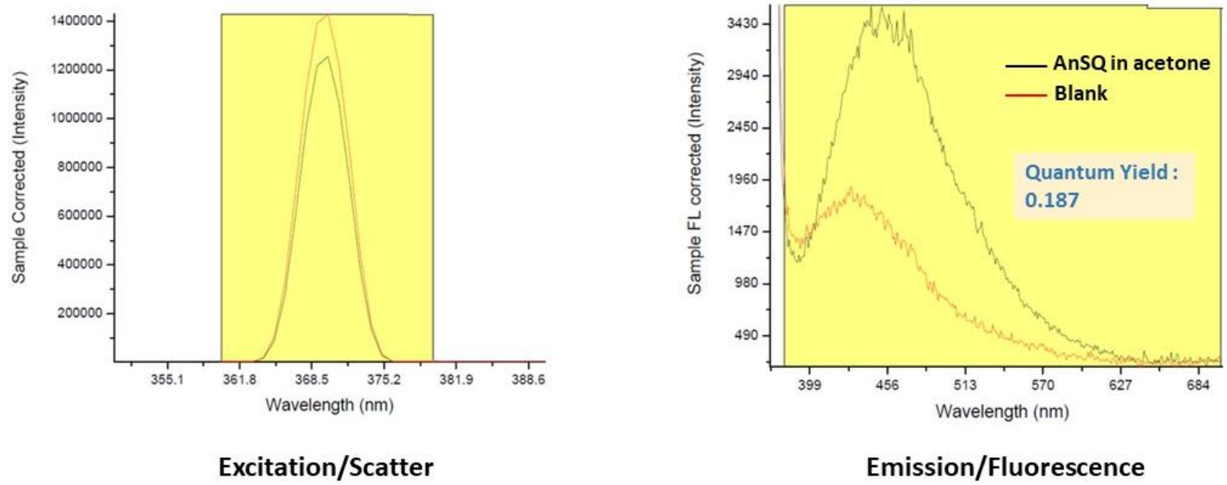


**Figure S3aa.** Quantum yield of AnSQ in acetone

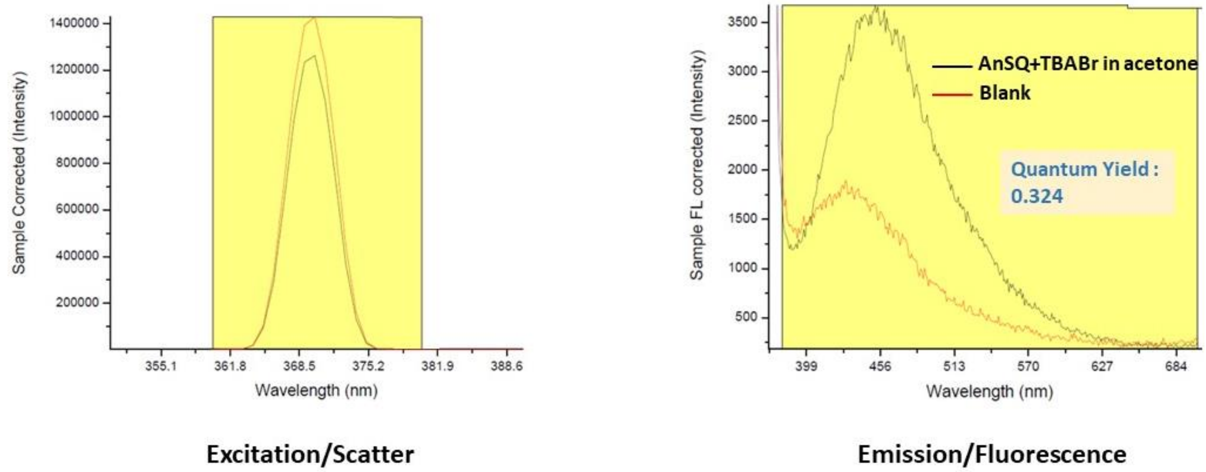


**Figure S3ab.** Quantum yield of AnSQ-$Br^-$ in acetone

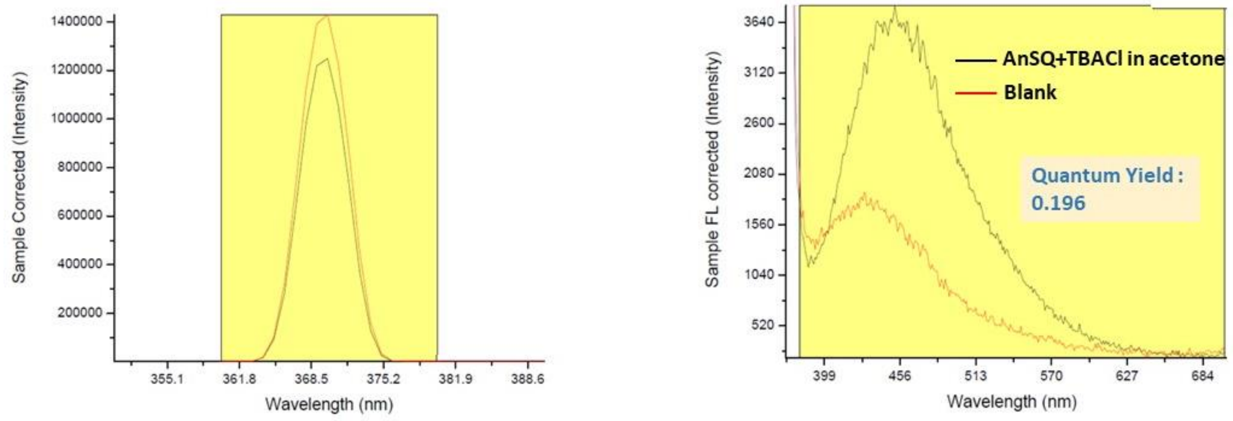


Excitation/Scatter

Emission/Fluorescence

**Figure S3ac.** Quantum yield of AnSQ-Cl$^-$ in acetone

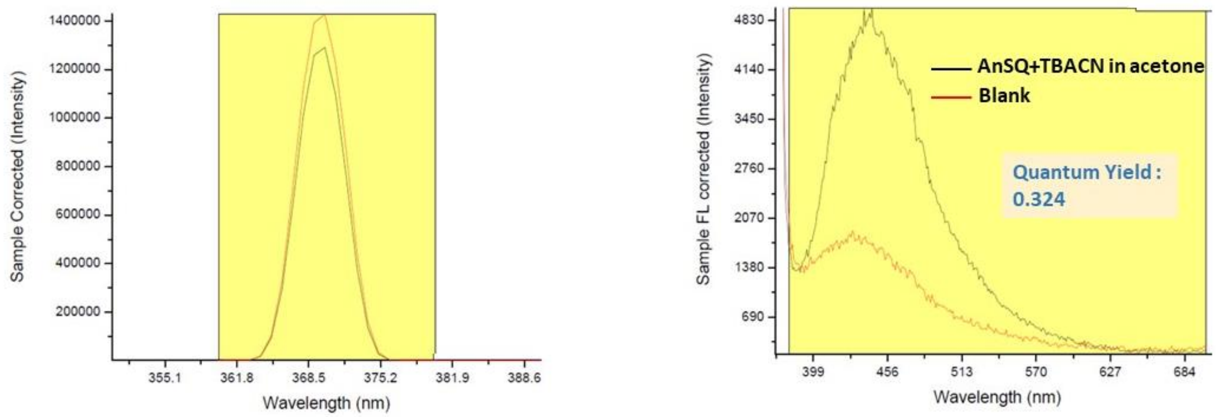


Excitation/Scatter

Emission/Fluorescence

**Figure S3ad.** Quantum yield of AnSQ-CN$^-$ in acetone

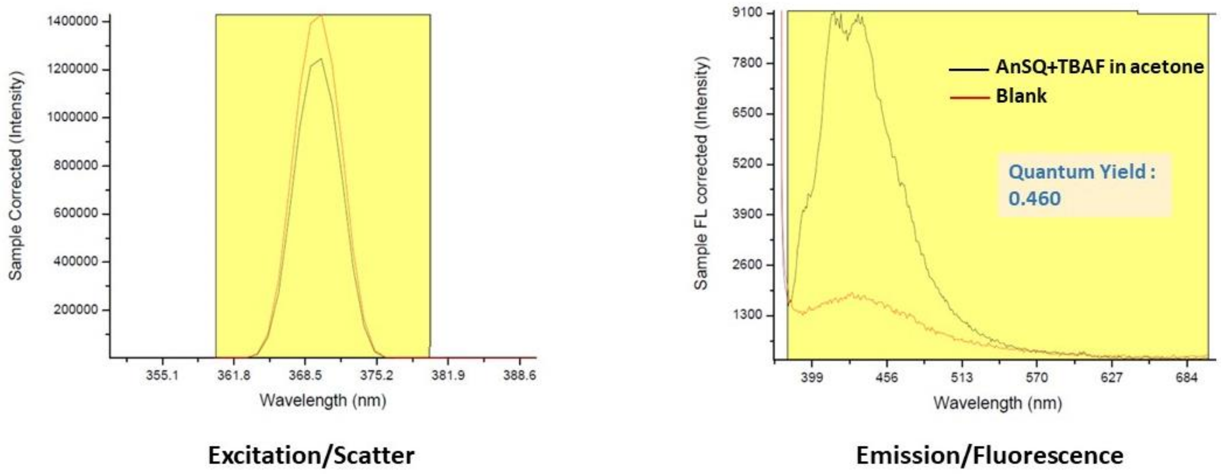


**Figure S3ae**. Quantum yield of AnSQ-$F^-$ in acetone

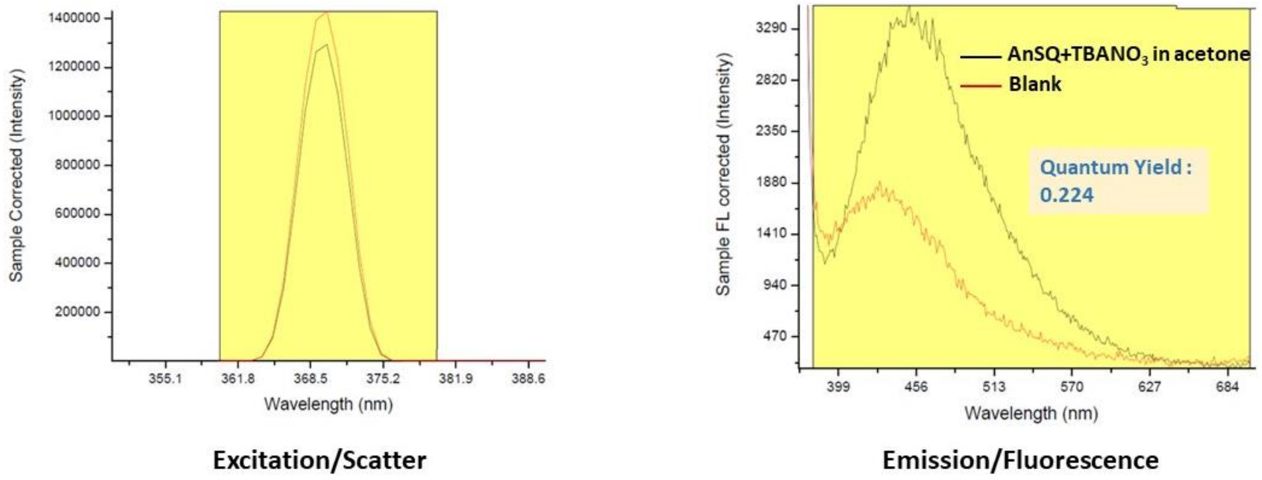


**Figure S3af.** Quantum yield of AnSQ-$NO_3^-$ in acetone

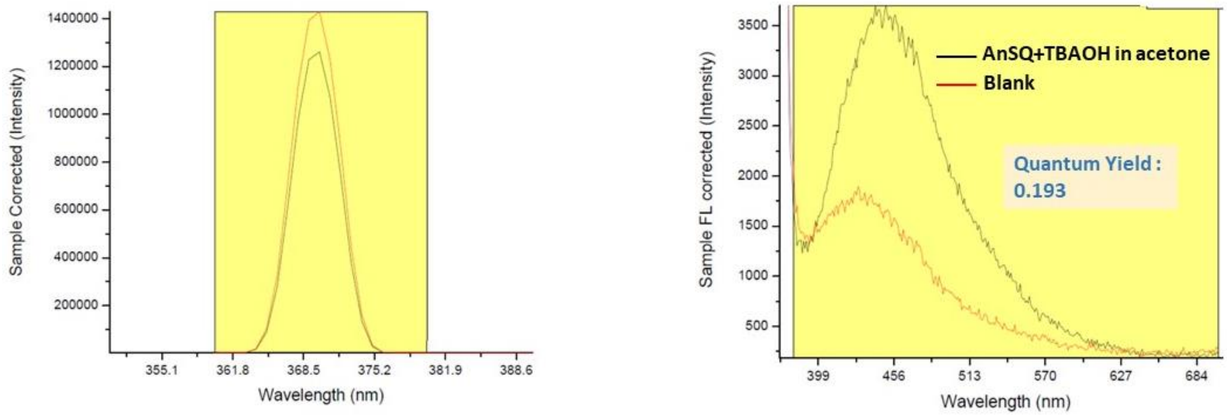


**Excitation/Scatter** **Emission/Fluorescence**

**Figure S3ag.** Quantum yield of AnSQ-$OH^-$ in acetone

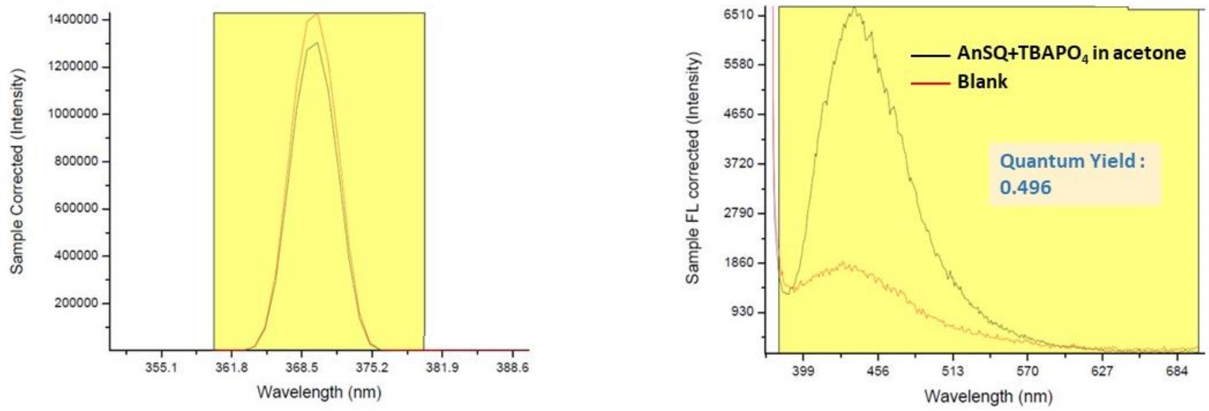


**Excitation/Scatter** **Emission/Fluorescence**

**Figure S3ah.** Quantum yield of AnSQ-$PO_4^{3-}$ in acetone

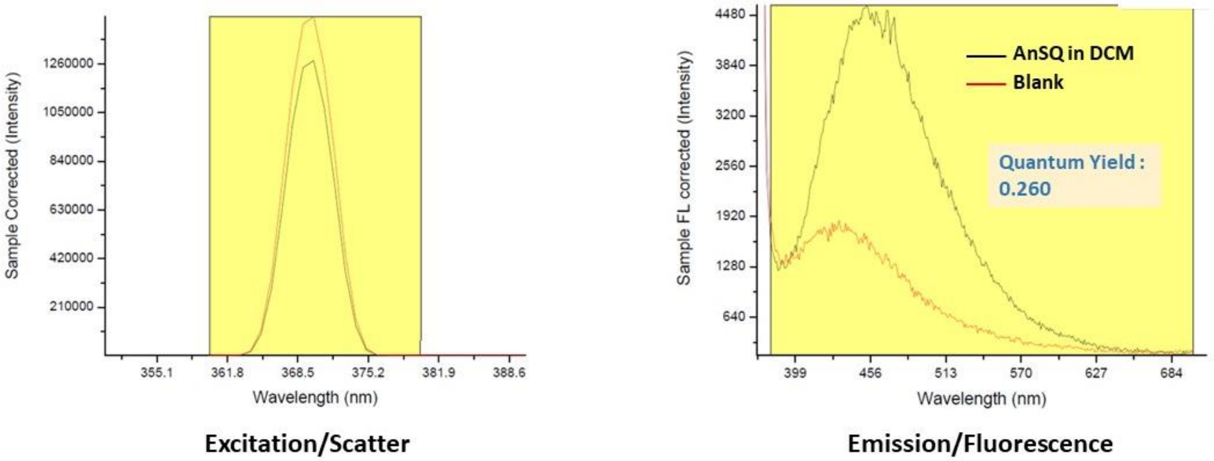


**Figure S3ai.** Quantum yield of AnSQ in DCM

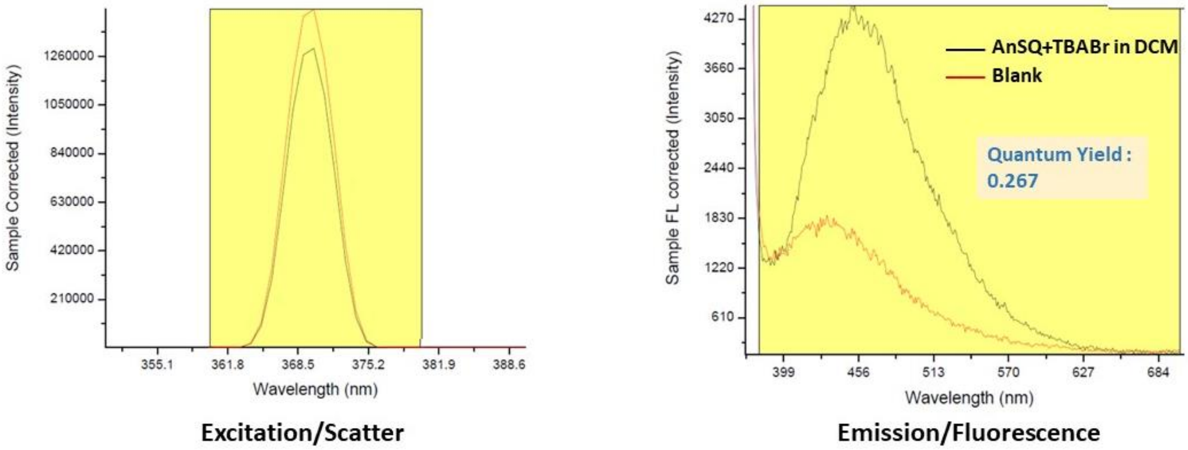


**Figure S3aj.** Quantum yield of AnSQ-$Br^-$ in DCM

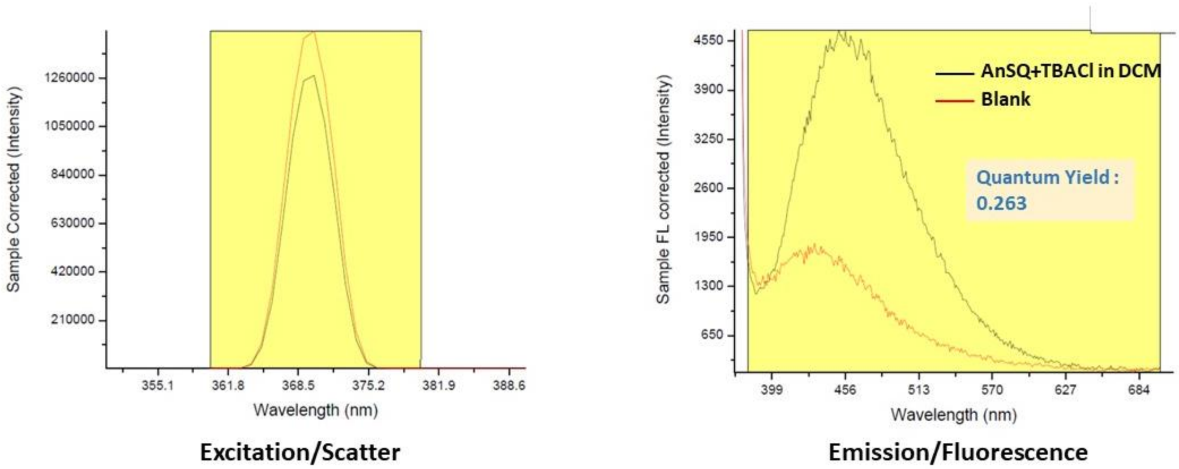


**Figure S3aj.** Quantum yield of AnSQ-$Cl^-$ in DCM

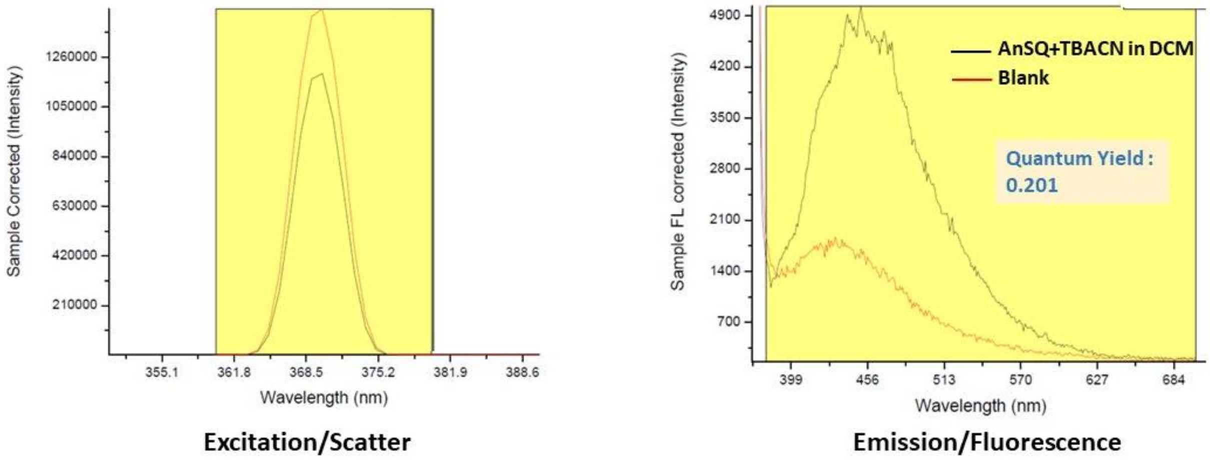


**Figure S3ak.** Quantum yield of AnSQ-$CN^-$ in DCM

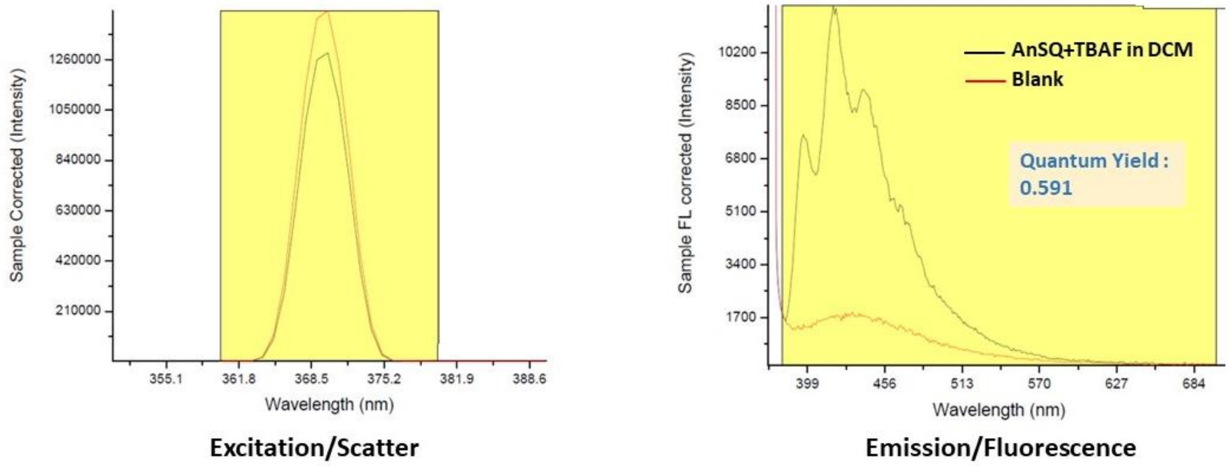


**Figure S3al.** Quantum yield of AnSQ-$F^-$ in DCM

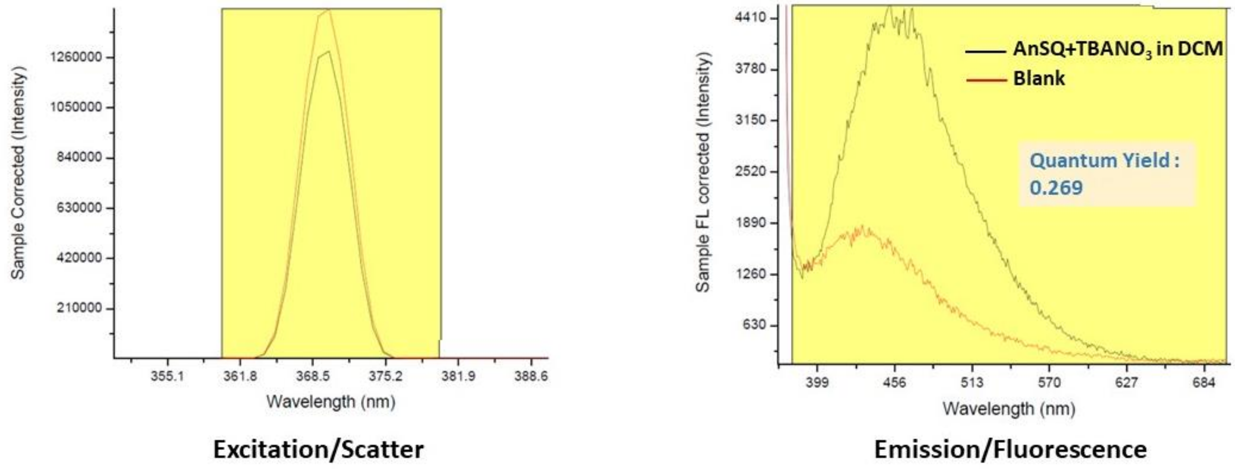


**Figure S3am.** Quantum yield of AnSQ-$NO_3^-$ in DCM

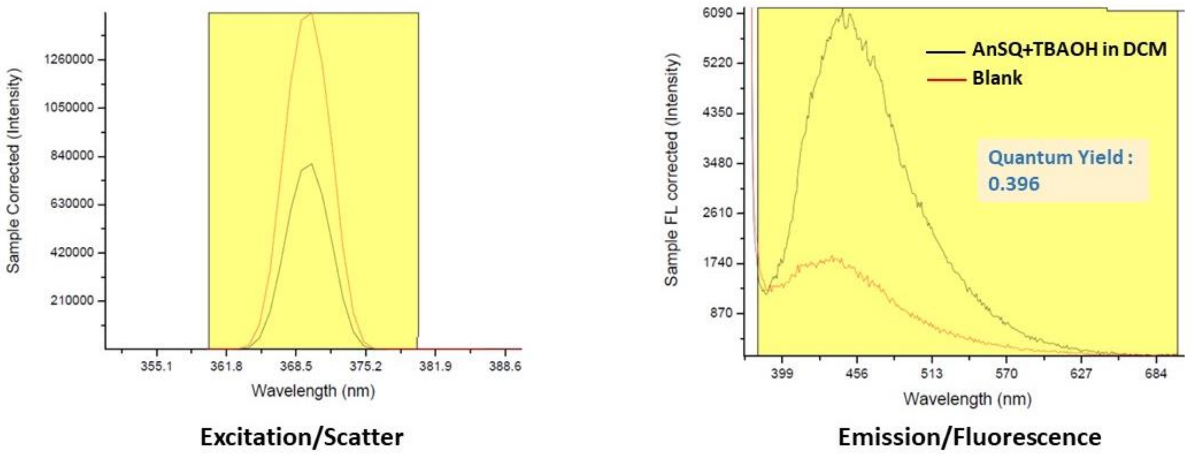


**Figure S3an.** Quantum yield of AnSQ-$OH^-$ in DCM

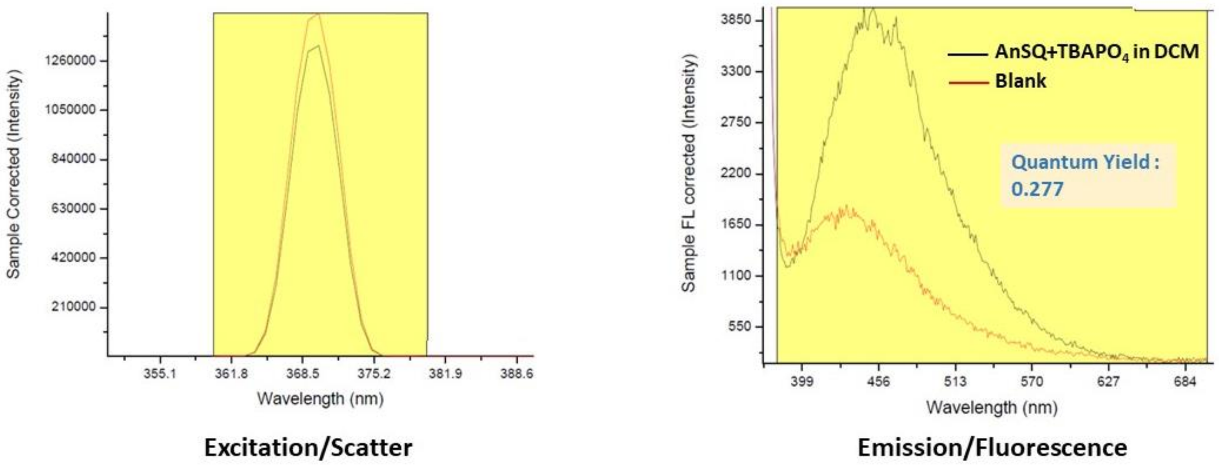


**Figure S3ao.** Quantum yield of AnSQ-$PO_4^{3-}$ in DCM

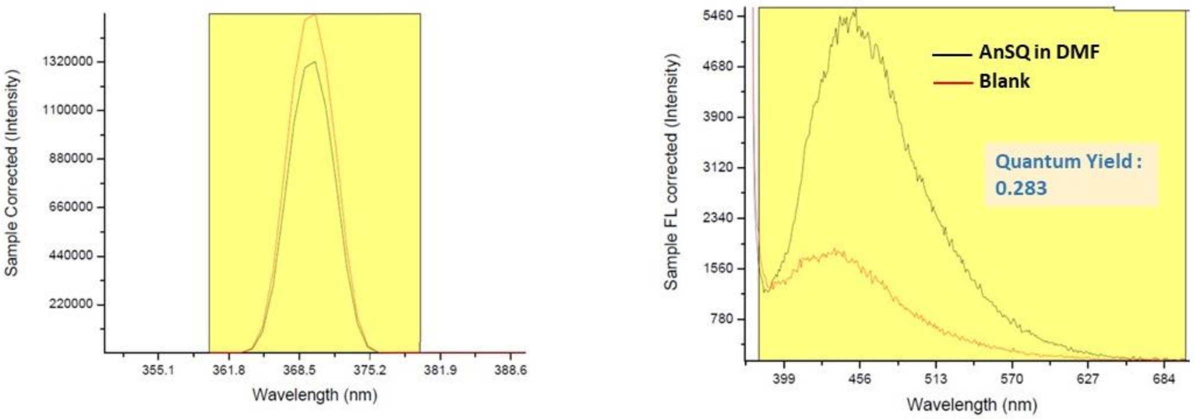


**Excitation/Scatter** **Emission/Fluorescence**

**Figure S3ap.** Quantum yield of AnSQ in DMF

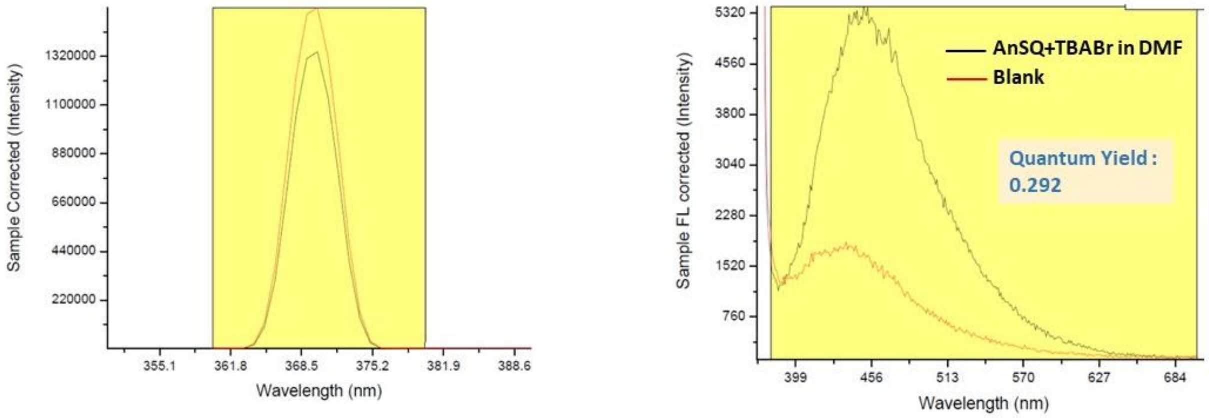


**Excitation/Scatter** **Emission/Fluorescence**

**Figure S3aq.** Quantum yield of AnSQ-$Br^-$ in DMF

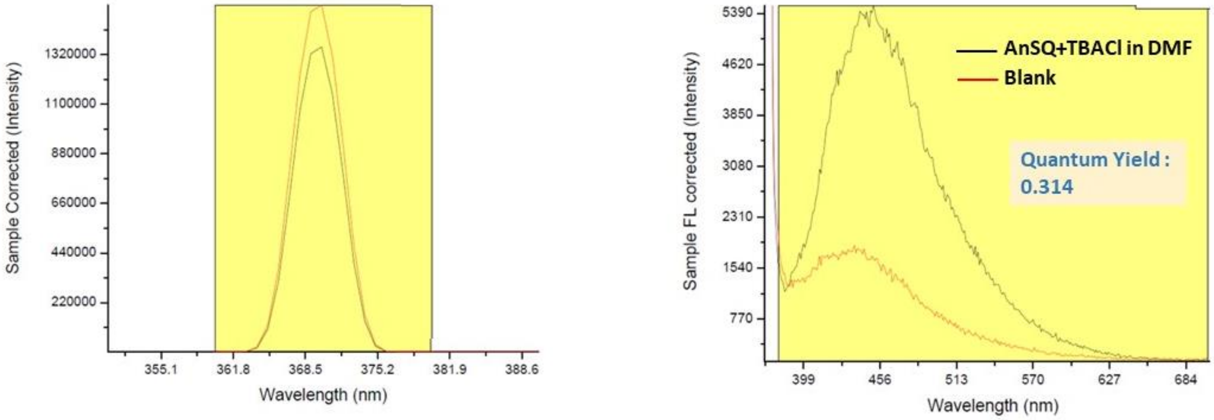


Excitation/Scatter

Emission/Fluorescence

**Figure S3ar**. Quantum yield of AnSQ-Cl$^{-}$ in DMF

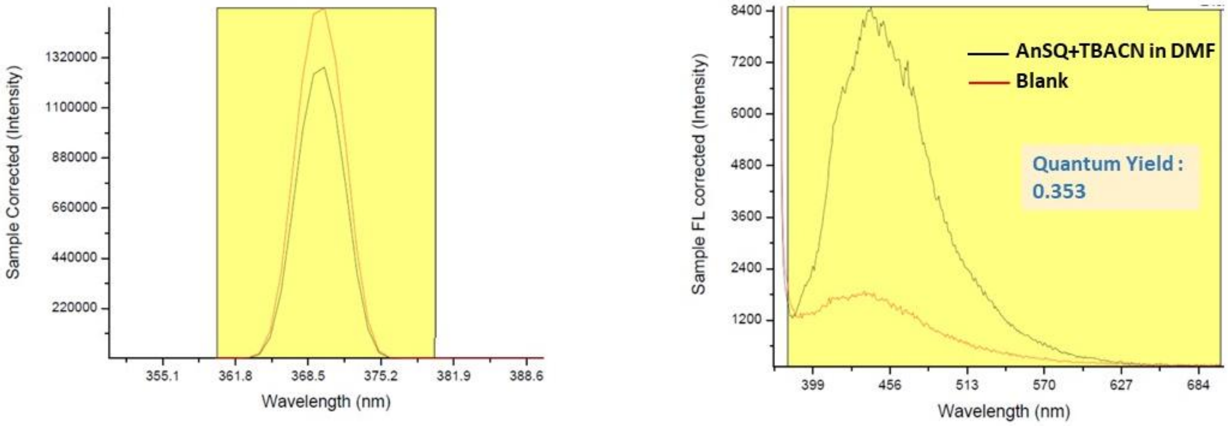


Excitation/Scatter

Emission/Fluorescence

**Figure S3as.** Quantum yield of AnSQ-CN$^{-}$ in DMF

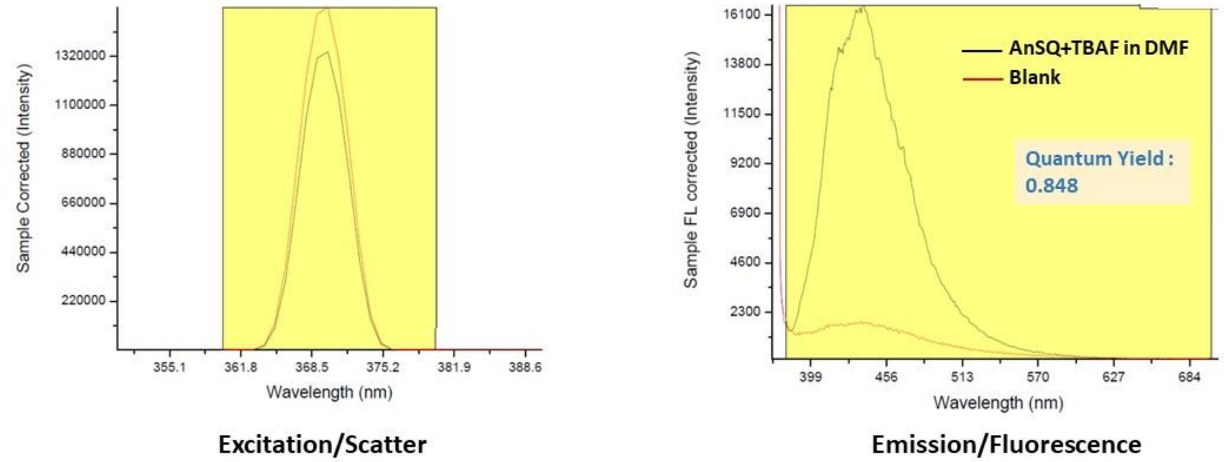


**Figure S3at.** Quantum yield of AnSQ-$F^-$ in DMF

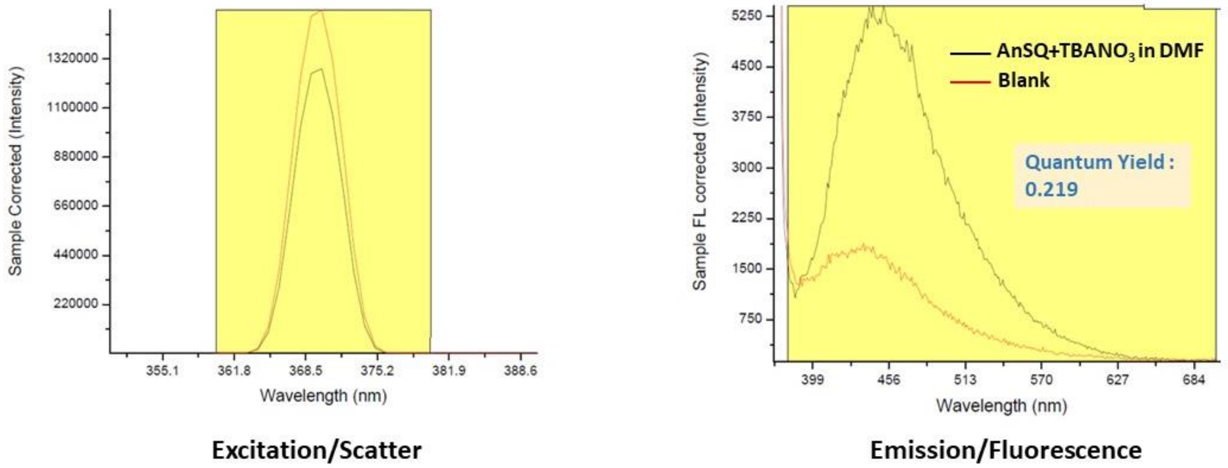


**Figure S3au.** Quantum yield of AnSQ-$NO_3^-$ in DMF

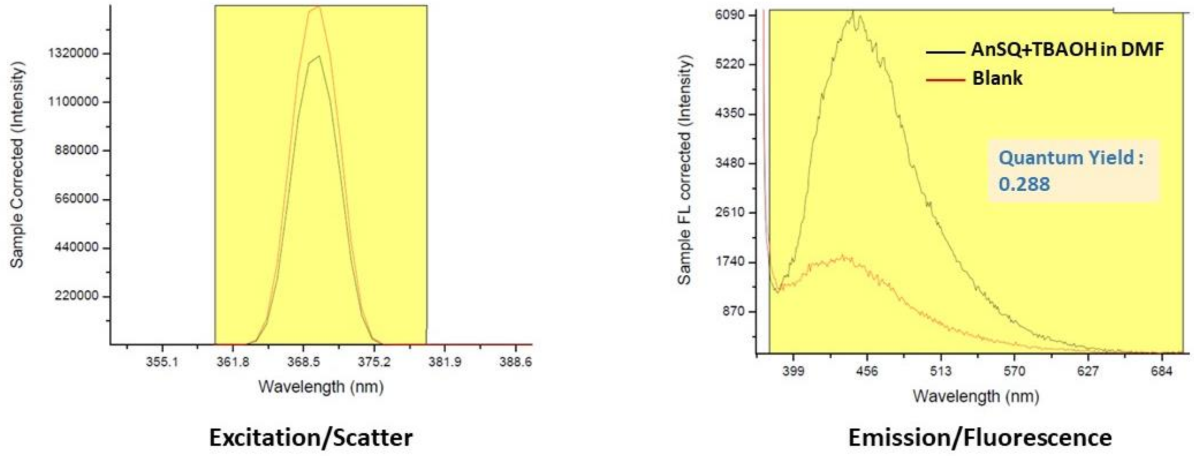


**Figure S3av.** Quantum yield of AnSQ-$OH^-$ in DMF

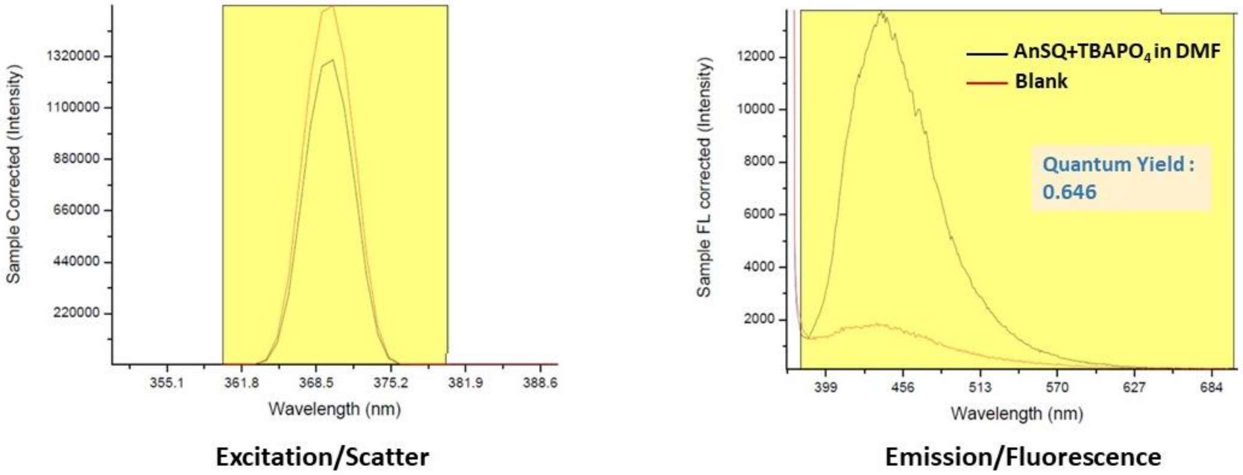


**Figure S3aw.** Quantum yield of AnSQ-$PO_4^{3-}$ in DMF

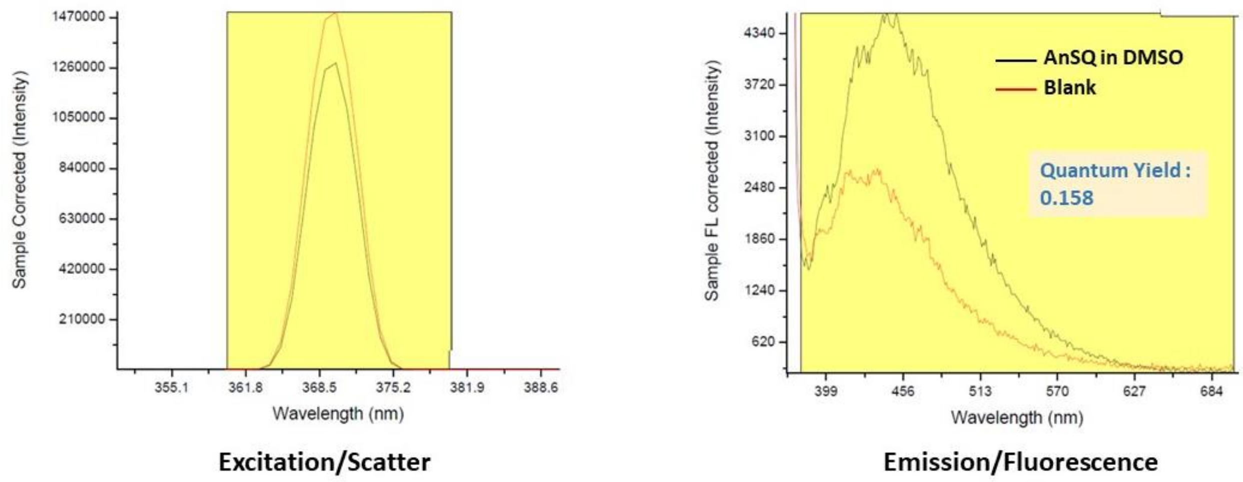


**Figure S3ax.** Quantum yield of AnSQ in DMSO

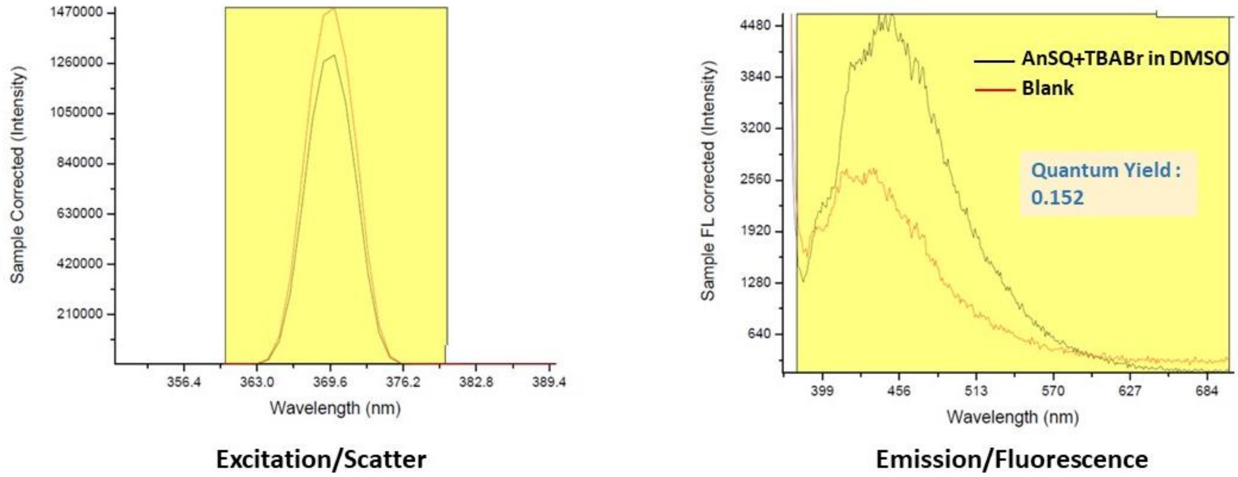


**Figure S3ay.** Quantum yield of AnSQ-$Br^-$ in DMSO

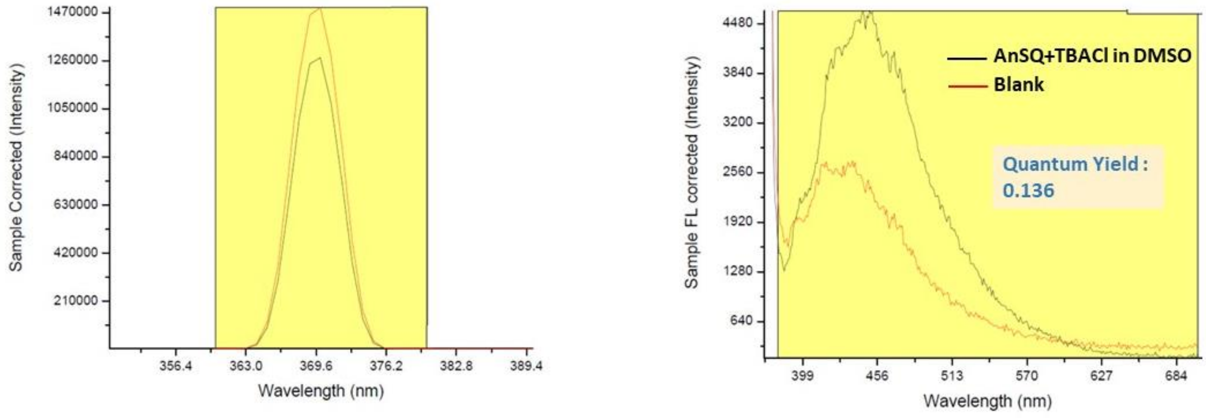


**Excitation/Scatter** **Emission/Fluorescence**

**Figure S3az.** Quantum yield of AnSQ-Cl$^{-}$ in DMSO

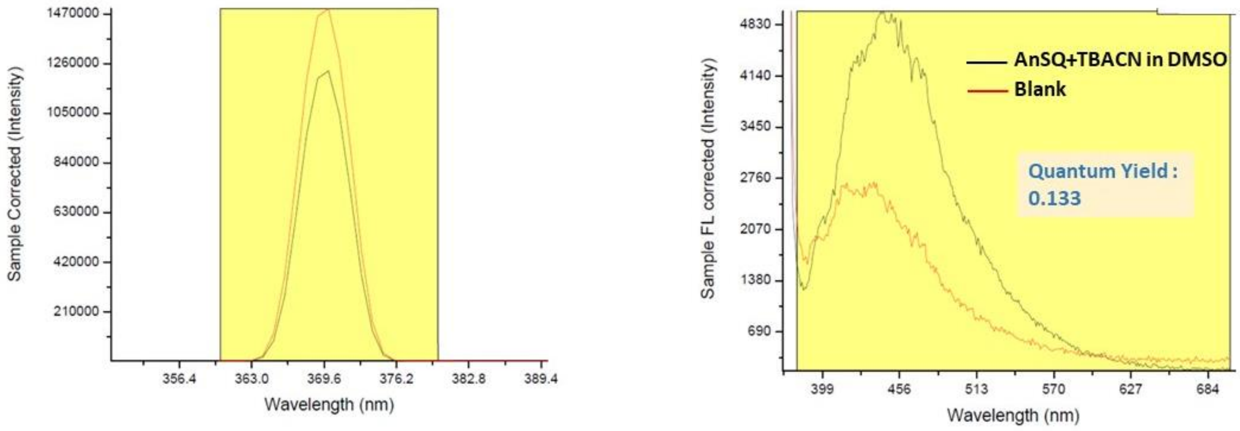


**Excitation/Scatter** **Emission/Fluorescence**

**Figure S3ba.** Quantum yield of AnSQ-CN$^{-}$ in DMSO

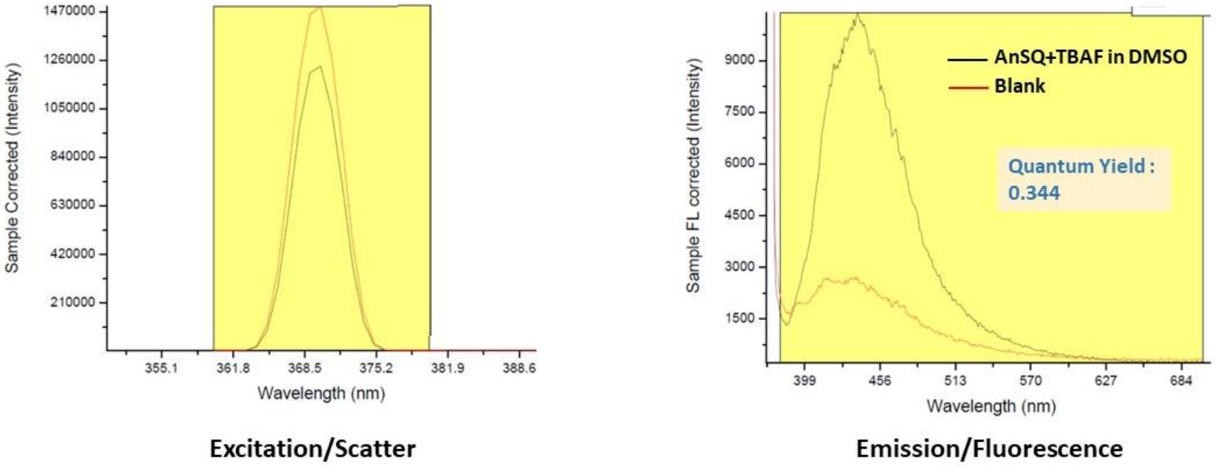


**Figure S3bb.** Quantum yield of AnSQ-$F^-$ in DMSO

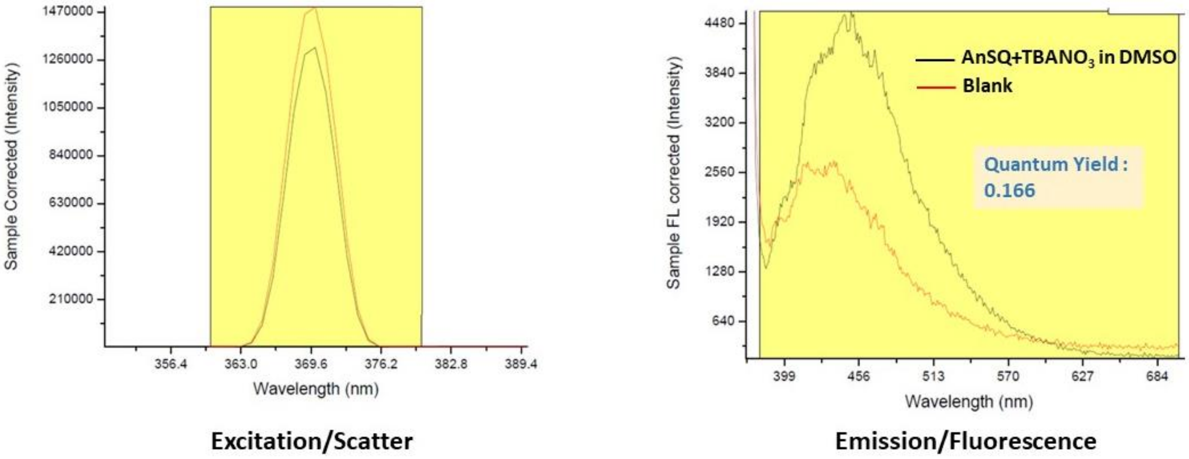


**Figure S3bc.** Quantum yield of AnSQ-$NO_3^-$ in DMSO

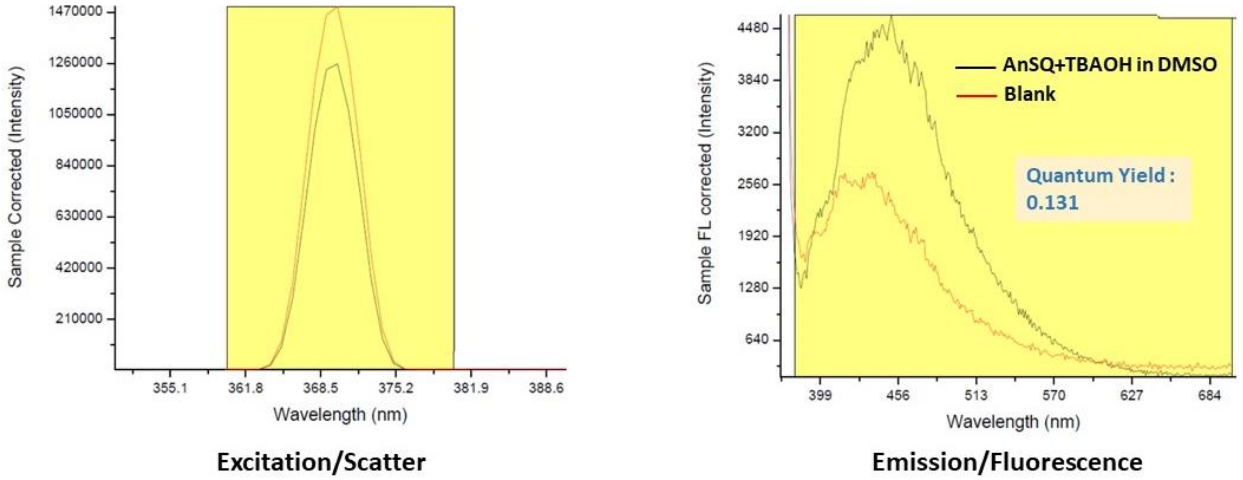


**Figure S3bd.** Quantum yield of AnSQ-$OH^-$ in DMSO

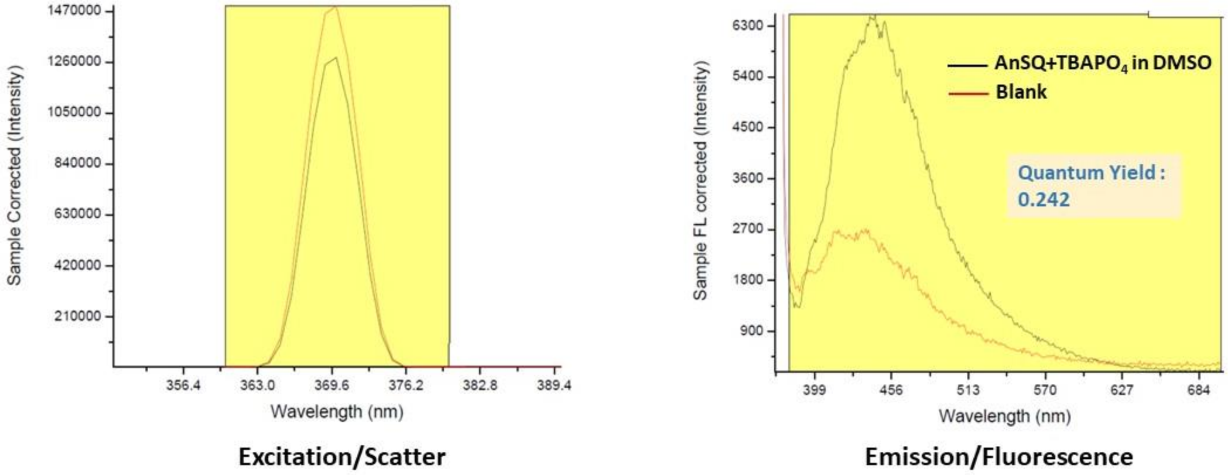


**Figure S3bf.** Quantum yield of AnSQ-$PO_4^{3-}$ in DMSO

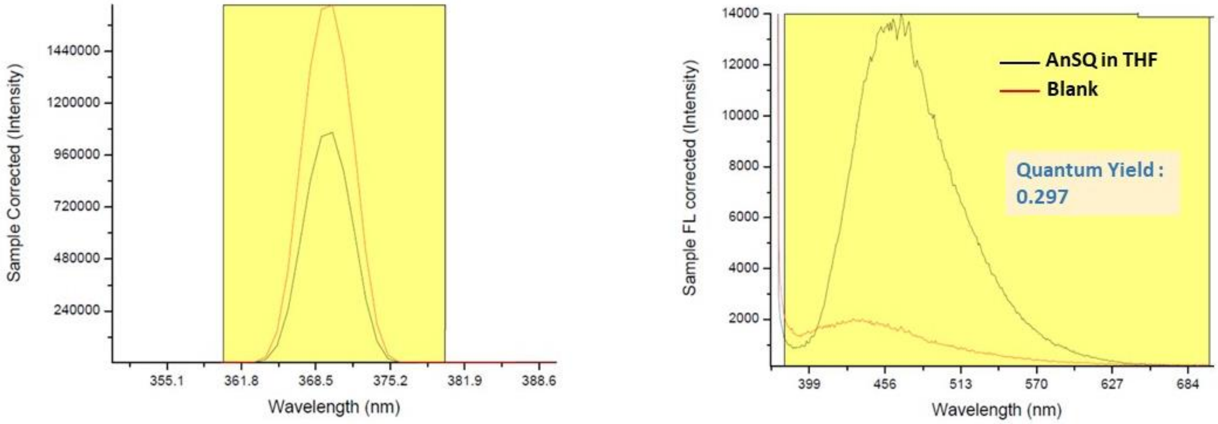


**Excitation/Scatter** **Emission/Fluorescence**

**Figure S3bg.** Quantum yield of AnSQ in THF

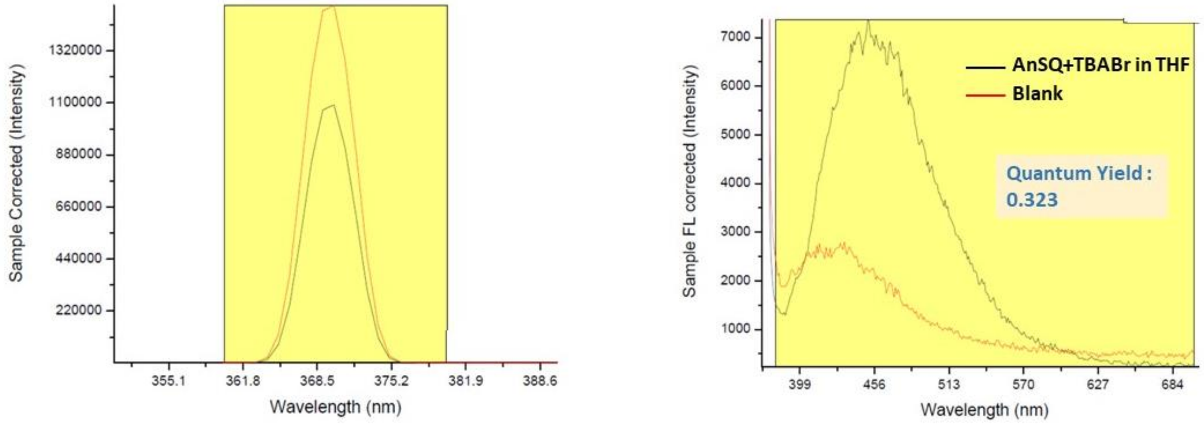


**Excitation/Scatter** **Emission/Fluorescence**

**Figure S3bh.** Quantum yield of AnSQ-$Br^-$ in THF

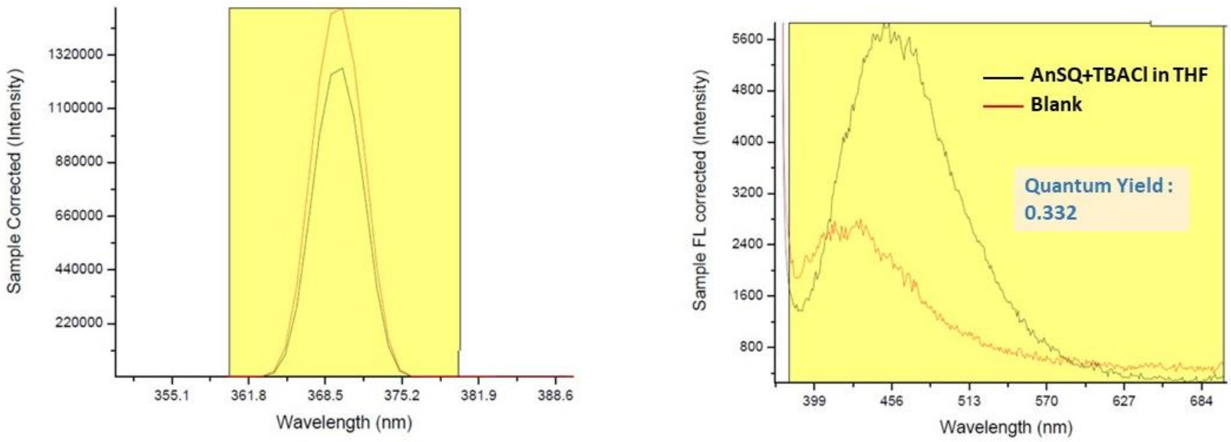


**Excitation/Scatter** **Emission/Fluorescence**

**Figure S3bi.** Quantum yield of AnSQ-$Cl^-$ in THF

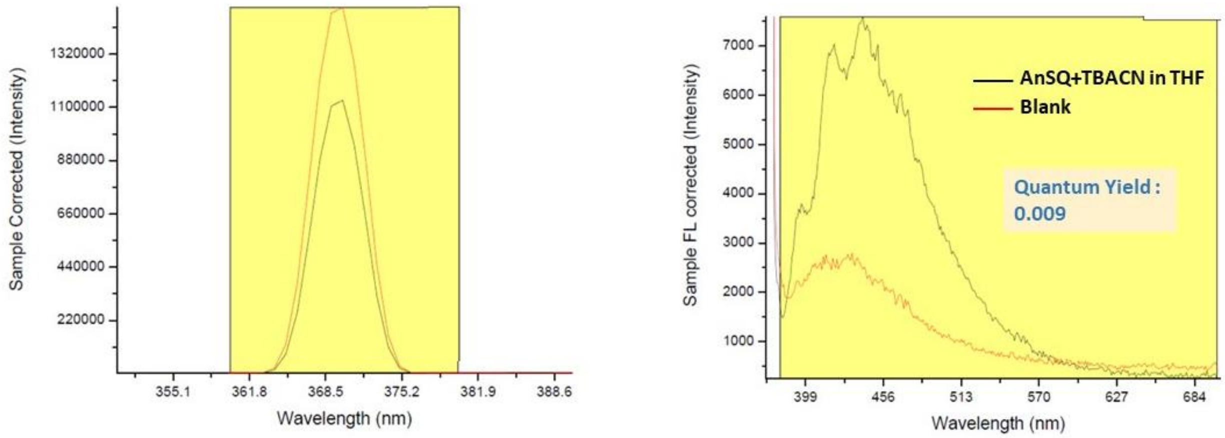


**Excitation/Scatter** **Emission/Fluorescence**

**Figure S3bj.** Quantum yield of AnSQ-$CN^-$ in THF

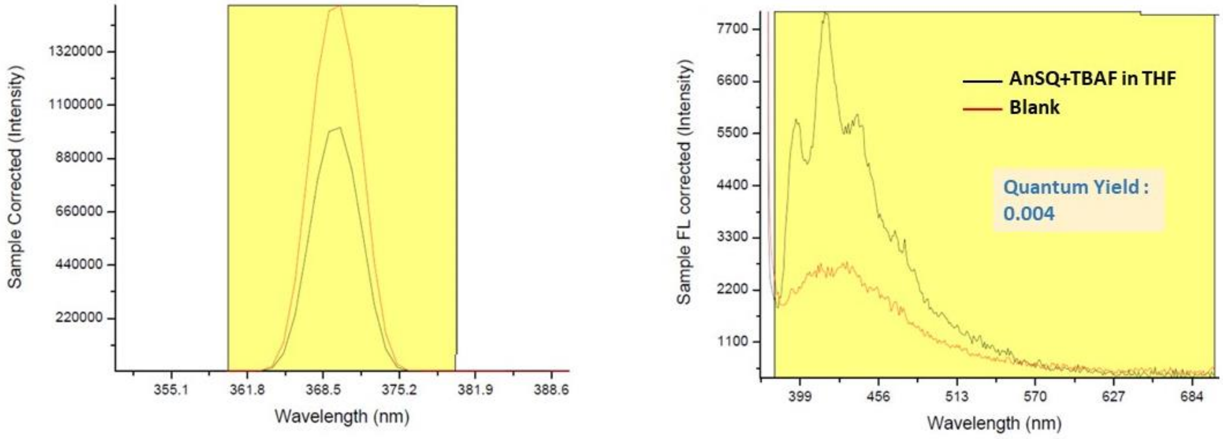


Excitation/Scatter

Emission/Fluorescence

**Figure S3bk.** Quantum yield of AnSQ-$F^-$ in THF

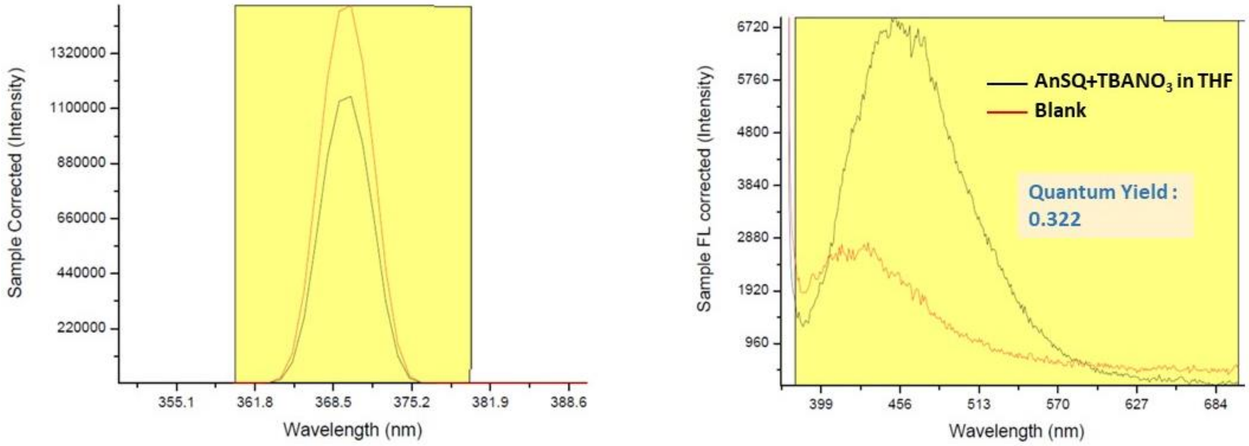


Excitation/Scatter

Emission/Fluorescence

**Figure S3bl.** Quantum yield of AnSQ-$NO_3^-$ in THF

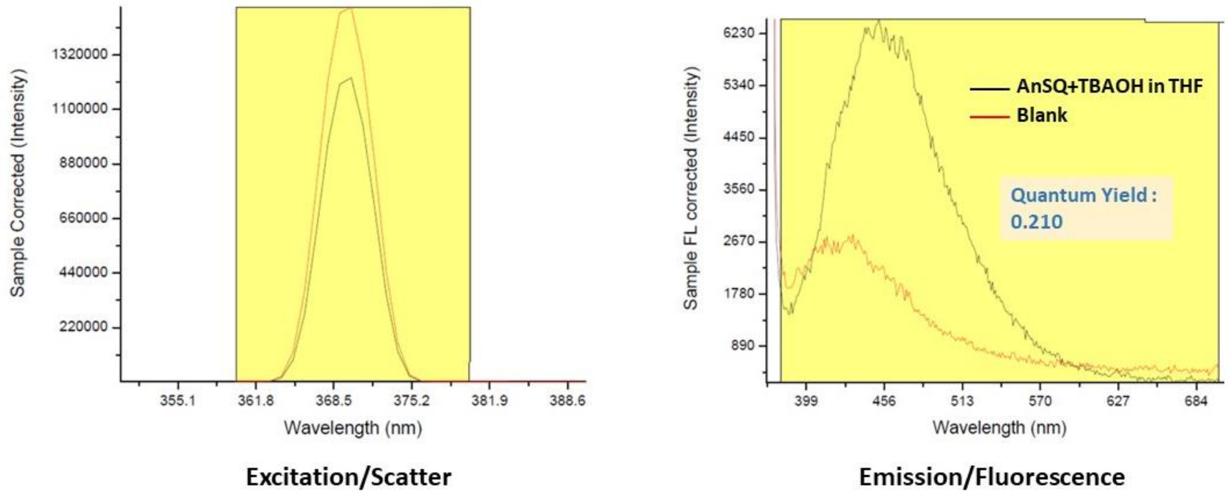


**Figure S3bm.** Quantum yield of AnSQ-$OH^-$ in THF

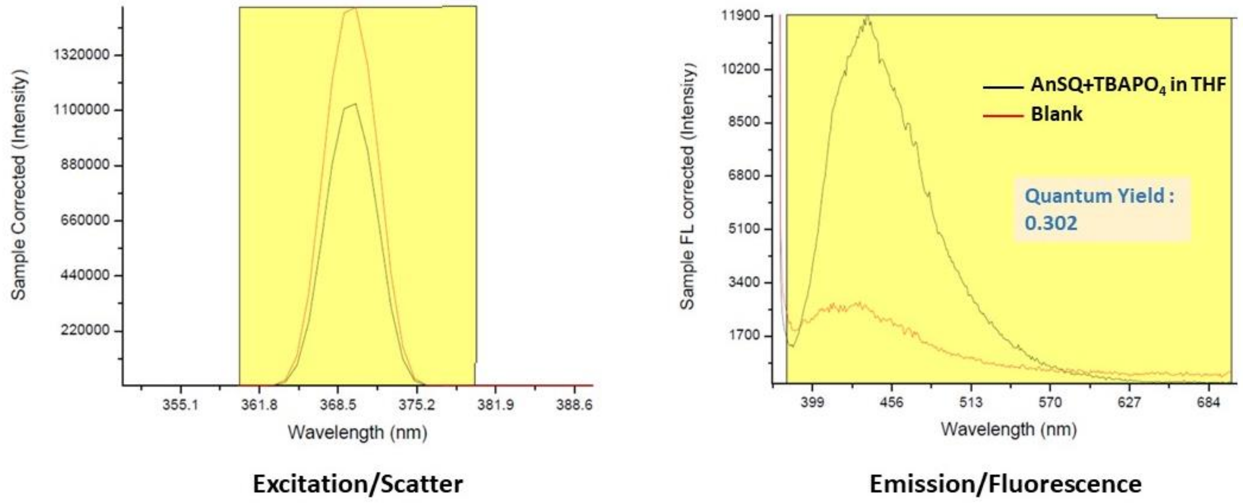


**Figure S3bn.** Quantum yield of AnSQ-$PO_4^{3-}$ in THF

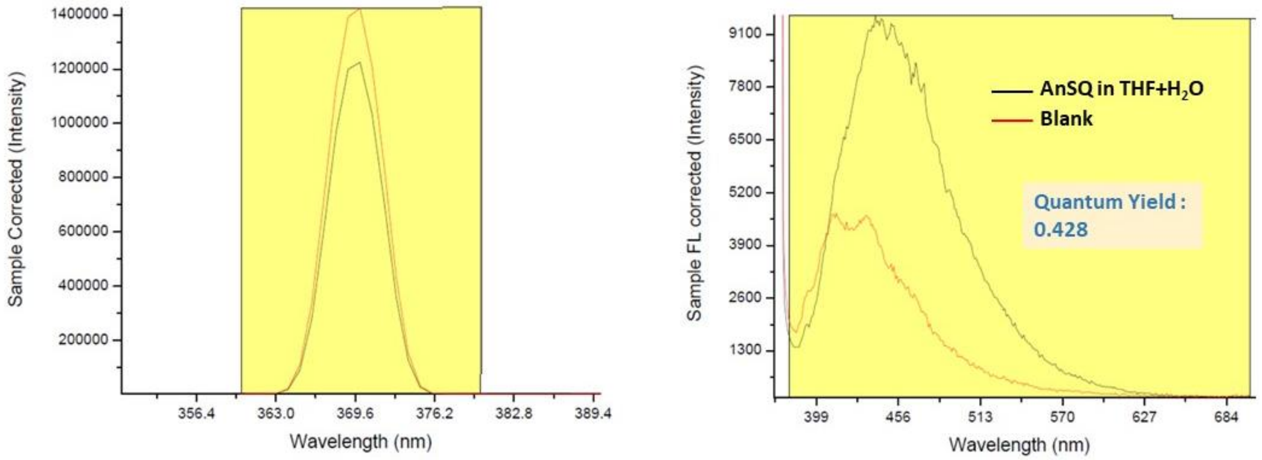


**Excitation/Scatter** **Emission/Fluorescence**

**Figure S3bo.** Quantum yield of AnSQ in THF+$H_2O$

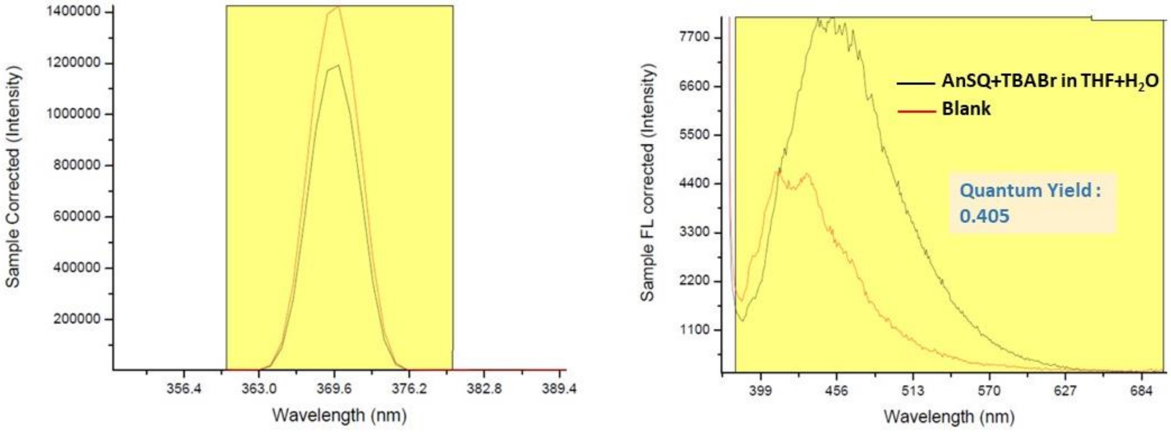


**Excitation/Scatter** **Emission/Fluorescence**

**Figure S3bp.** Quantum yield of AnSQ-$Br^-$ in THF+$H_2O$

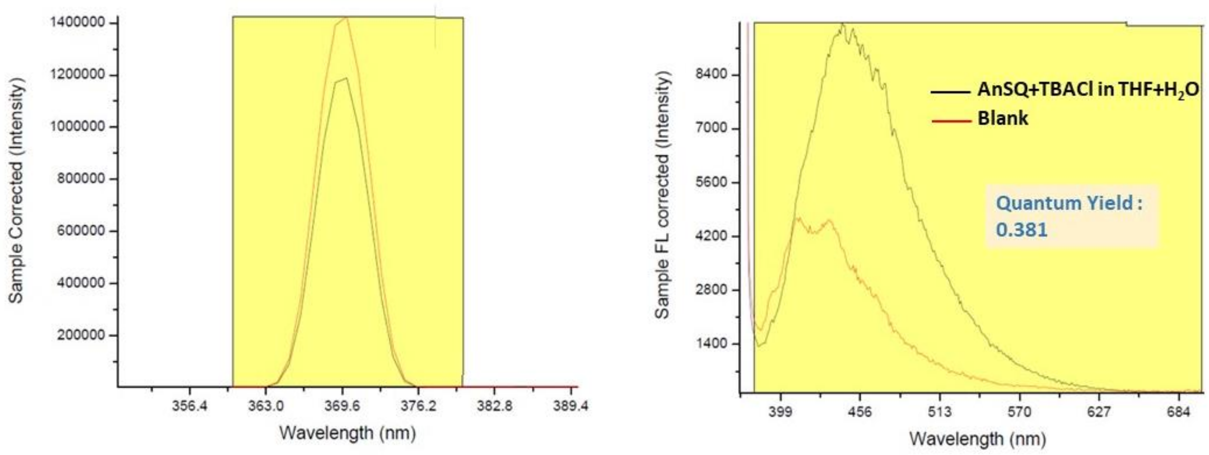


**Excitation/Scatter** **Emission/Fluorescence**

**Figure S3bq.** Quantum yield of AnSQ-$Cl^-$ in THF+$H_2O$

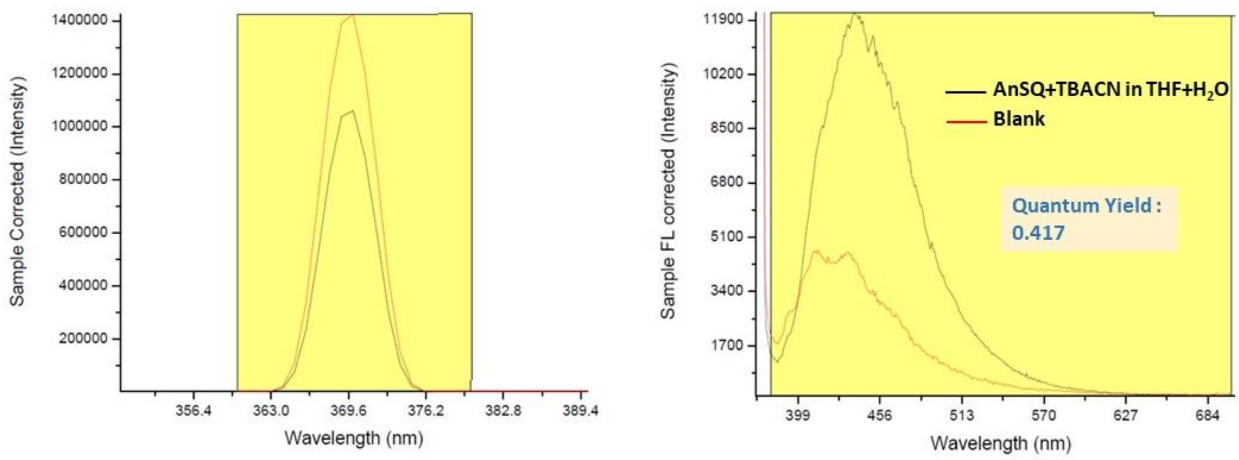


**Excitation/Scatter** **Emission/Fluorescence**

**Figure S3br.** Quantum yield of AnSQ-$CN^-$ in THF+$H_2O$

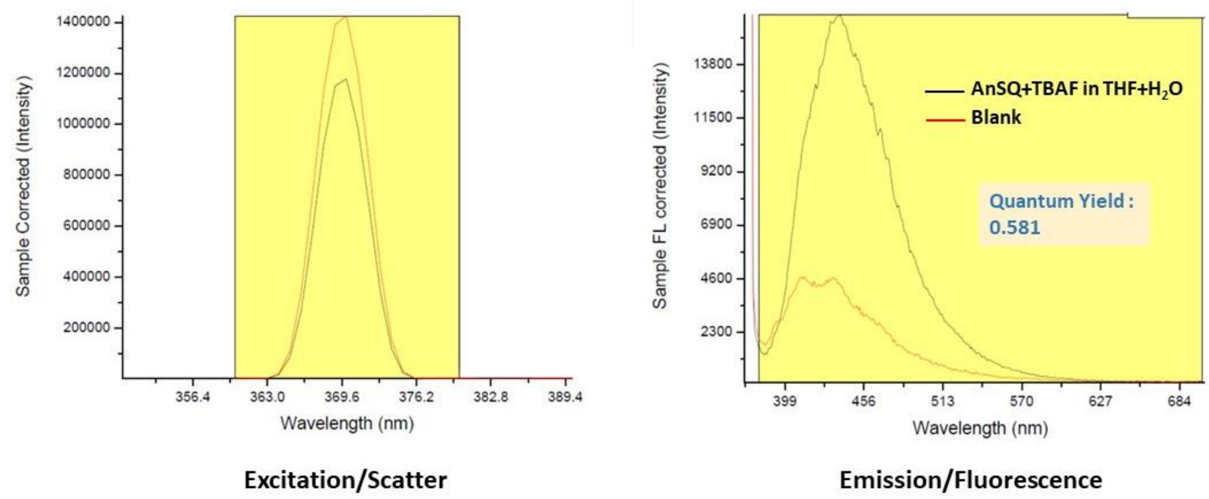


**Figure S3bs.** Quantum yield of AnSQ-$F^-$ in THF+$H_2O$

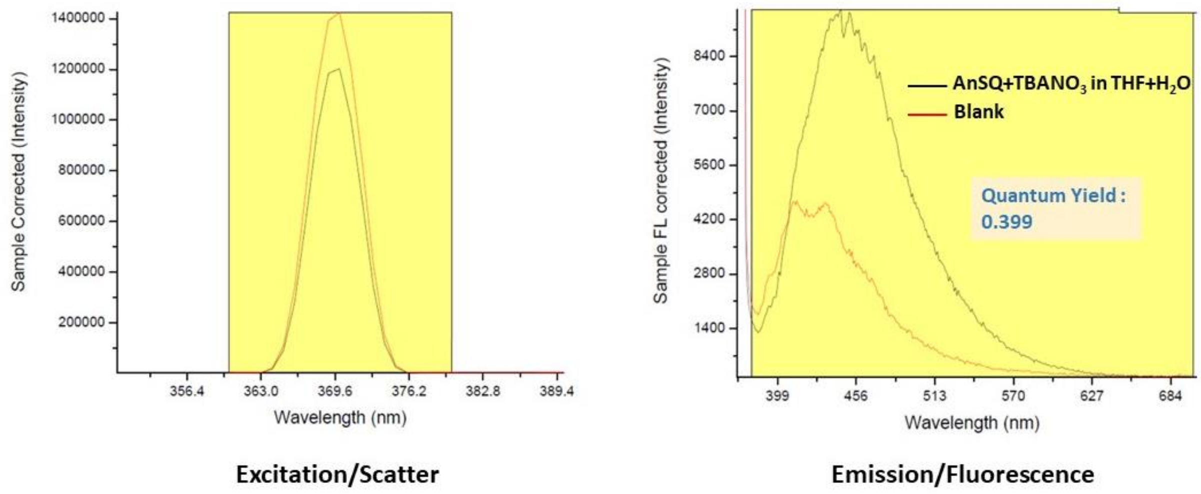


**Figure S3bt.** Quantum yield of AnSQ-$NO_3^-$ in THF+$H_2O$

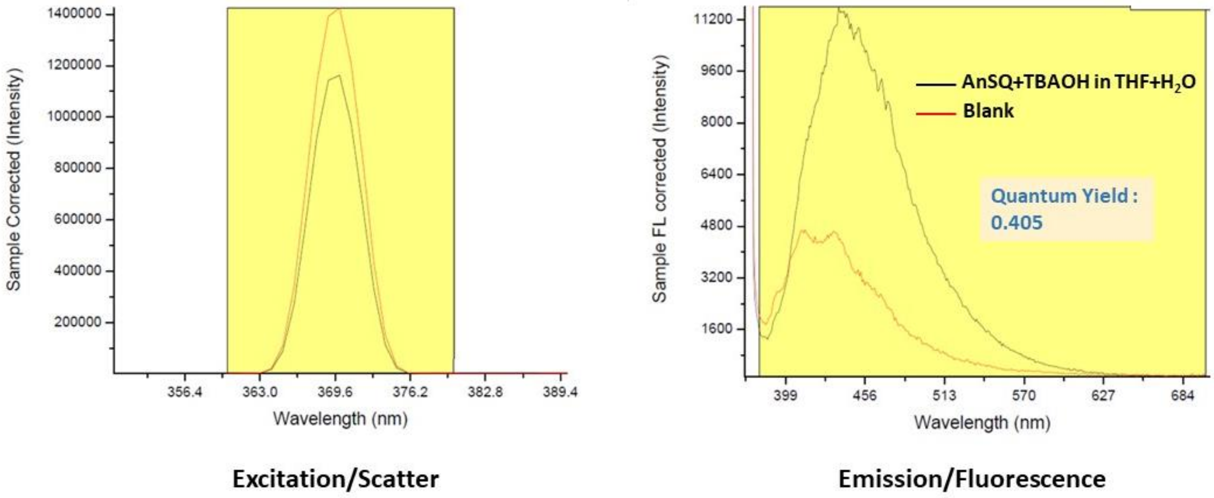


**Figure S3bu.** Quantum yield of AnSQ-$OH^-$ in THF+$H_2O$

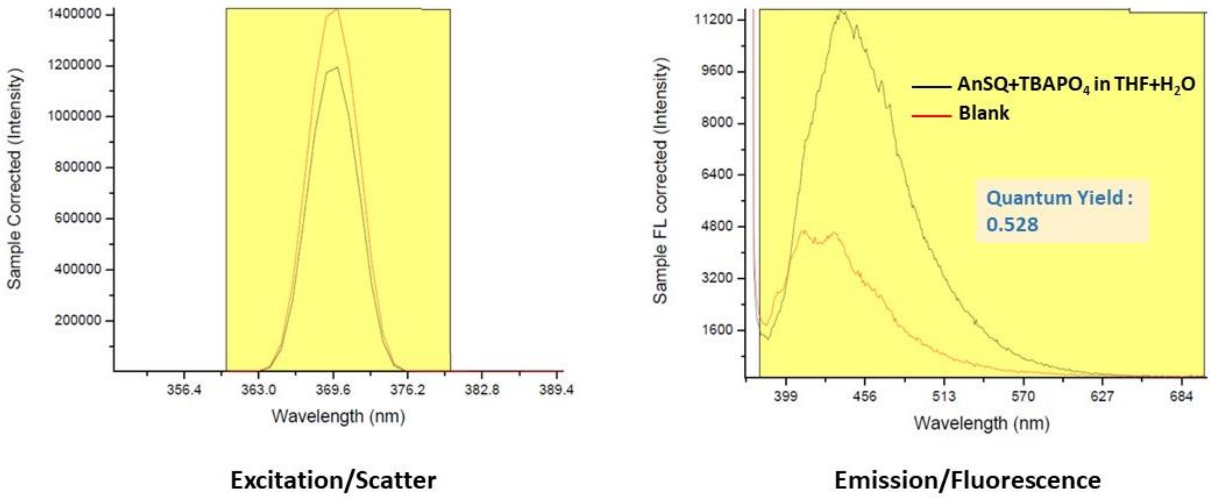


**Figure S3bv.** Quantum yield of AnSQ-$PO_4^{3-}$ in THF+$H_2O$

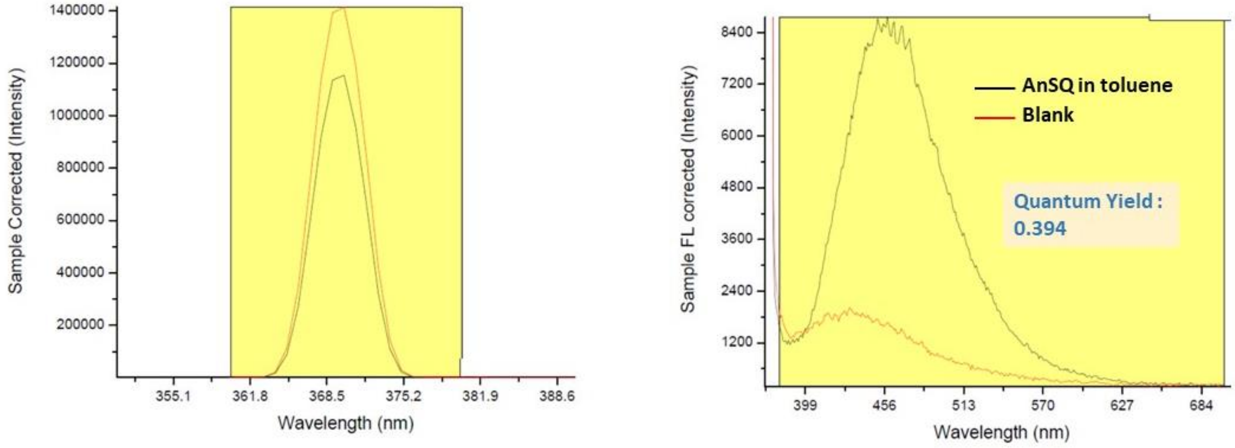


**Excitation/Scatter** **Emission/Fluorescence**

**Figure S3bw.** Quantum yield of AnSQ in toluene

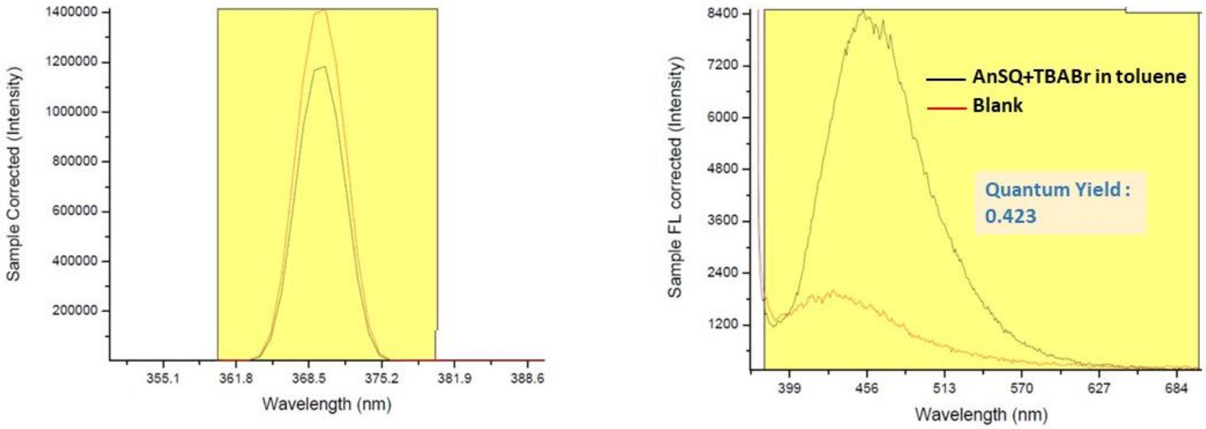


**Excitation/Scatter** **Emission/Fluorescence**

**Figure S3bx.** Quantum yield of AnSQ-$Br^{-}$ in toluene

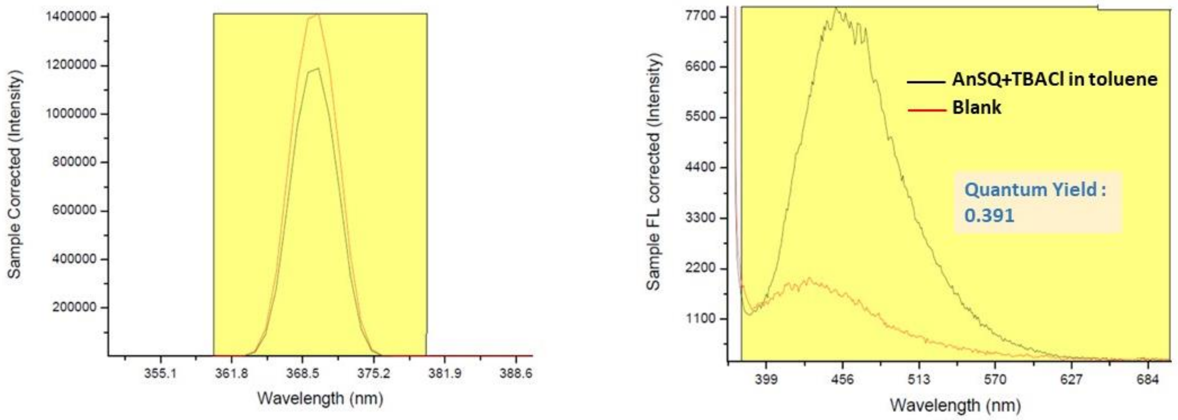


**Excitation/Scatter** **Emission/Fluorescence**

**Figure S3by.** Quantum yield of AnSQ-Cl$^-$ in toluene

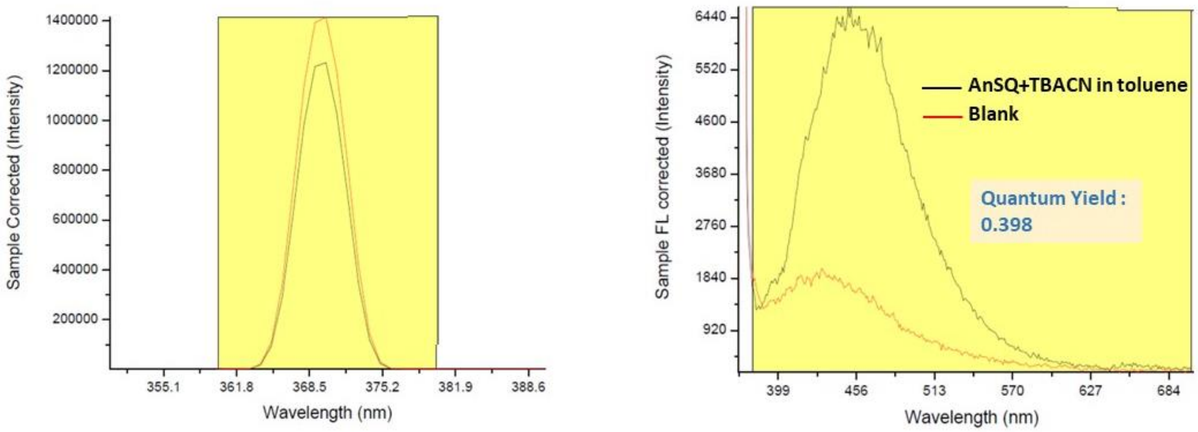


**Excitation/Scatter** **Emission/Fluorescence**

**Figure S3bz.** Quantum yield of AnSQ-CN$^-$ in toluene

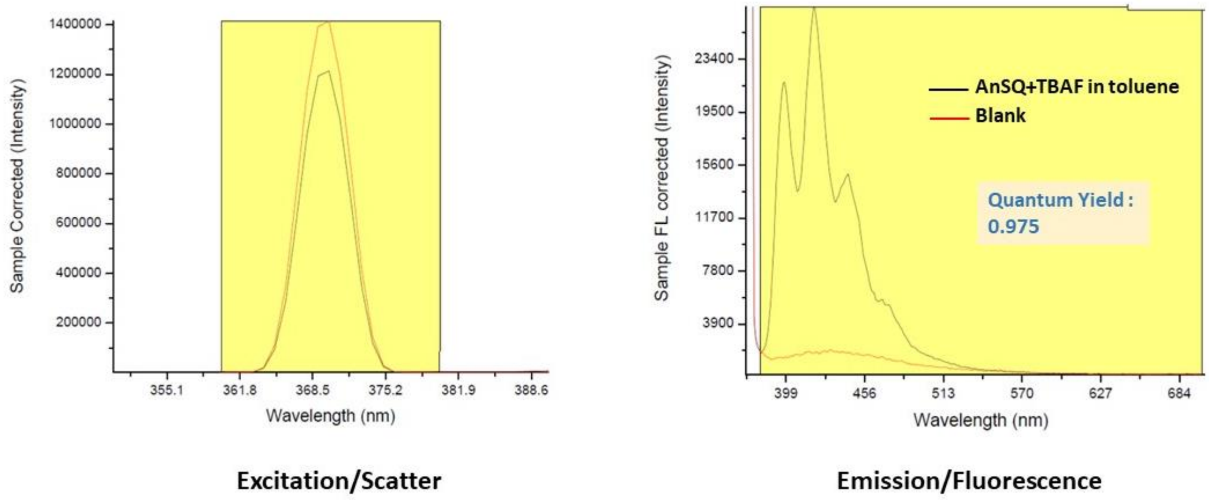


**Figure S3ca.** Quantum yield of AnSQ-$F^-$ in toluene

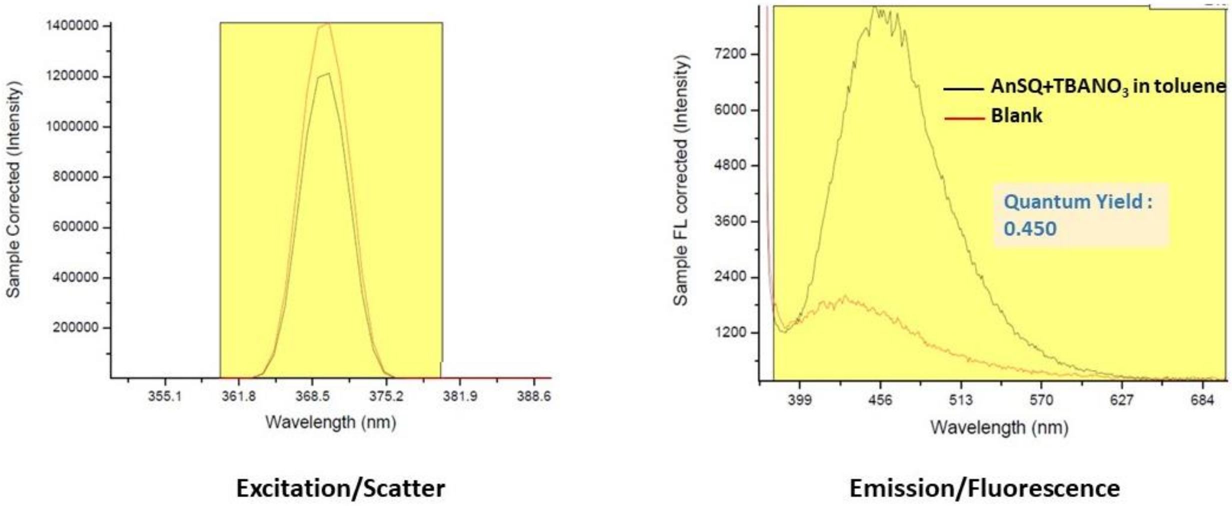


**Figure S3cb.** Quantum yield of AnSQ-$NO_3^-$ in toluene

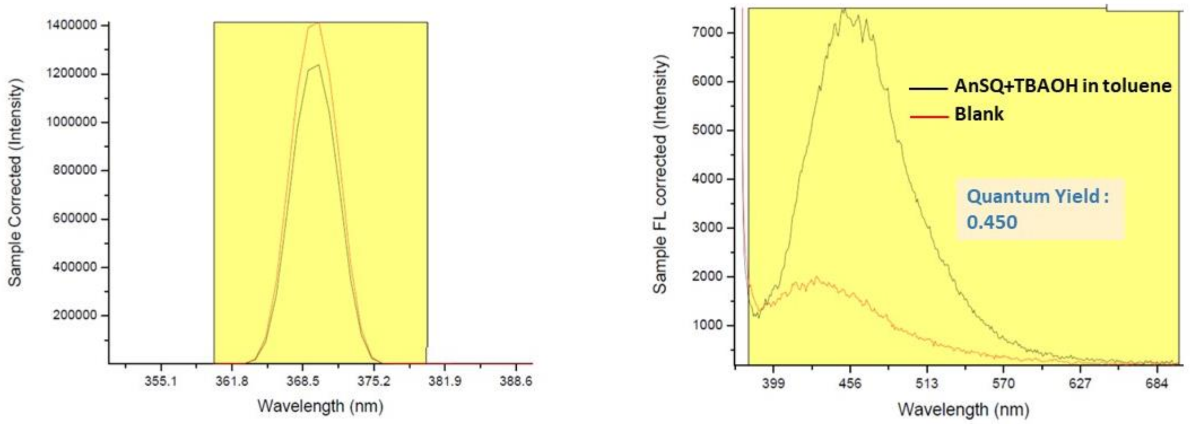


**Excitation/Scatter** **Emission/Fluorescence**

**Figure S3cc.** Quantum yield of AnSQ-$OH^-$ in toluene

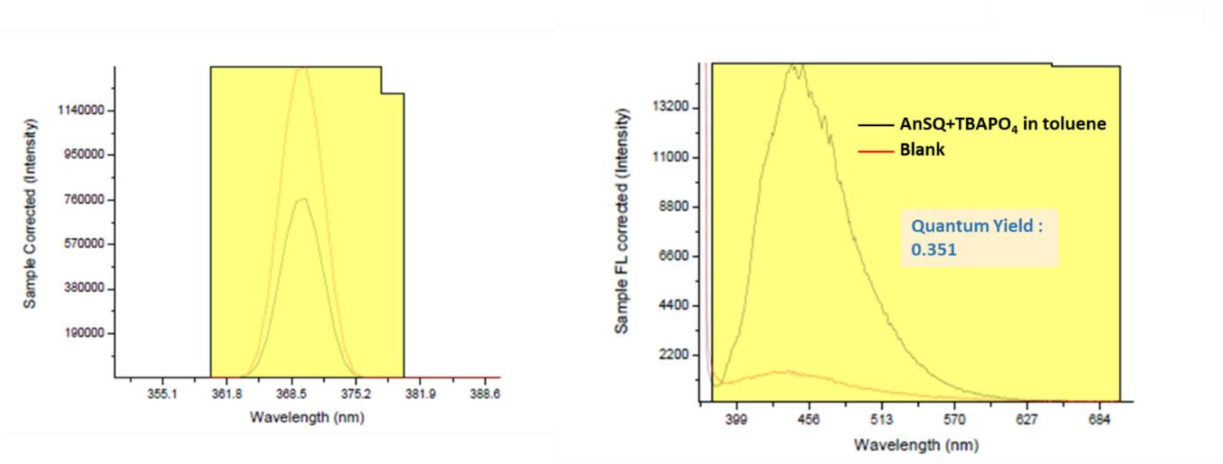


**Excitation/Scatter** **Emission/Fluorescence**

**Figure S3cd.** Quantum yield of AnSQ-$PO_4^{3-}$ in toluene

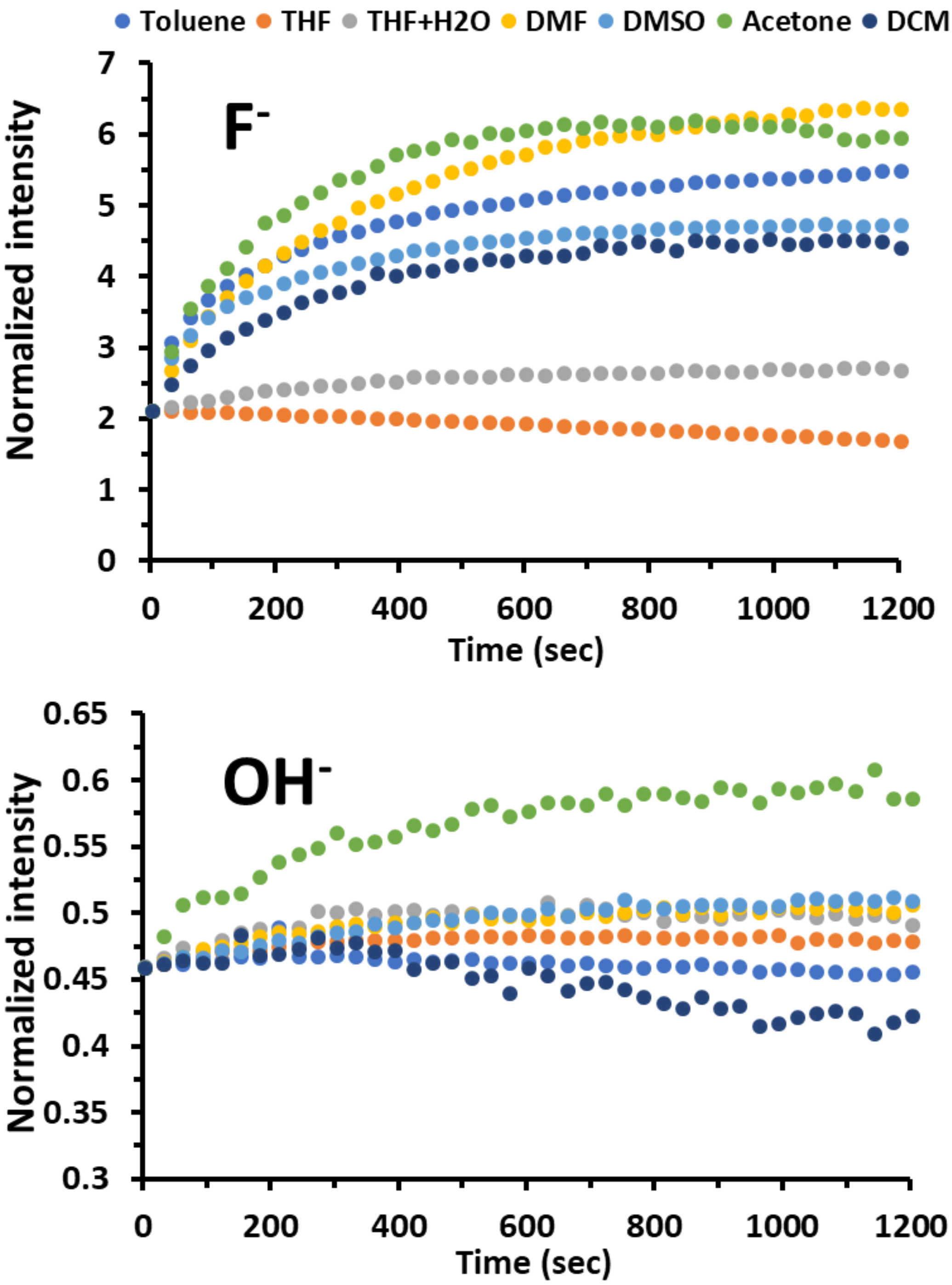


**Figure S4a.** Kinetic studies for anions addition to AnSQ in term of fluorescent intensity (top) fluoride ion (bottom) hydroxide ion with $\lambda_{ex}$ = 370 nm and $\lambda_{em}$ were fixed in accordance with table 1

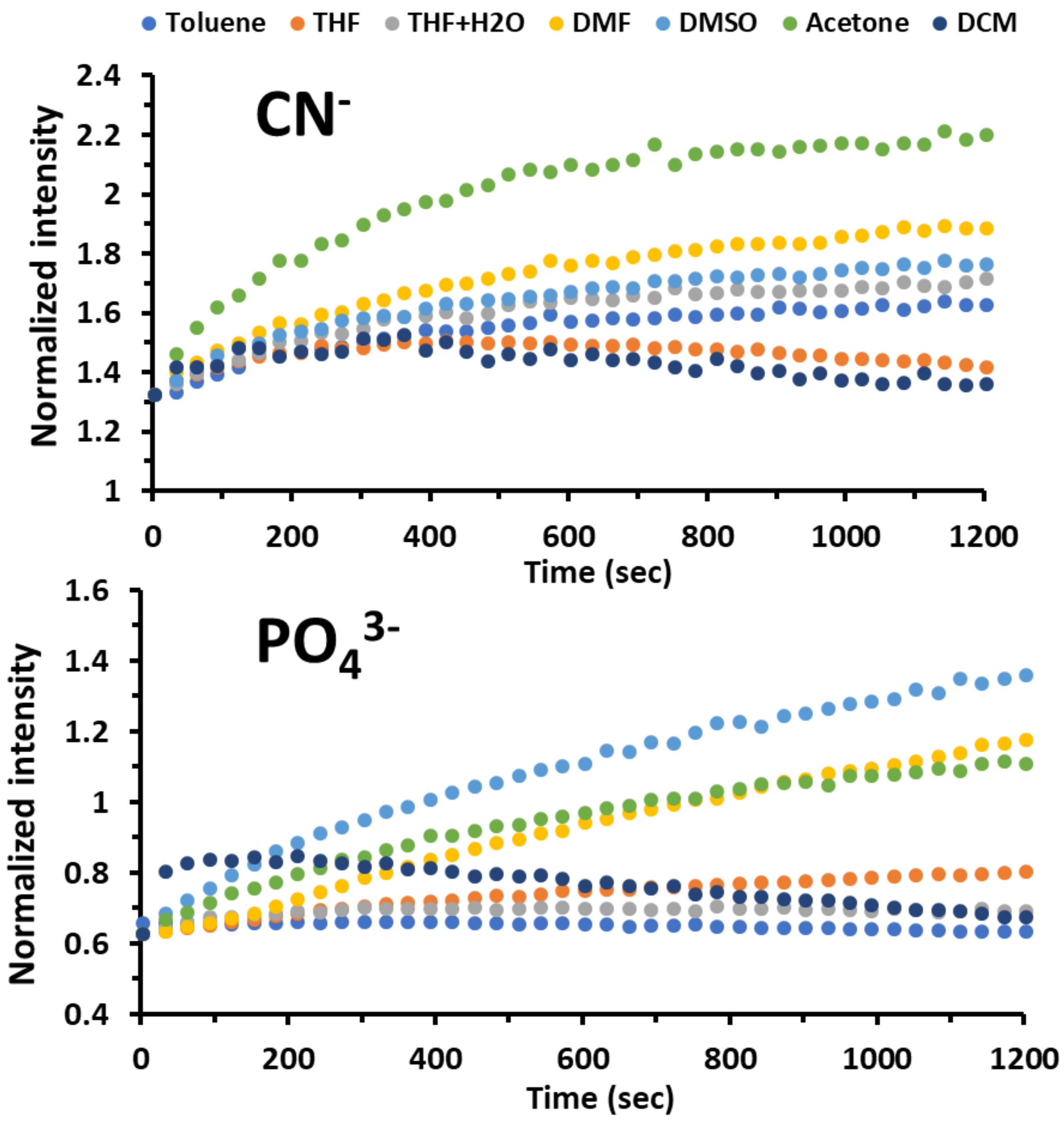


**Figure S4b.** Kinetic studies for anions addition to AnSQ in term of fluorescent intensity (top) cyanide ion (bottom) phosphate ion with $\lambda_{ex}$ = 370 nm and $\lambda_{em}$ were fixed in accordance with table 1

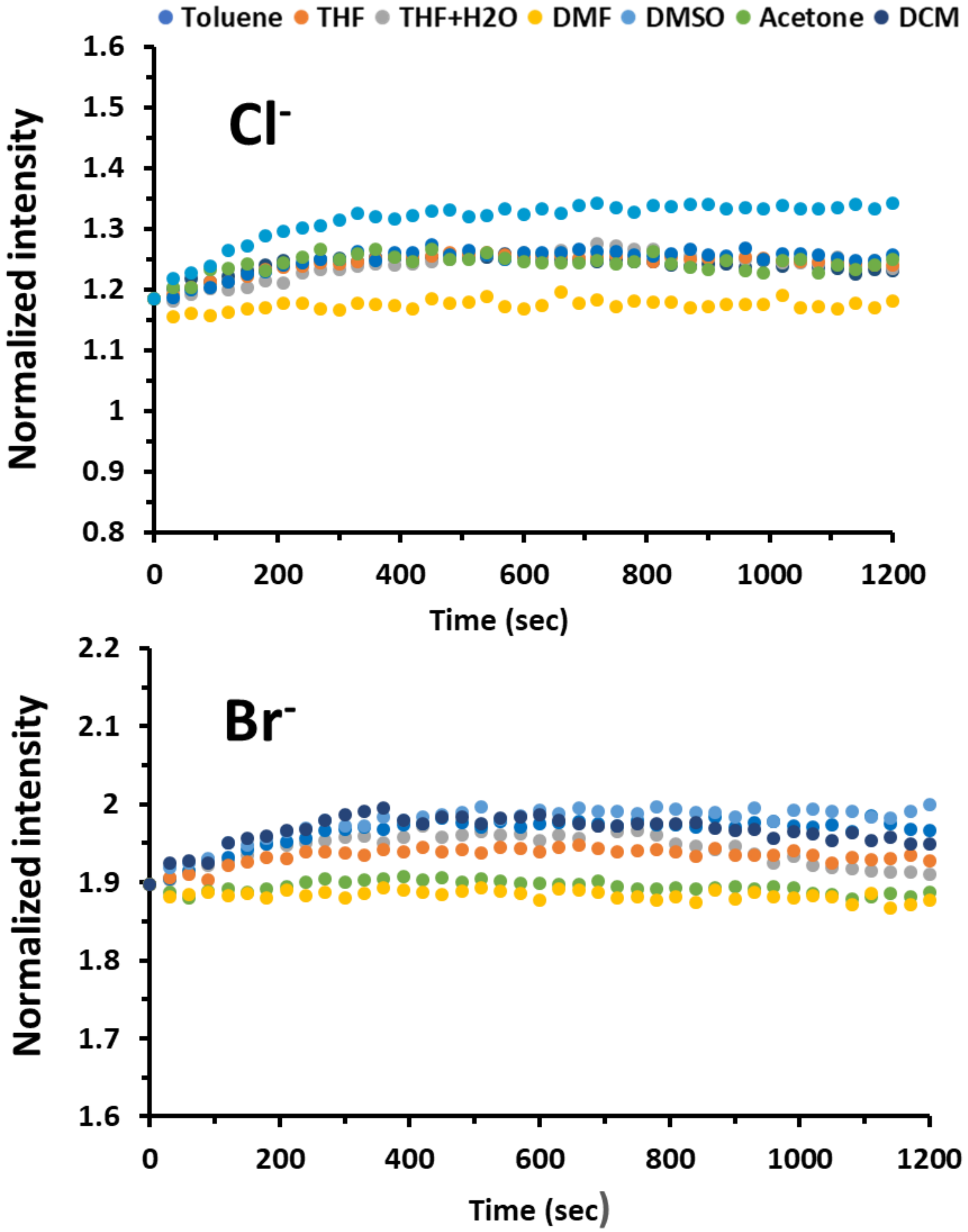


**Figure S4c.** Kinetic studies for anions addition to AnSQ in term of fluorescent intensity (top) chloride ion (bottom) bromide ion with $\lambda_{ex}$ = 370 nm and $\lambda_{em}$ were fixed in accordance with table 1

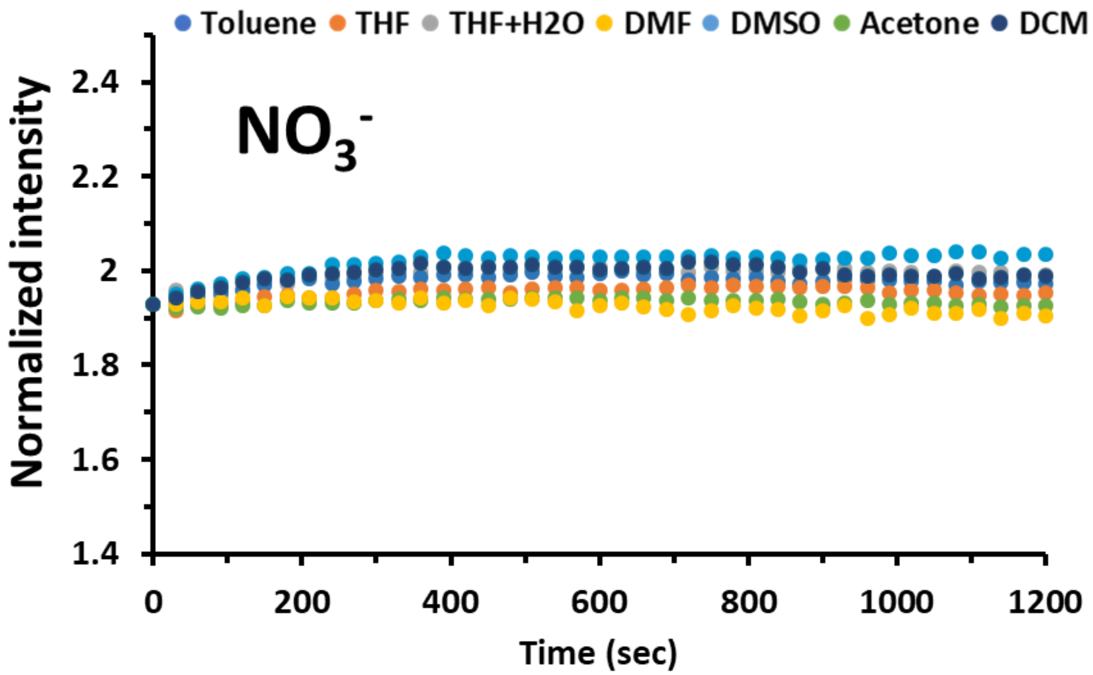


**Figure S4d.** Kinetic studies for anions addition to AnSQ in term of fluorescent intensity nitrate ion with $\lambda_{ex}$ = 370 nm and $\lambda_{em}$ were fixed in accordance with table 1

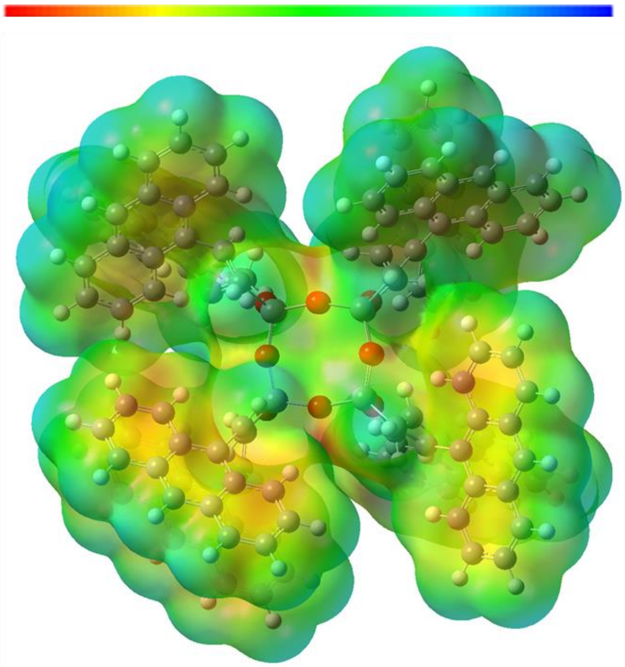

**Figure S5a.** Electrostatic potential surface mapping (EPS) of $T_8R_8$ at 8 substitutions of 9-vinylanthracene. EPS was generated by gaussian03 under the DFT calculation with basis set Lan2MB including B3LYP.

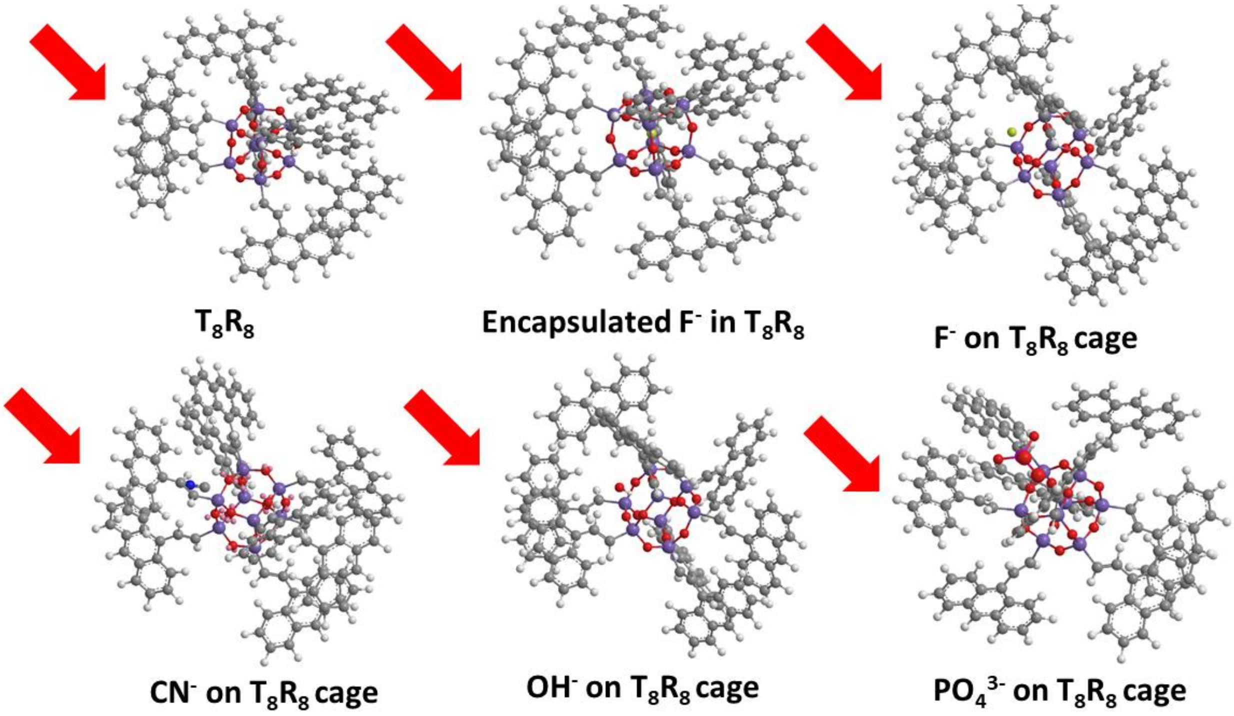


**Figure S5b.** MM2 minimalized with no solvation of $T_8R_8$ showing the different excimer formations after anions addition.

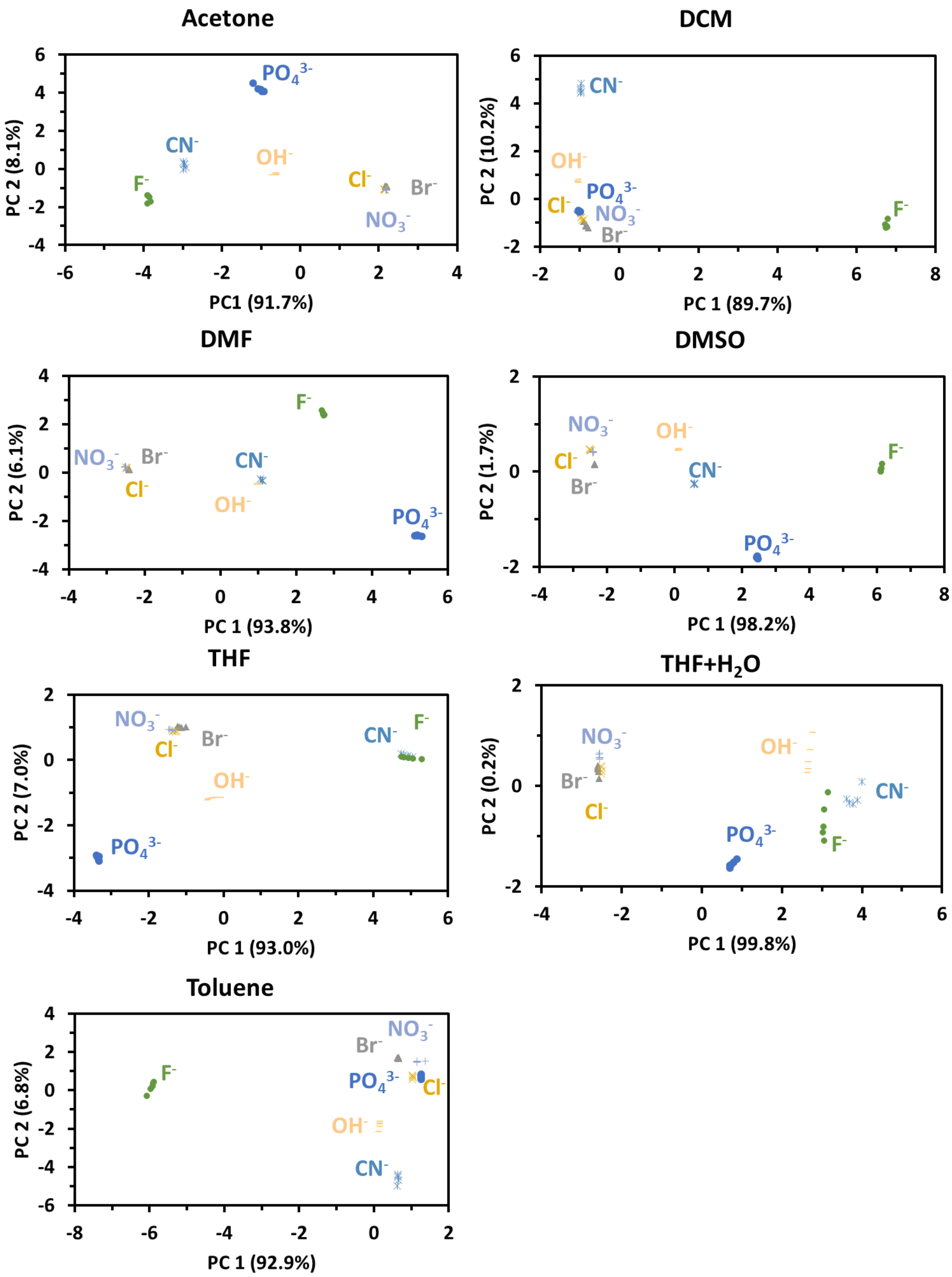


**Figure S6.** Principle component analysis results of AnSQ after 100 eq addition of anions, by collecting fluorescent intensity, anion and solvent as raw data.

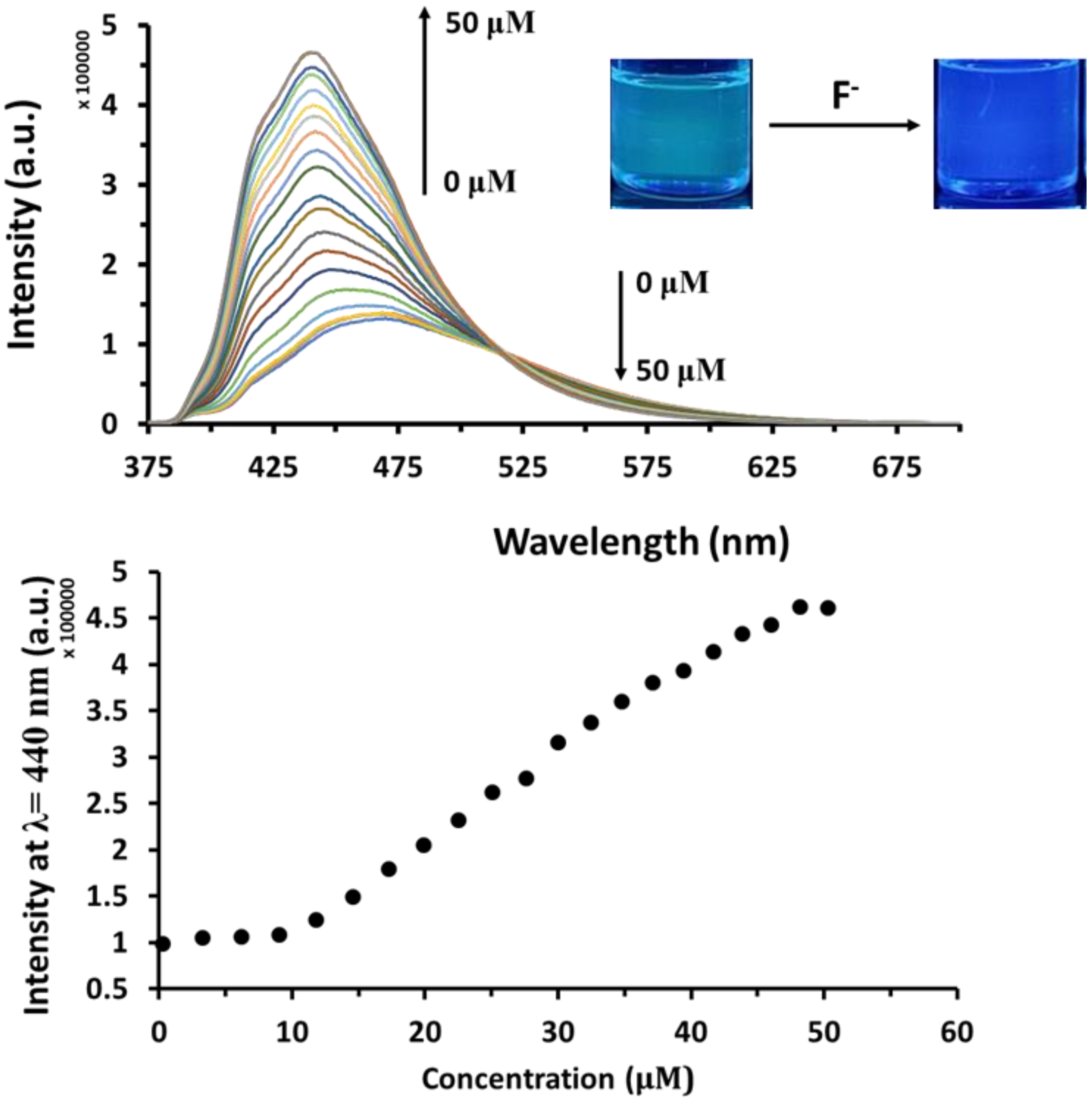


**Figure S7a.** (top) Fluorescent titration result at $\lambda_{ex}$= 370 nm of 6 µM of AnSQ upon the addition of fluoride ion in DMSO as solvent (bottom) intensity at $\lambda_{em}$ = 440 nm in DMSO

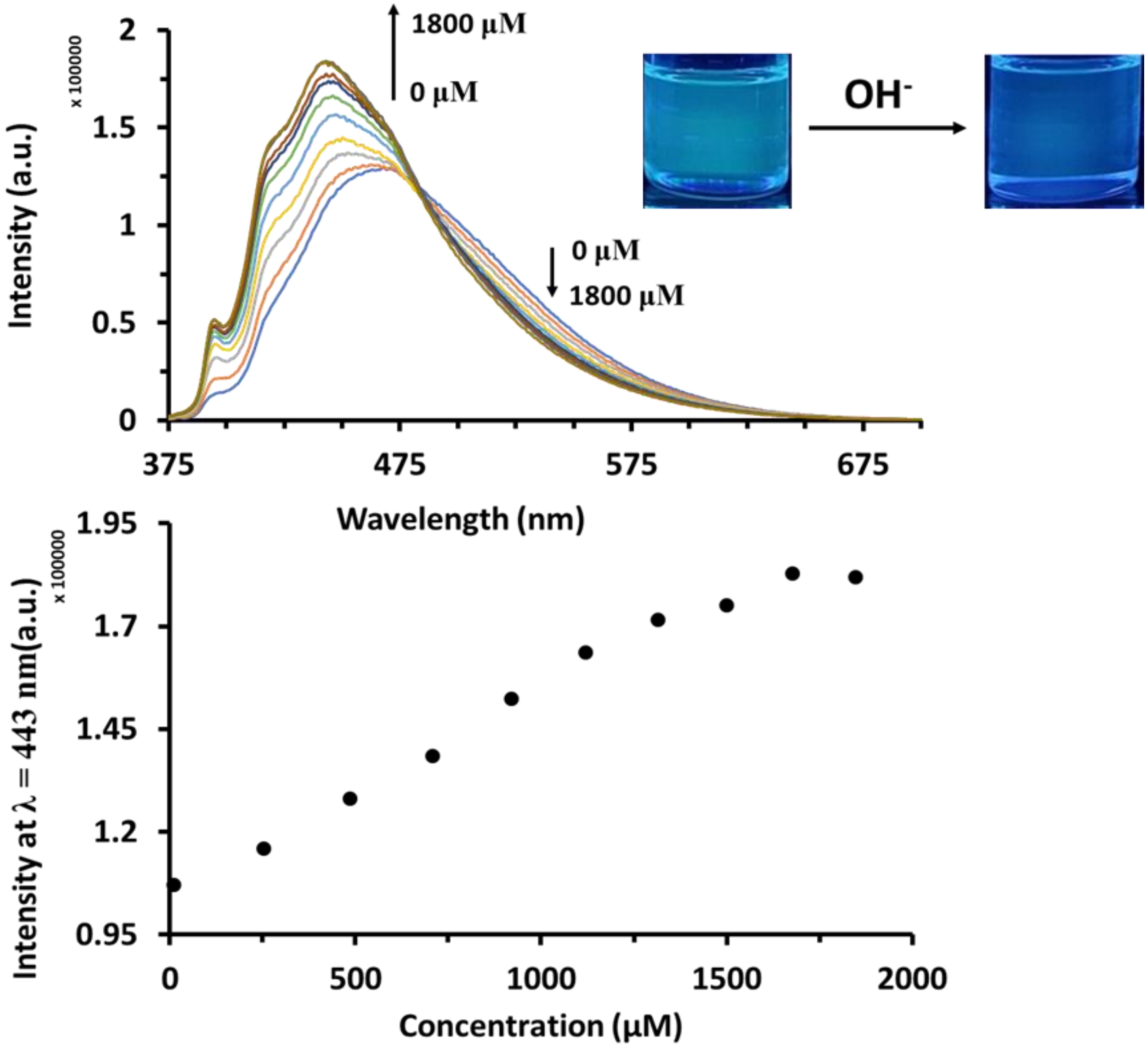


**Figure S7b.** (top) Fluorescent titration result at $\lambda_{ex}$= 370 nm of 6 μM of AnSQ upon the addition of hydroxide ion in DMSO as solvent (bottom) intensity at $\lambda_{em}$ = 443 nm in DMSO

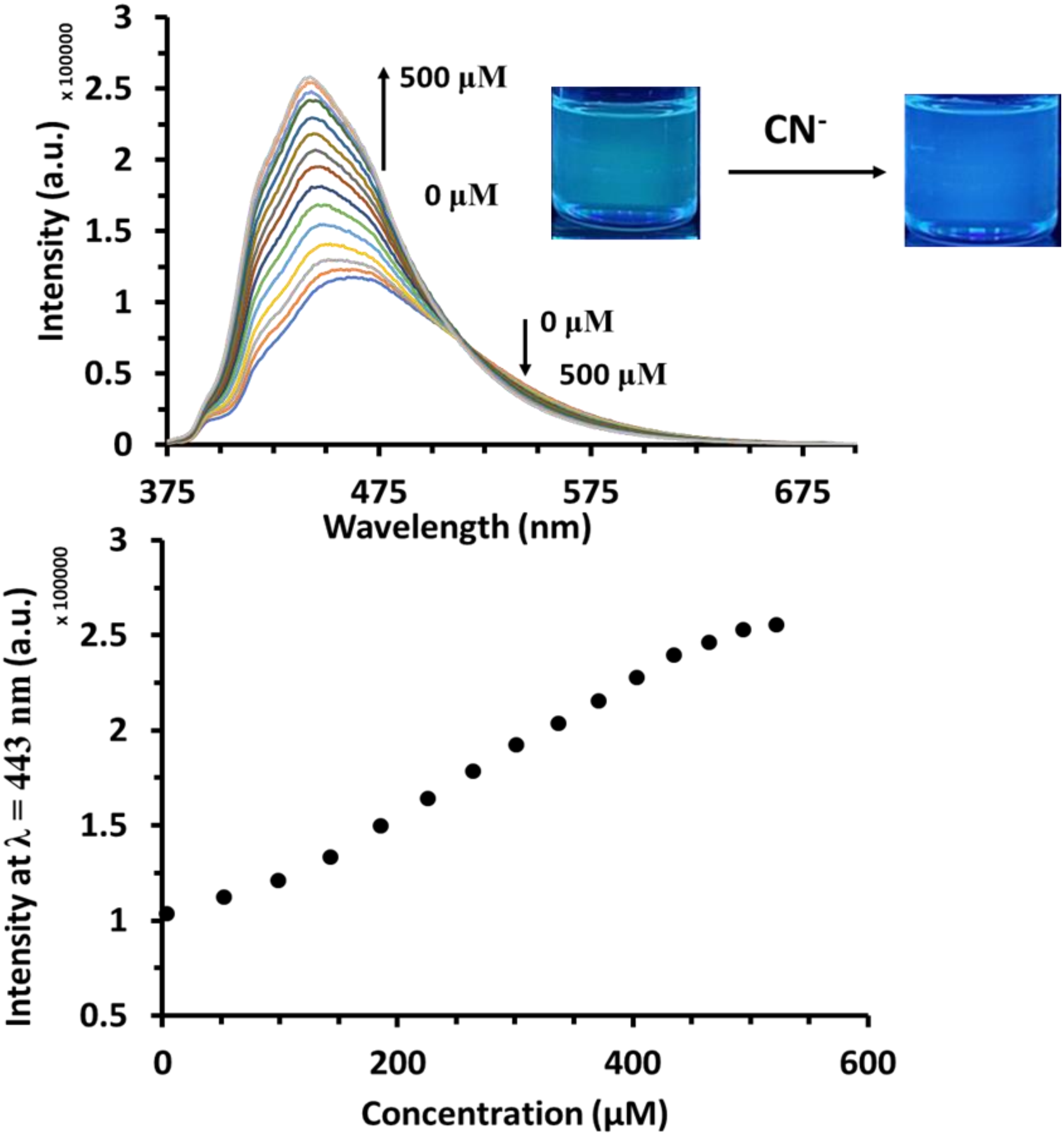


**Figure S7c.** (top) Fluorescent titration result at $\lambda_{ex}$= 370 nm of 6 μM of AnSQ upon the addition of cyanide ion in DMSO as solvent (bottom) intensity at $\lambda_{em}$ = 443 nm in DMSO

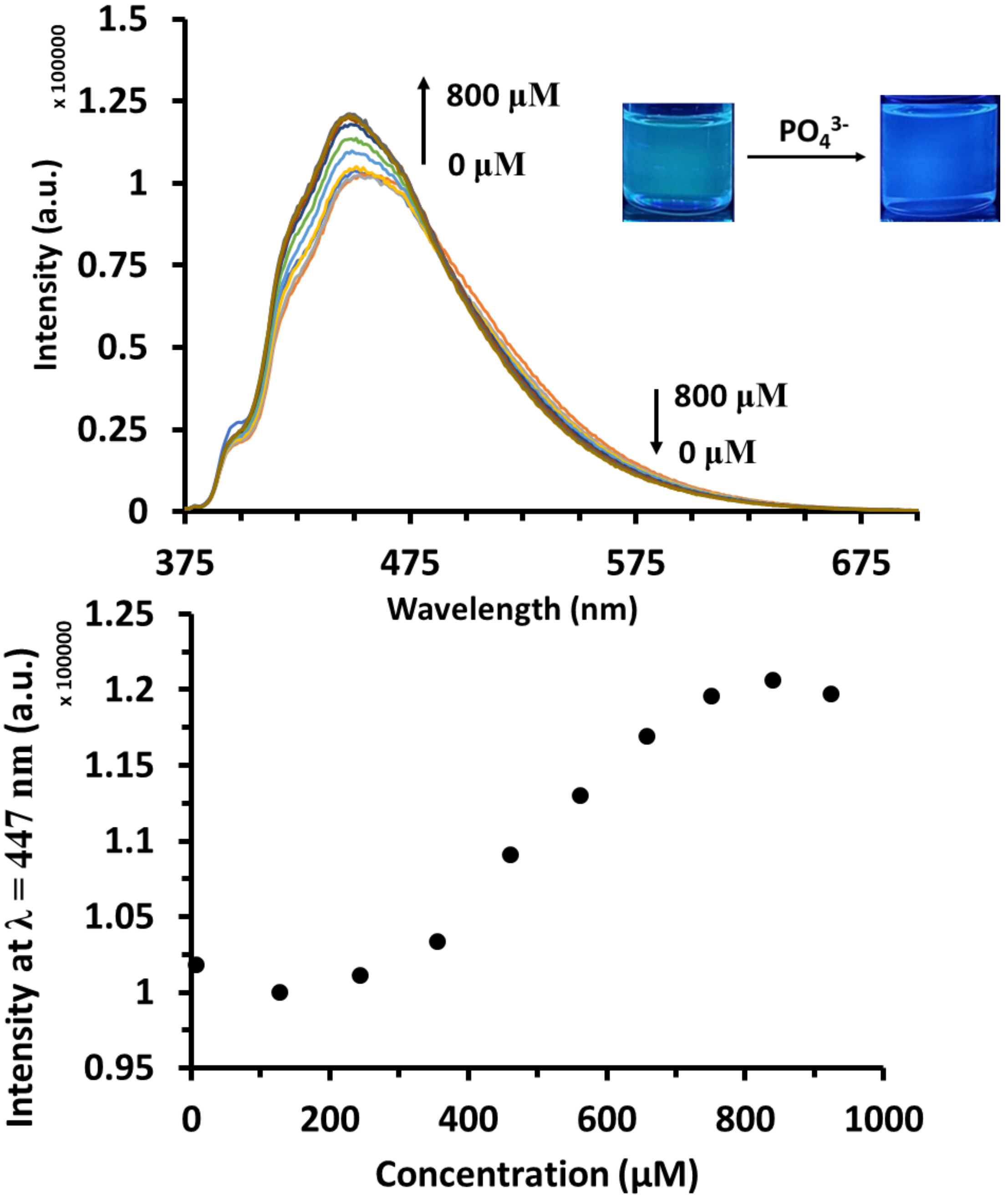


**Figure S7d.** (top) Fluorescent titration result at $\lambda_{ex}$= 370 nm of 6 μM of AnSQ upon the addition of phosphate ion in DMSO as solvent (bottom) intensity at $\lambda_{em}$ = 447 nm in DMSO

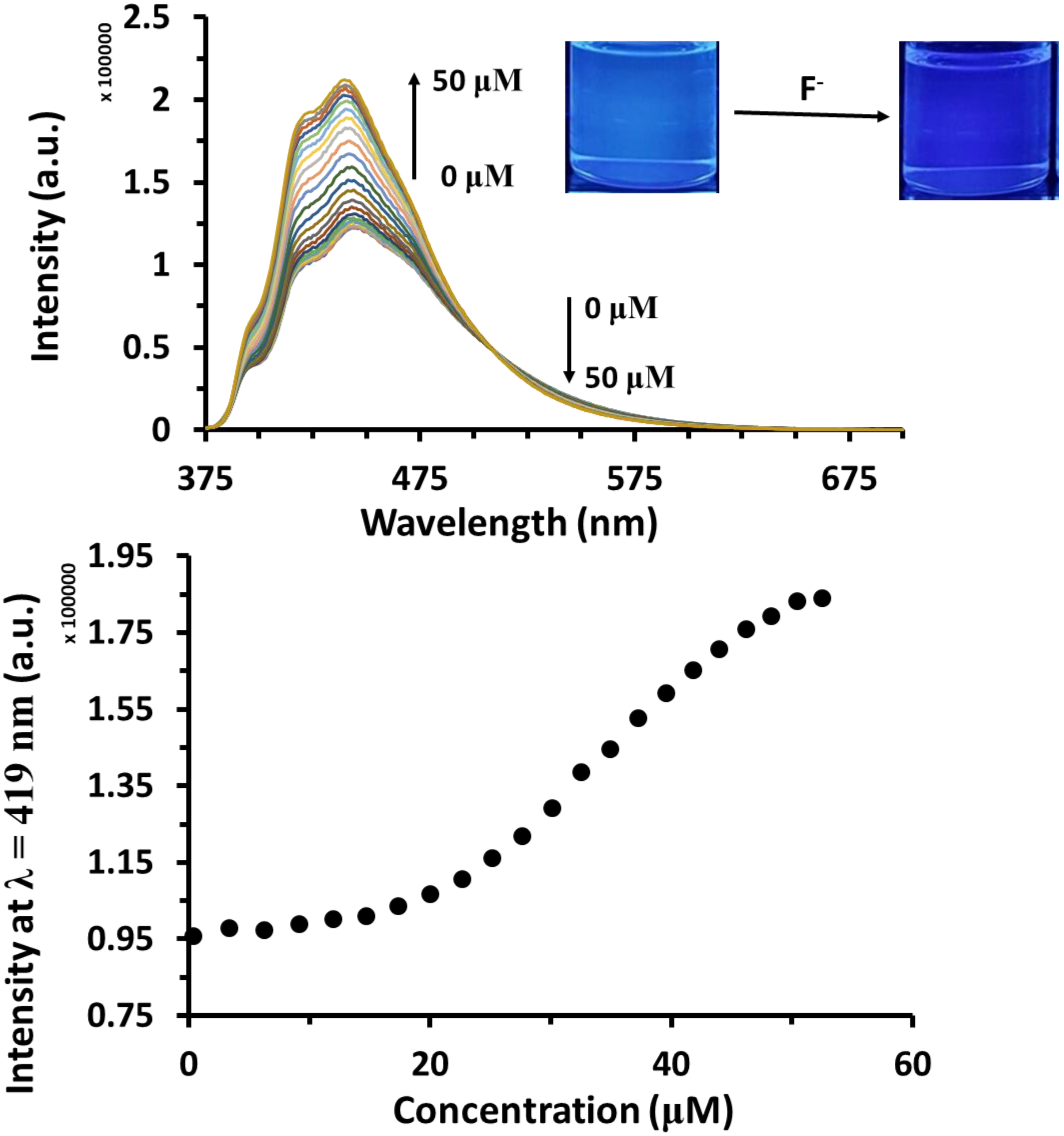


Figure S7e. (top) Fluorescent titration result at $\lambda_{ex}$= 370 nm of 6 μM of AnSQ upon the addition of fluoride ion in DMF as solvent (bottom) intensity at $\lambda_{em}$ = 419 nm

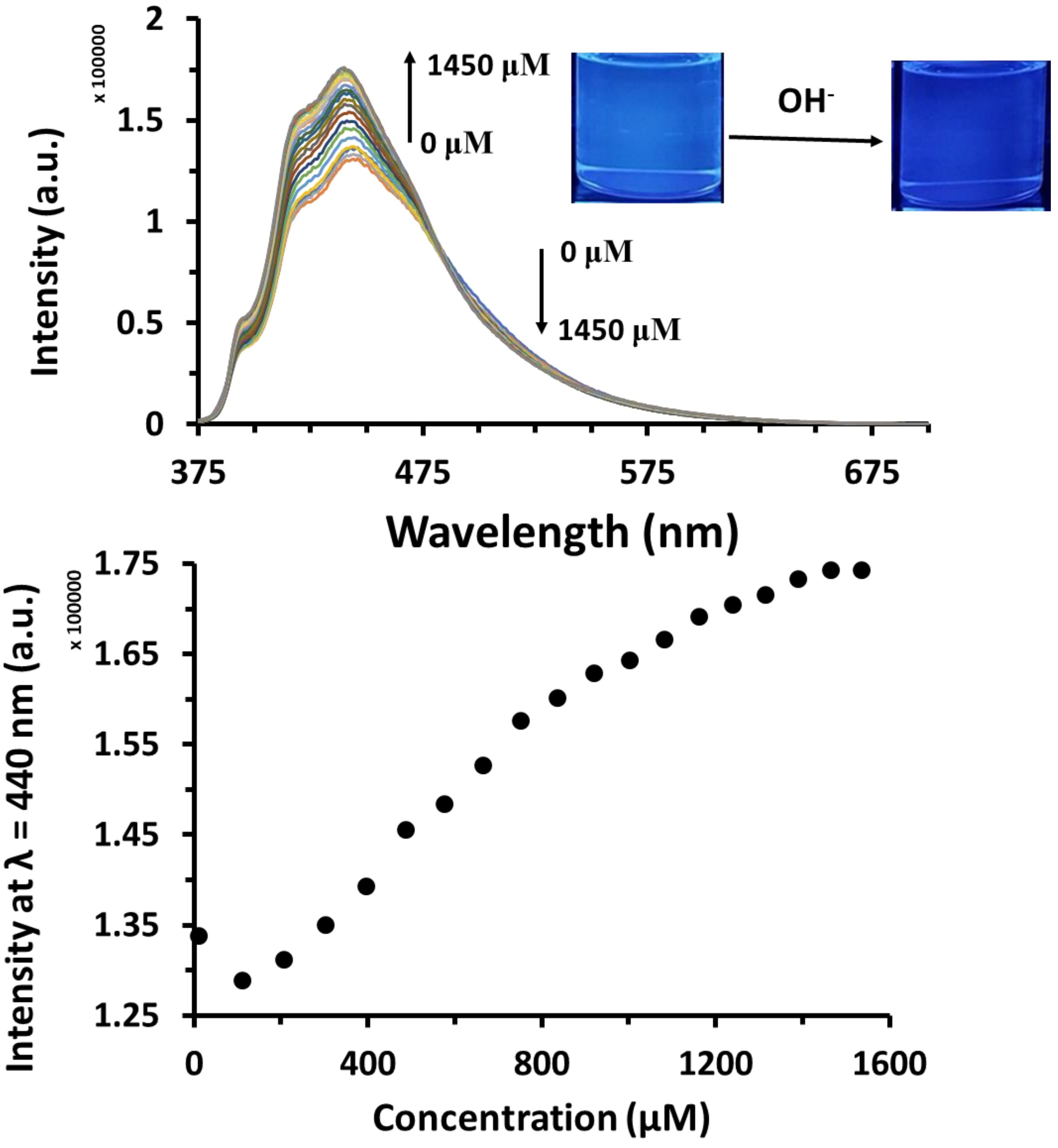


**Figure S7f.** (top) Fluorescent titration result at $\lambda_{ex}$= 370 nm of 6 µM of AnSQ upon the addition of hydroxide ion in DMF as solvent (bottom) intensity at $\lambda_{em}$ = 440 nm

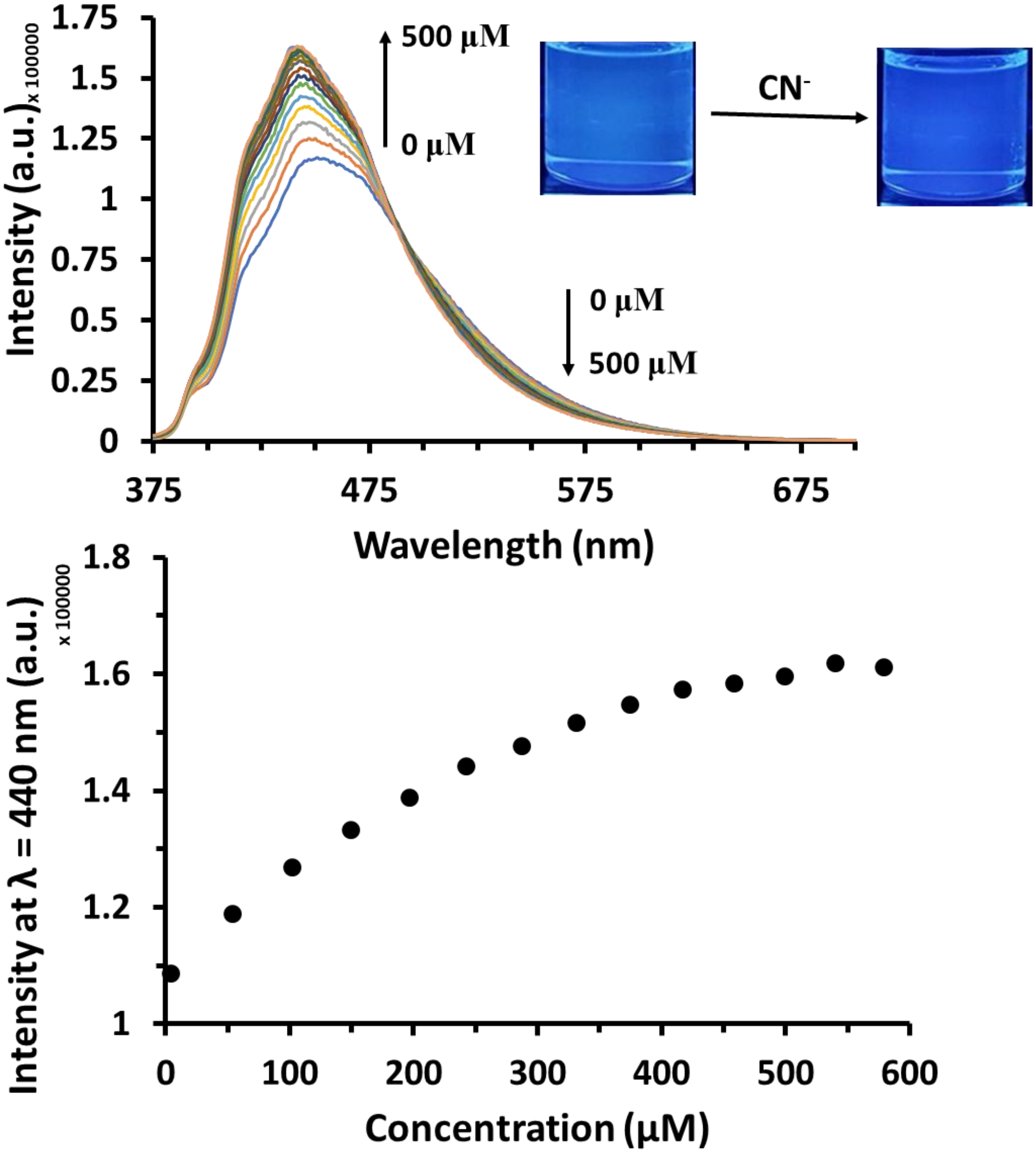


**Figure S7g.** (top) Fluorescent titration result at $\lambda_{ex}$= 370 nm of 6 µM of AnSQ upon the addition of cyanide ion in DMF as solvent (bottom) intensity at $\lambda_{em}$ = 440 nm

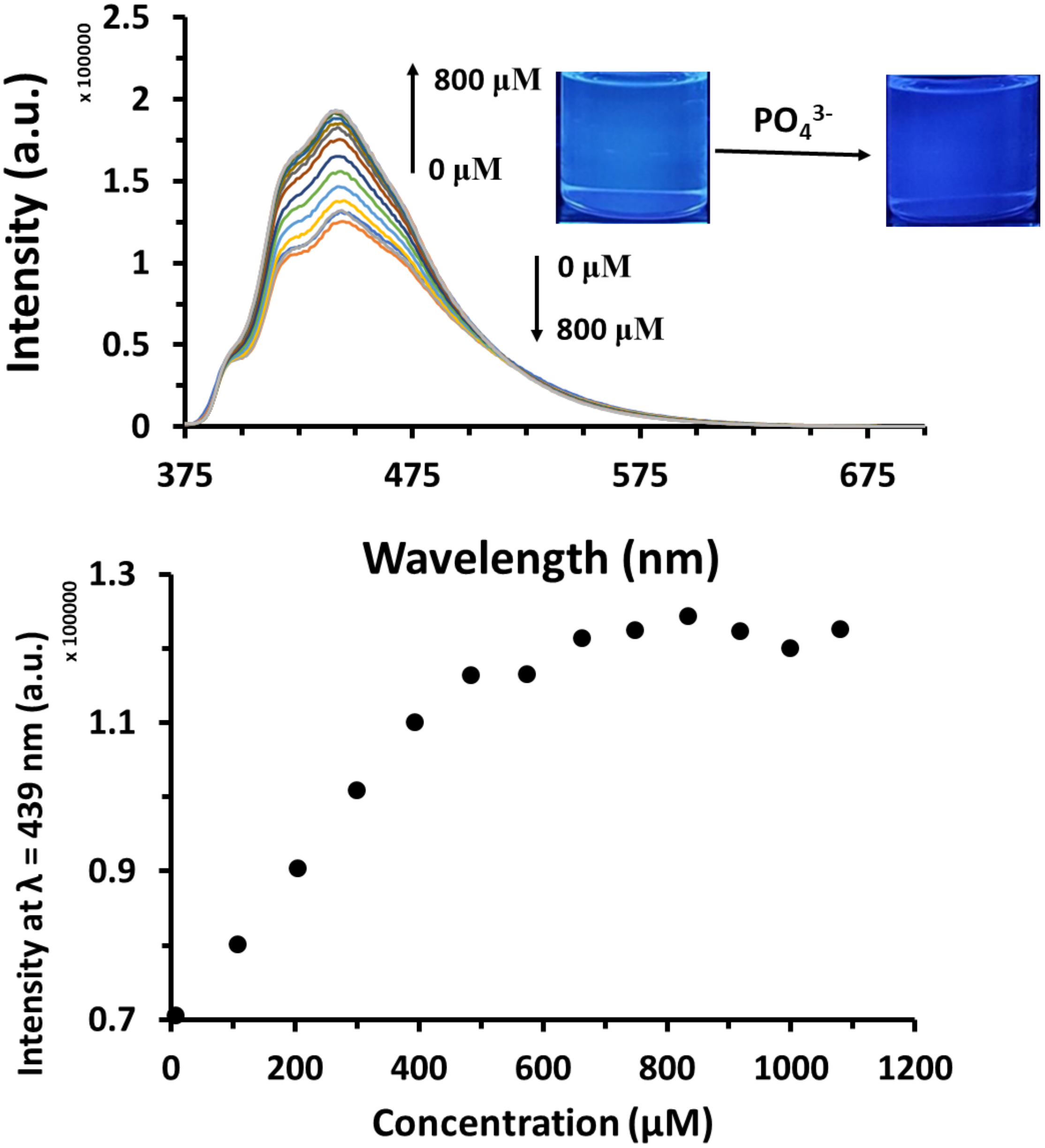


**Figure S7h.** (top) Fluorescent titration result at $\lambda_{ex}$ = 370 nm of 6 μM of AnSQ upon the addition of phosphate ion in DMF as solvent (bottom) intensity at $\lambda_{em}$ = 439 nm

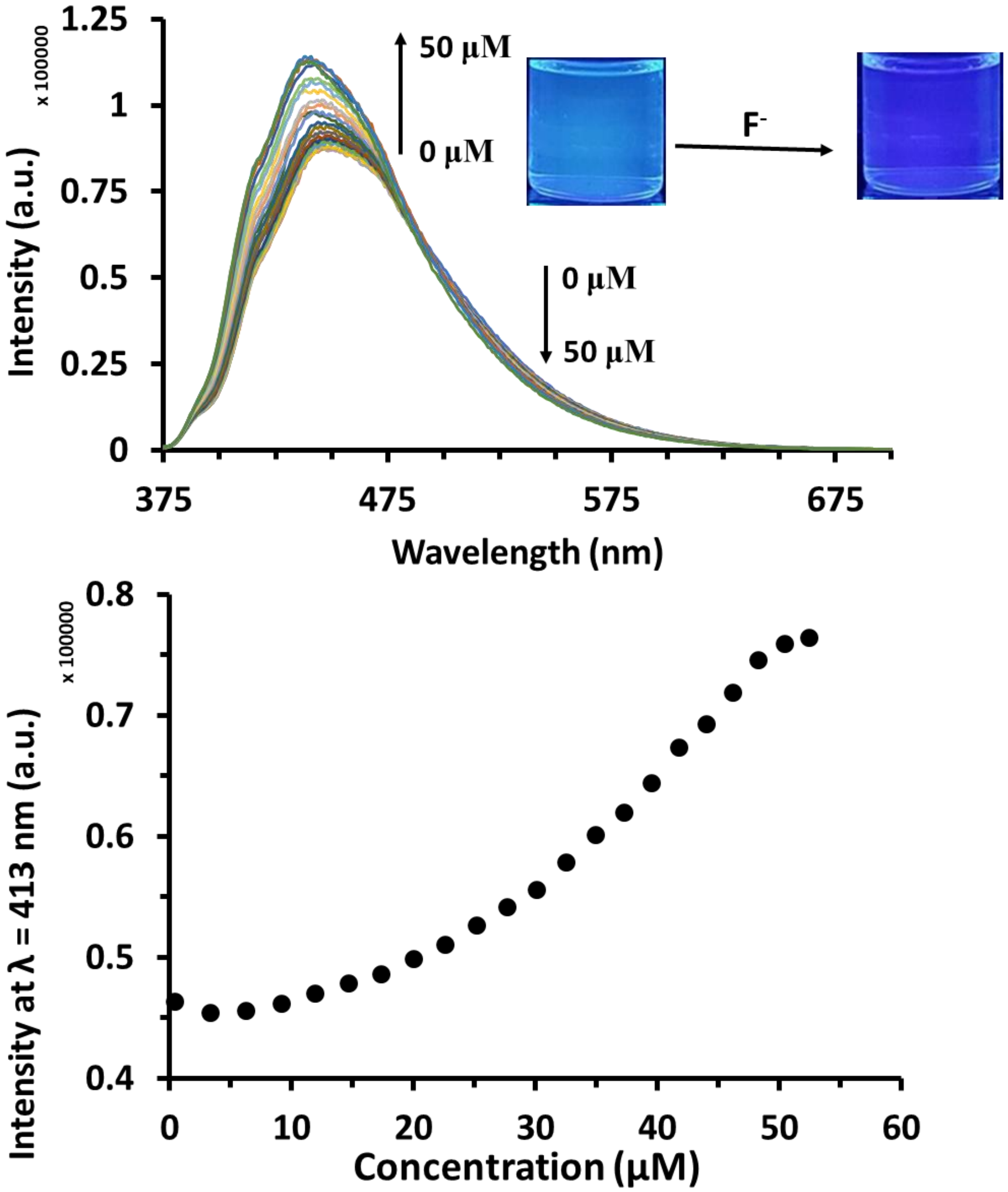


**Figure S7i.** (top) Fluorescent titration result at $\lambda_{ex}$= 370 nm of 6 μM of AnSQ upon the addition of fluoride ion in acetone as solvent (bottom) intensity at $\lambda_{em}$ = 413 nm

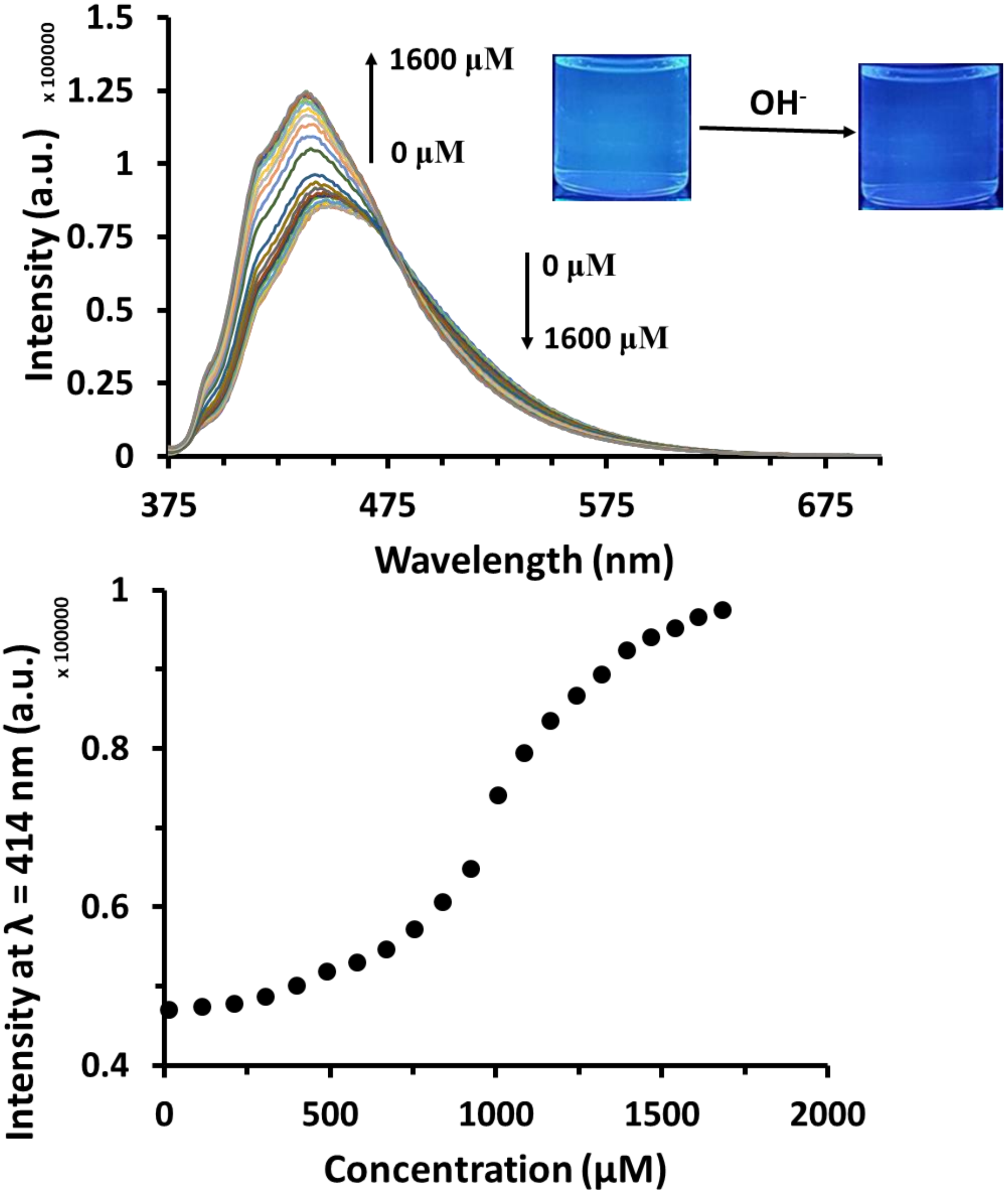


**Figure S7k.** (top) Fluorescent titration result at $\lambda_{ex}$= 370 nm of 6 μM of AnSQ upon the addition of hydroxide ion in acetone as solvent (bottom) intensity at $\lambda_{em}$ = 414 nm

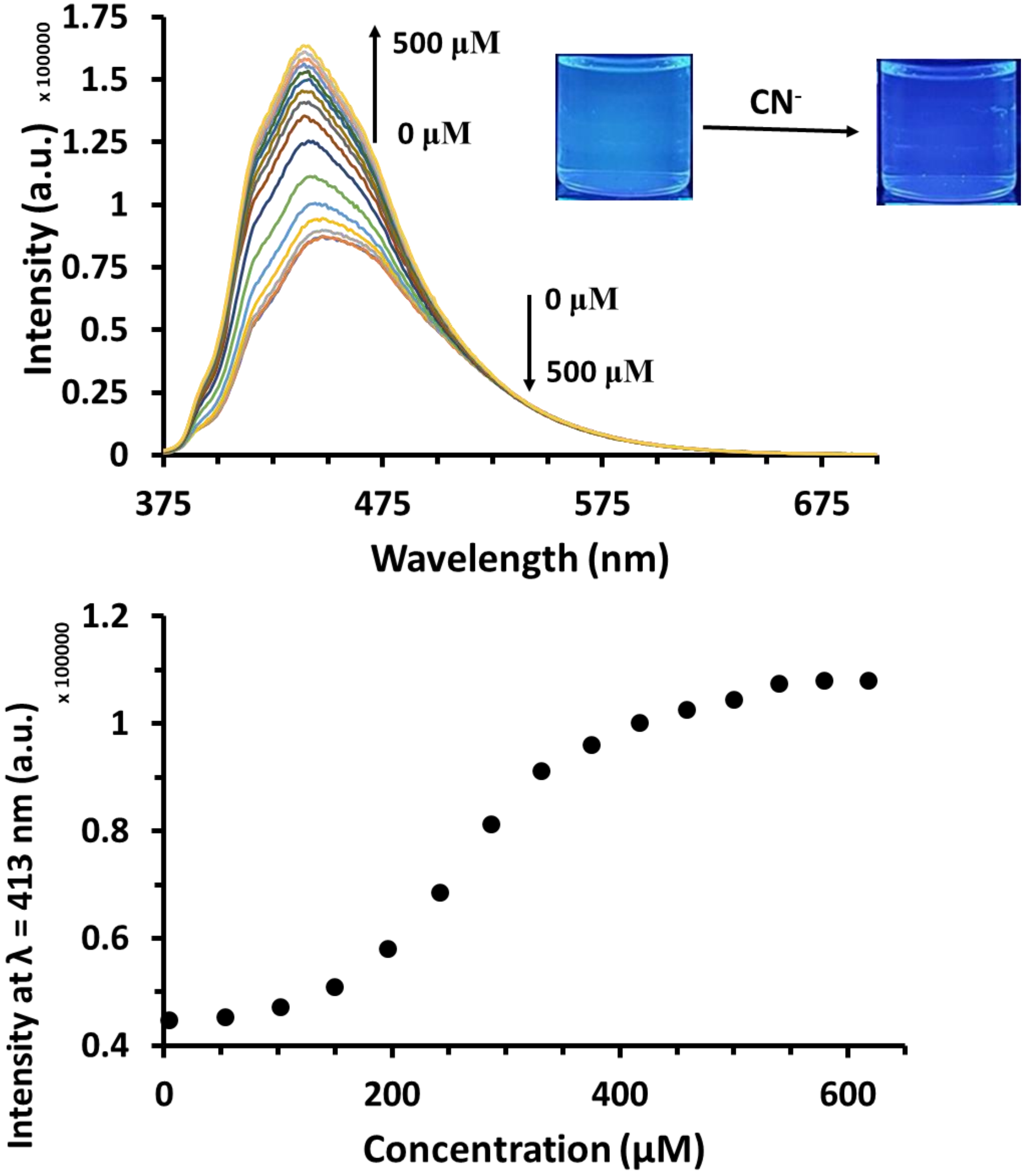


**Figure S7l.** (top) Fluorescent titration result at $\lambda_{ex}$= 370 nm of 6 µM of AnSQ upon the addition of cyanide ion in acetone as solvent (bottom) intensity at $\lambda_{em}$ = 443 nm

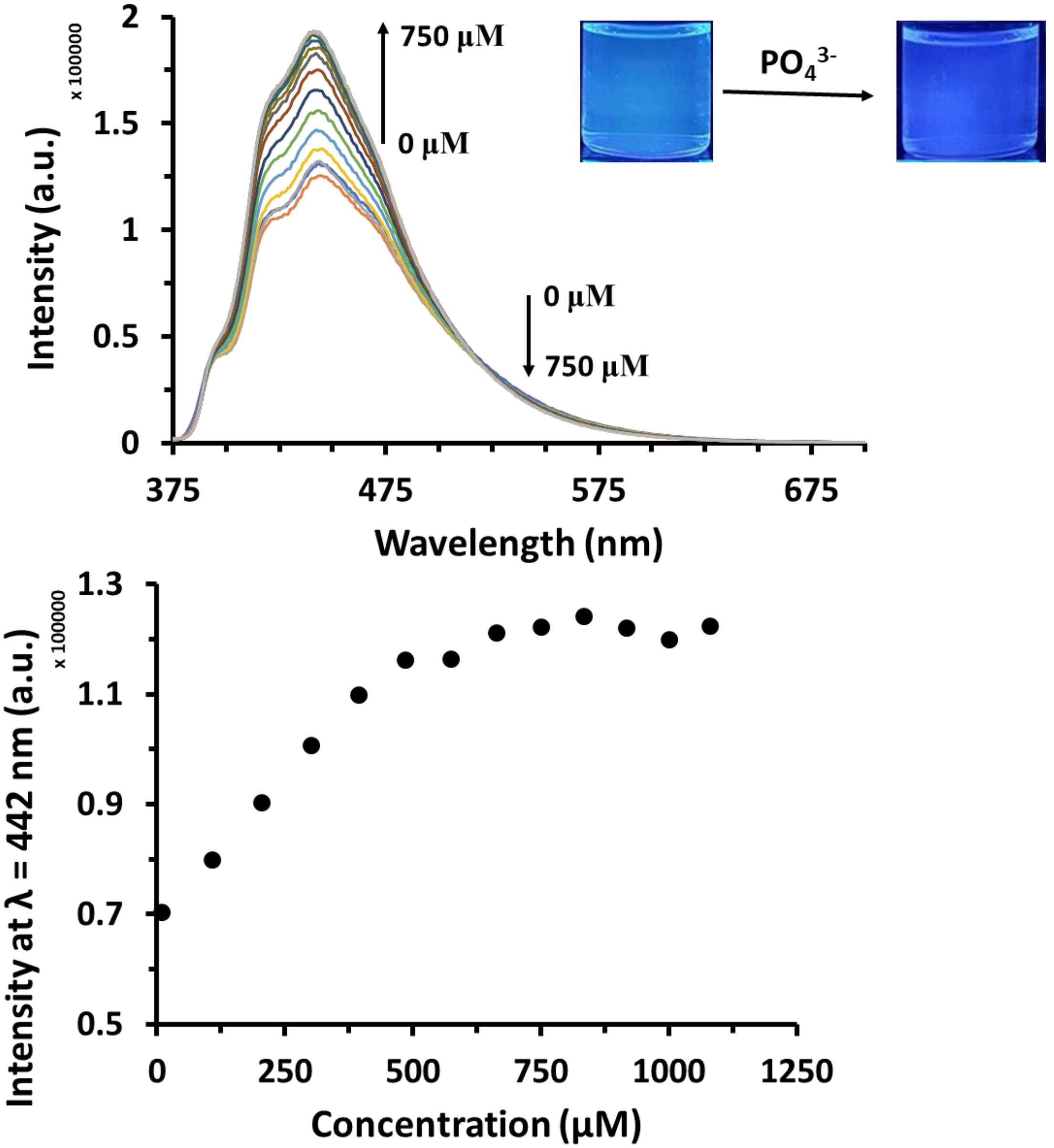


**Figure S7m.** (top) Fluorescent titration result at $\lambda_{ex}$= 370 nm of 6 μM of AnSQ upon the addition of phosphate ion in DMSO as solvent (bottom) intensity at $\lambda_{em}$ = 442 nm

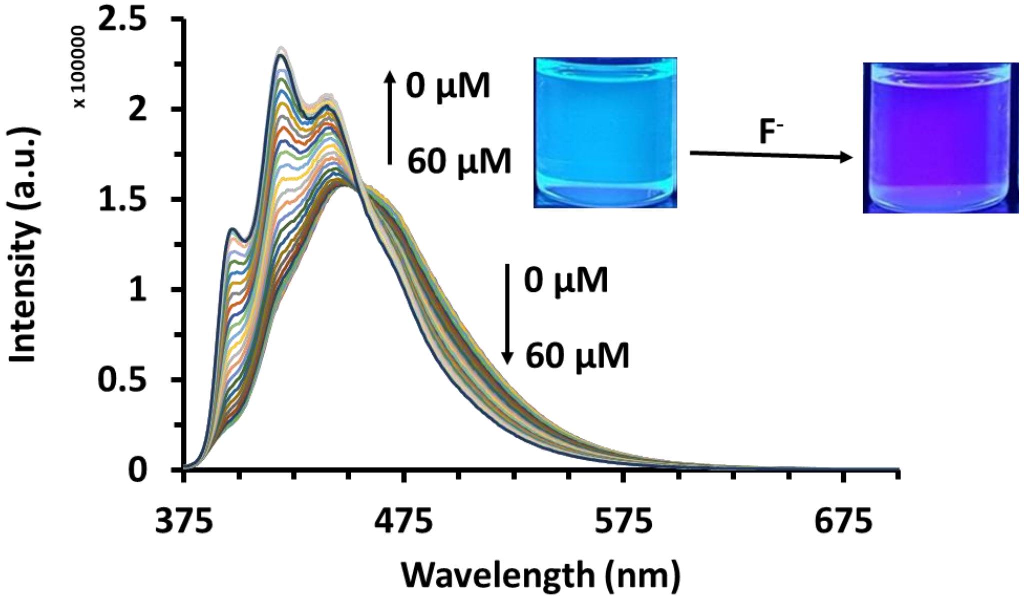


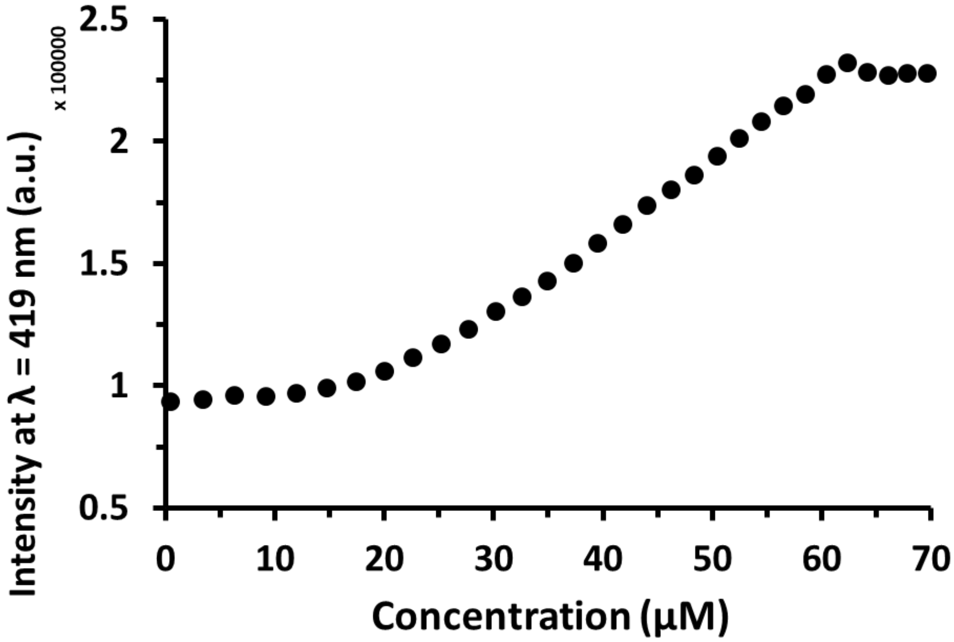


**Figure S7n.** (top) Fluorescent titration result at $\lambda_{ex}$= 370 nm of 6 μM of AnSQ upon the addition of cyanide ion in Toluene as solvent (bottom) intensity at $\lambda_{em}$ = 419 nm

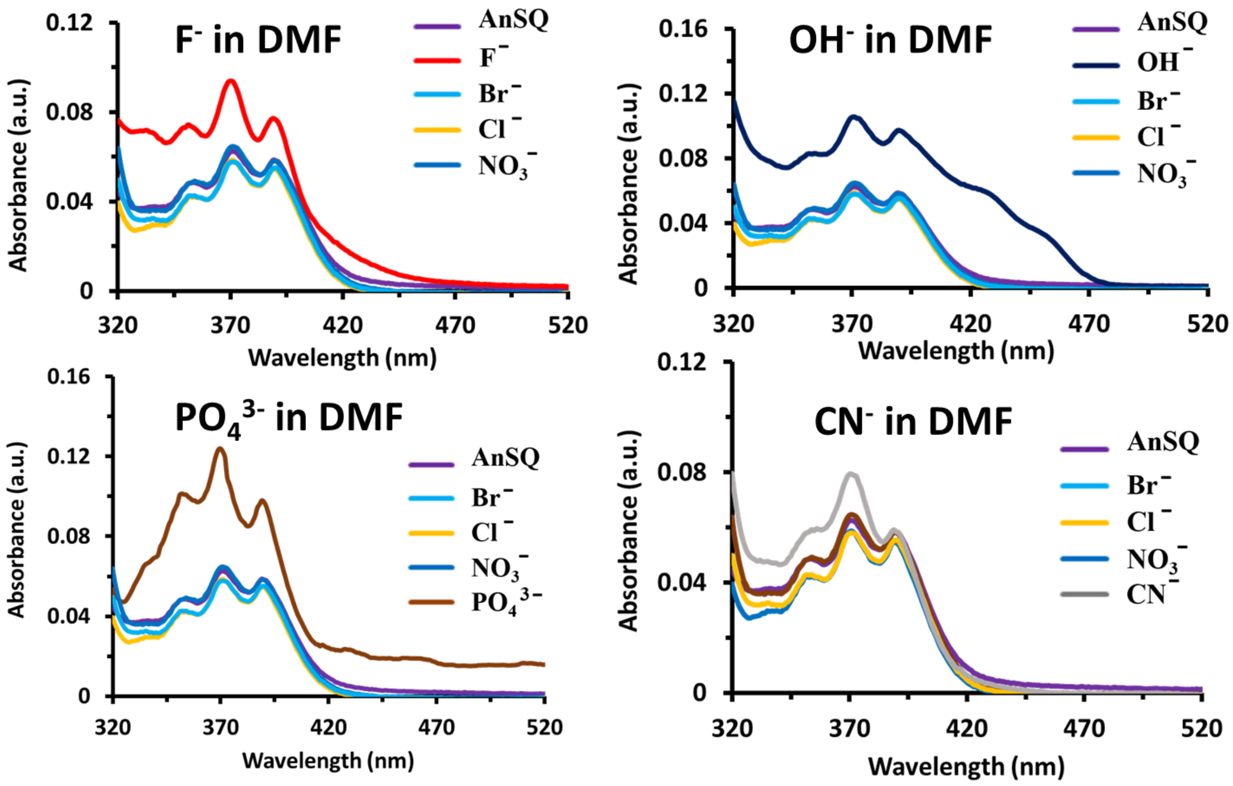


**Figure S8a.** UV-vis absorption of 6 μM AnSQ after anion addition in DMF with 18 hr reaction time.

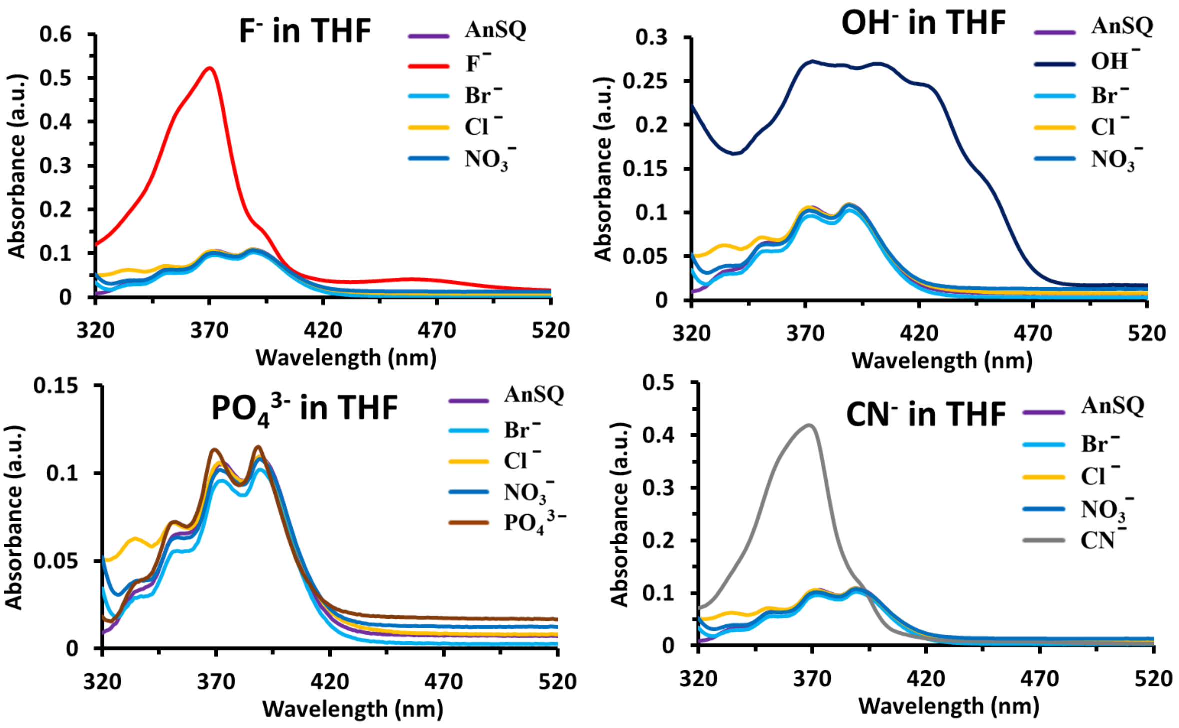


**Figure S8b.** UV-vis absorption of 6 μM AnSQ after anion addition in THF with 18 hr reaction time.

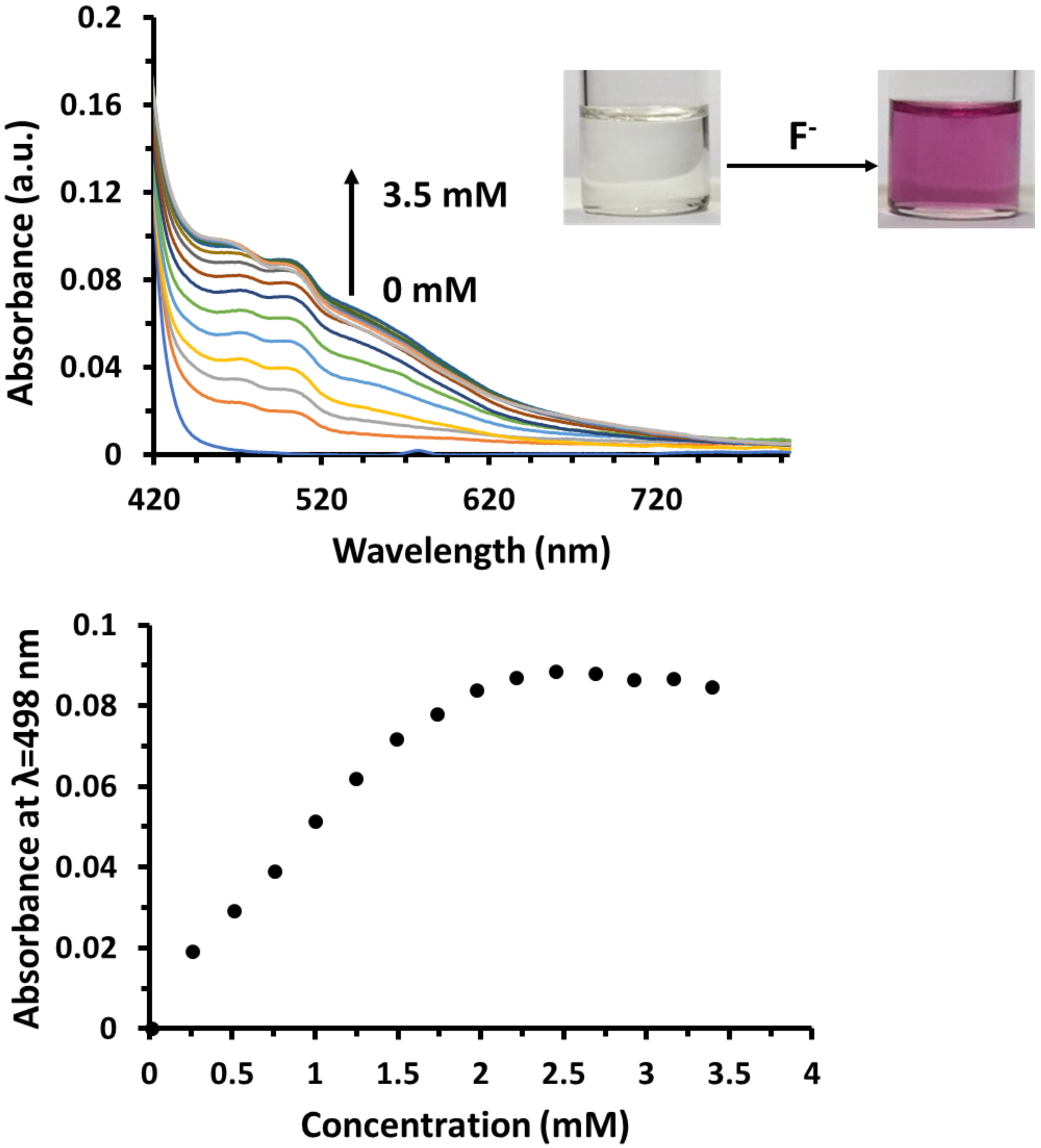


**Figure S8c.** (top) UV titration result of 60 μM of AnSQ upon the addition of fluoride ion in THF as solvent (bottom) absorbance intensity at $\lambda_{max}$ = 498 nm

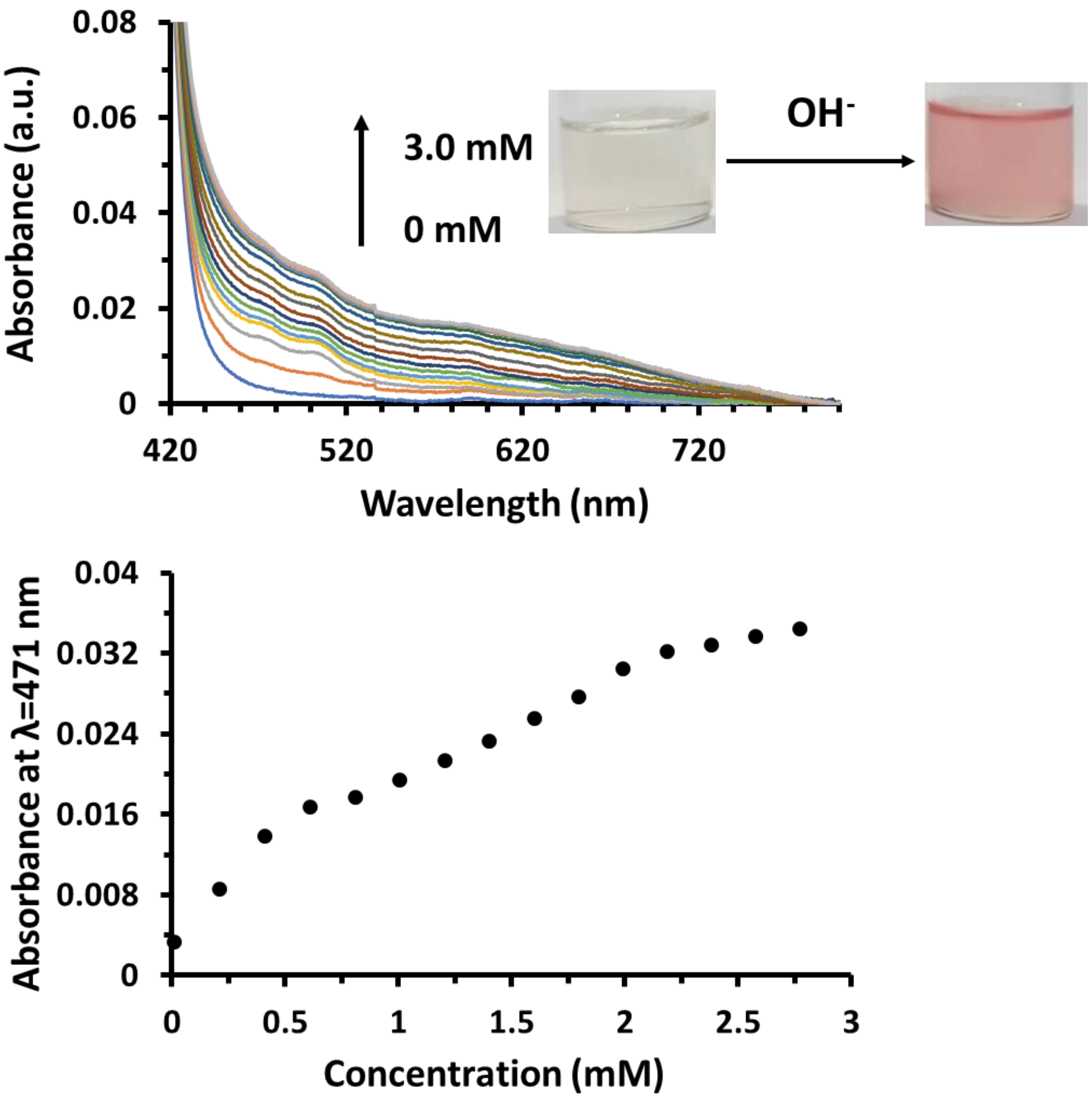


**Figure S8e.** (top) UV titration result of 60 μM of AnSQ upon the addition of hydroxide ion in THF as solvent (bottom) intensity at $\lambda_{max}$ = 471 nm

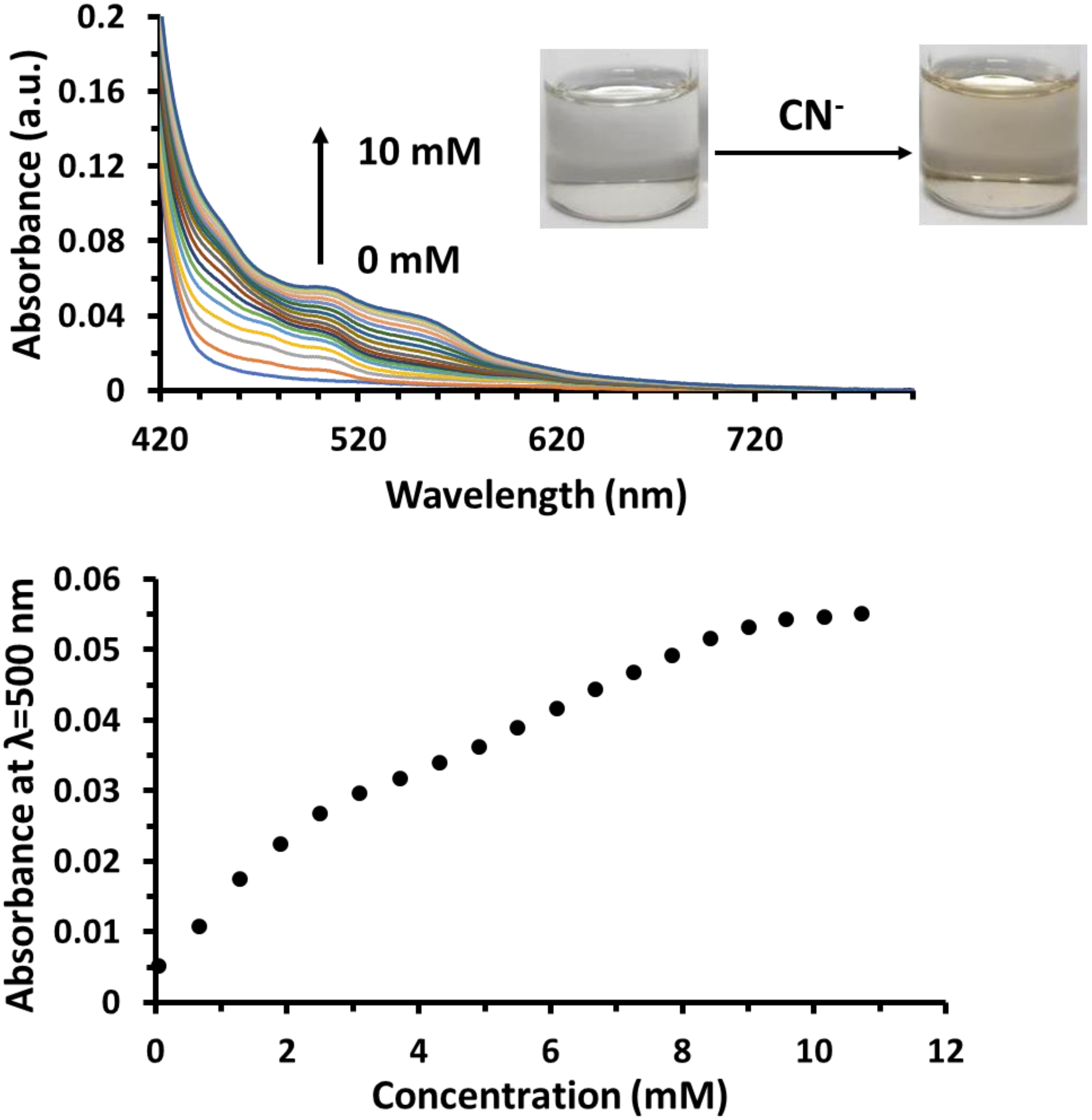


**Figure S8f.** (top) UV titration result of 60 μM of AnSQ upon the addition of cyanide ion in THF as solvent (bottom) intensity at $\lambda_{max}$ = 500 nm

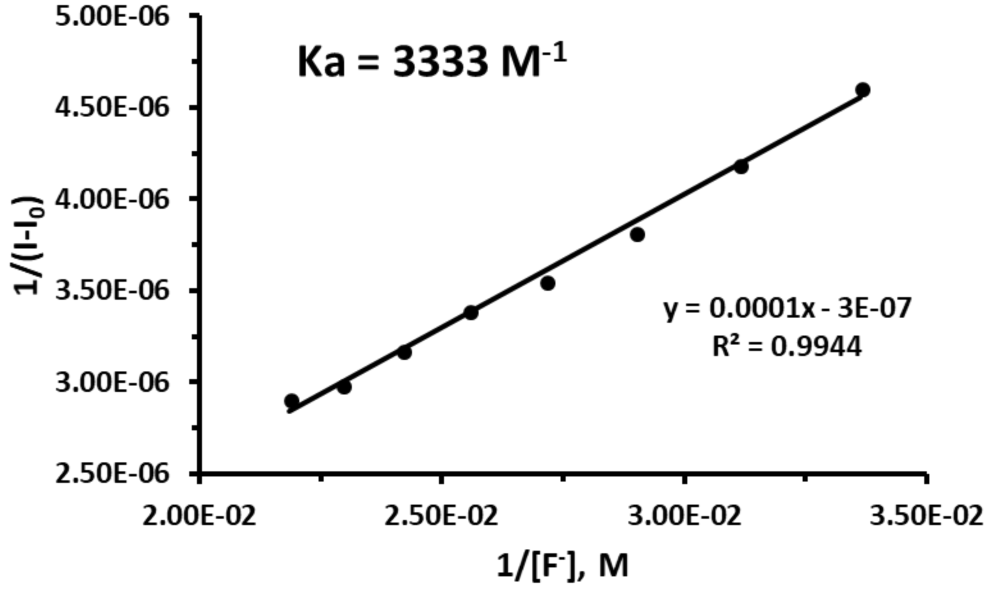


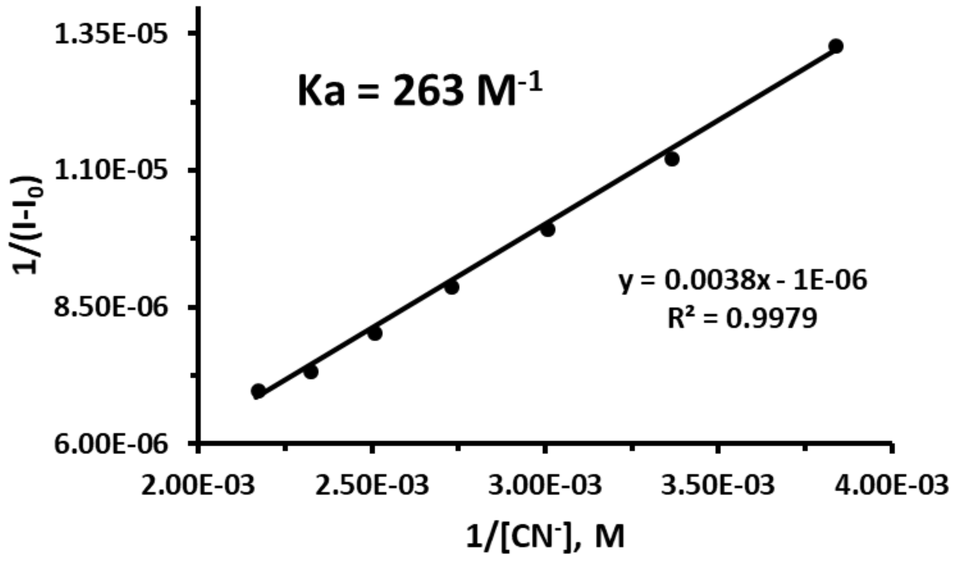


**Figure S9a.** Benesi-Hildebrand plot from fluorescent data of AnSQ upon addition of $F^-$ and $CN^-$

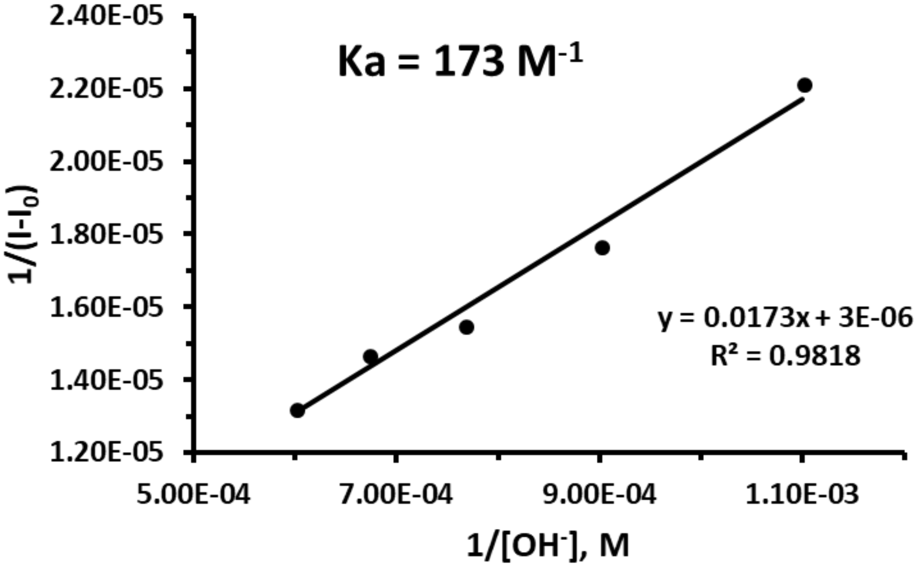


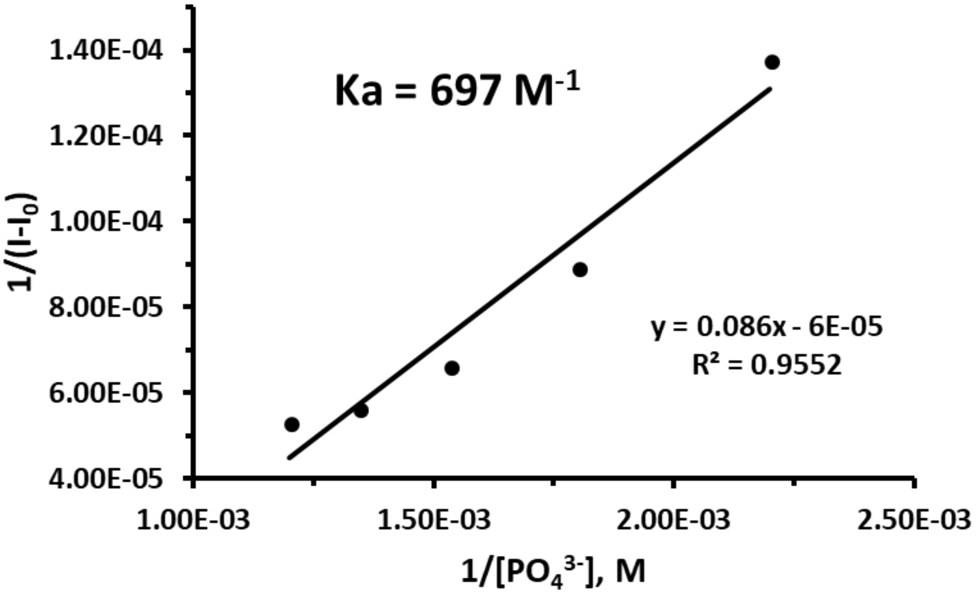


**Figure S9b.** Benesi-Hildebrand plot from fluorescent data of AnSQ upon addition of $OH^-$ and $PO_4^{3-}$ for association constant ($K_a$)

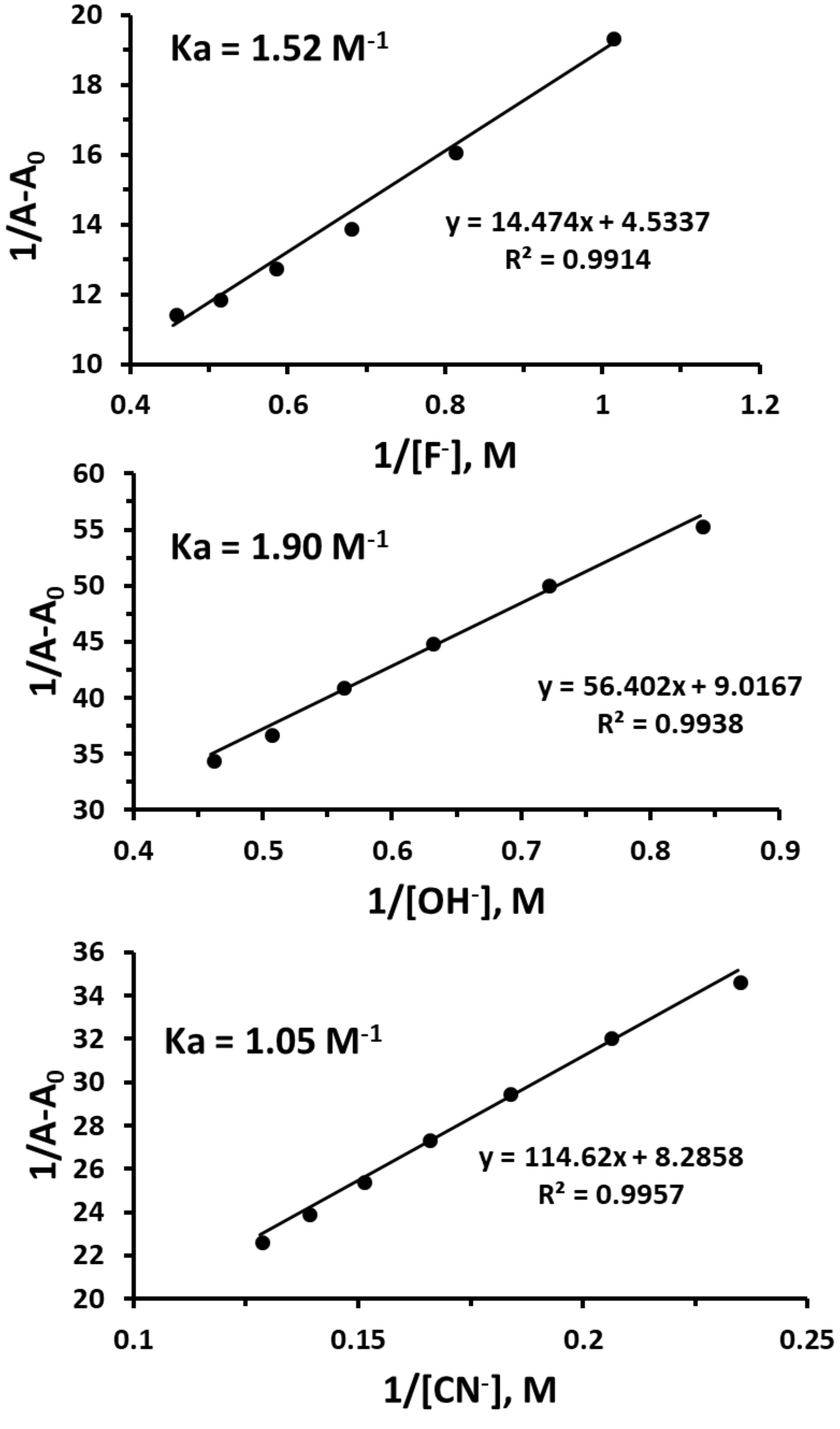


**Figure S10a.** Benesi-Hildebrand plot from absorbance data of AnSQ upon addition of $F^-$, $OH^-$ and $CN^-$ for association constant *($K_a$)*

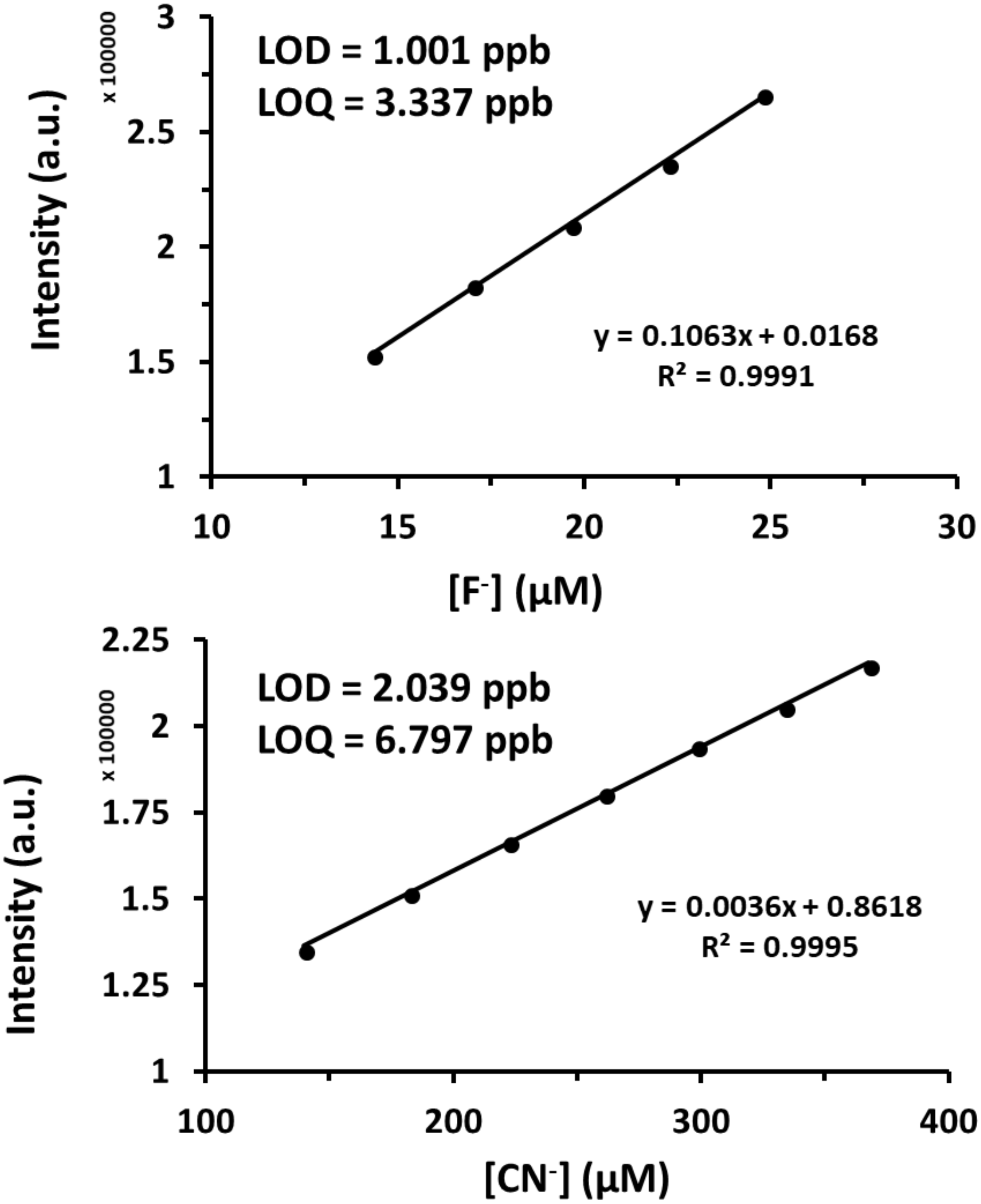


**Figure S10b.** Calibration curve from AnSQ fluorescence upon addition of $F^-$ and $OH^-$ for limit of detection (LOD) and limit of qualitative (LOQ) calculation in DMSO

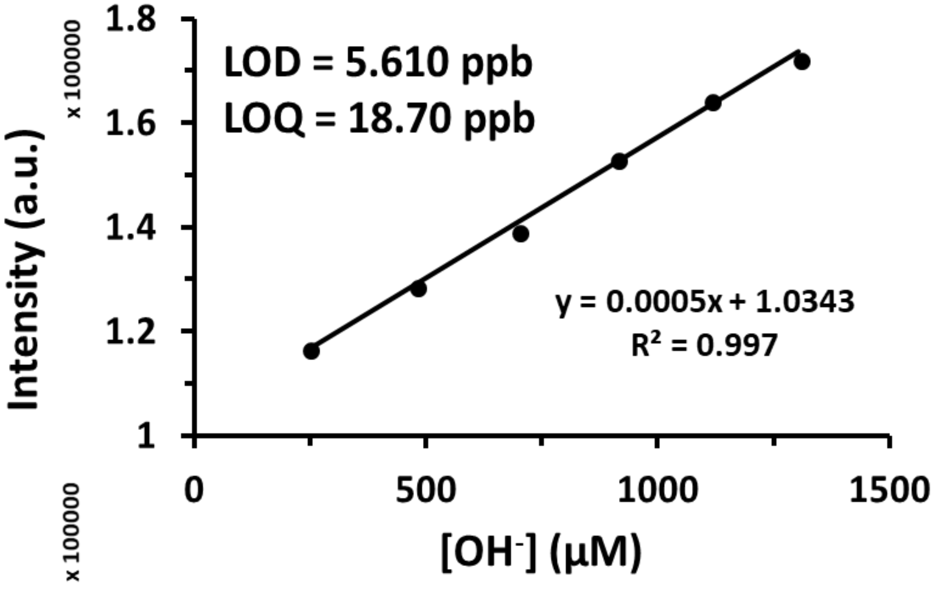


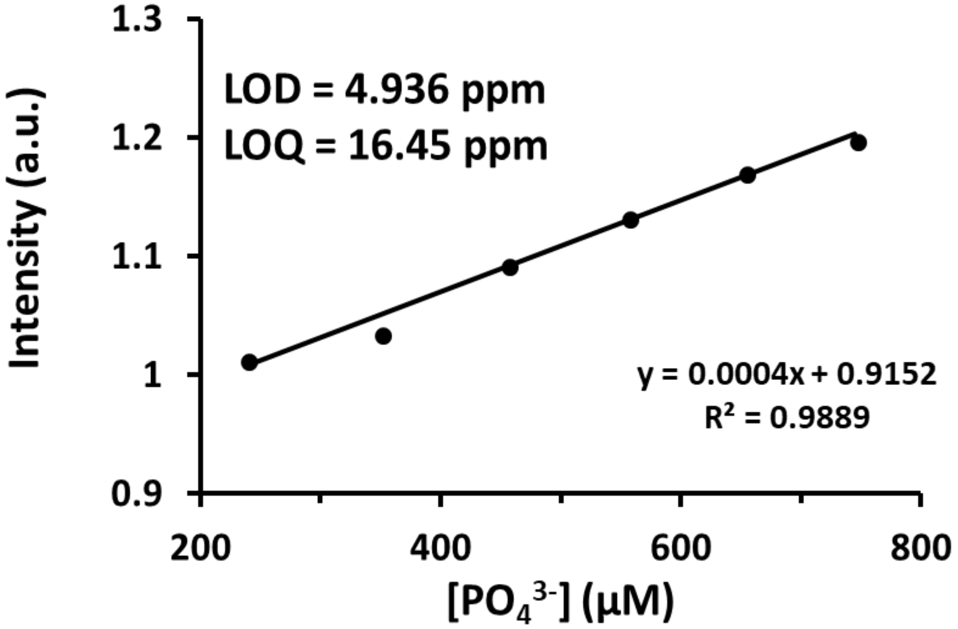


**Figure S10c.** Calibration curve from AnSQ fluorescence upon addition of $OH^-$ and $PO_4^{3-}$ for limit of detection (LOD) and limit of qualitative (LOQ) calculation in DMSO

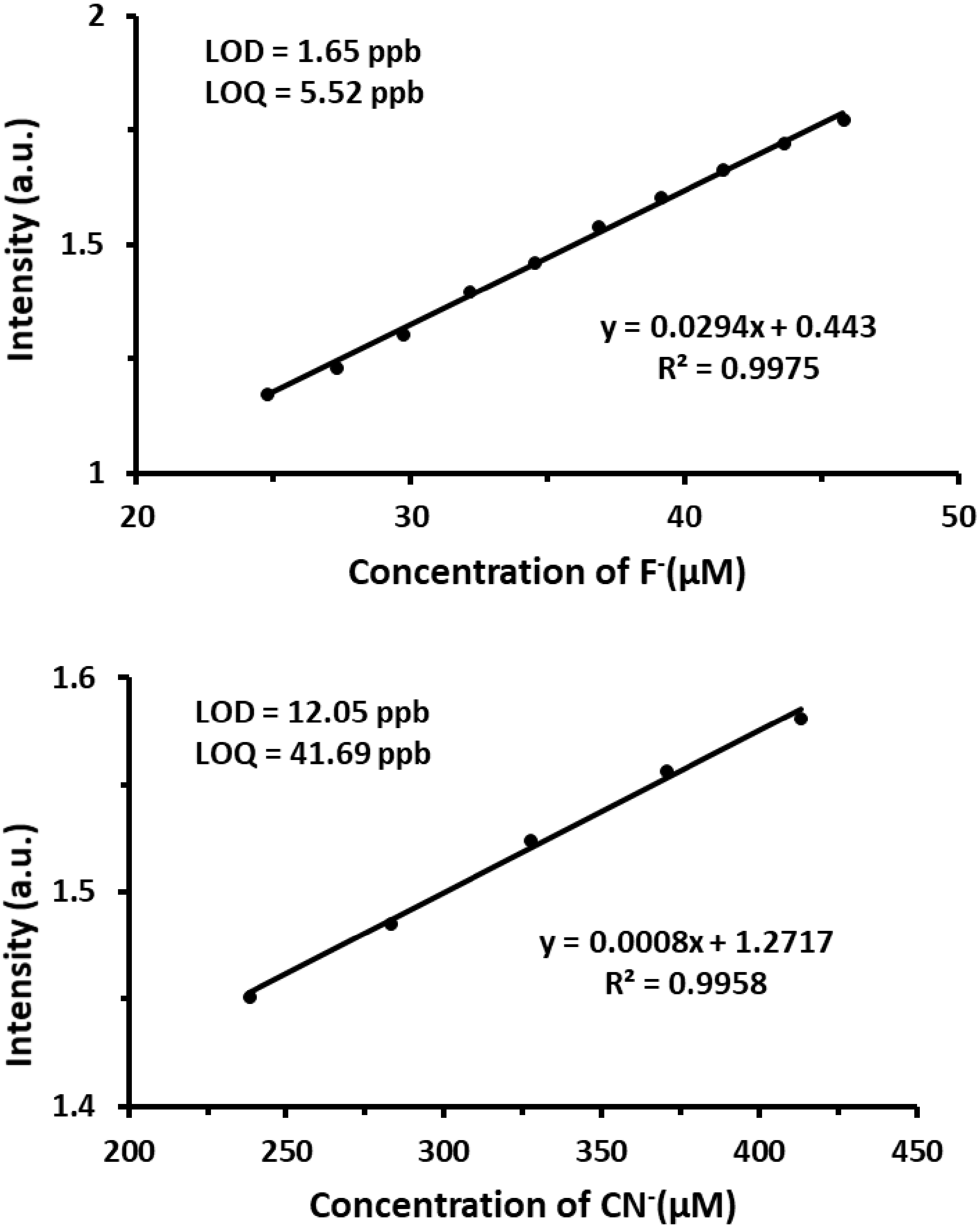


**Figure S10d.** Calibration curve from AnSQ fluorescence upon addition of $F^-$ and $CN^-$ for limit of detection (LOD) and limit of qualitative (LOQ) calculation in DMF

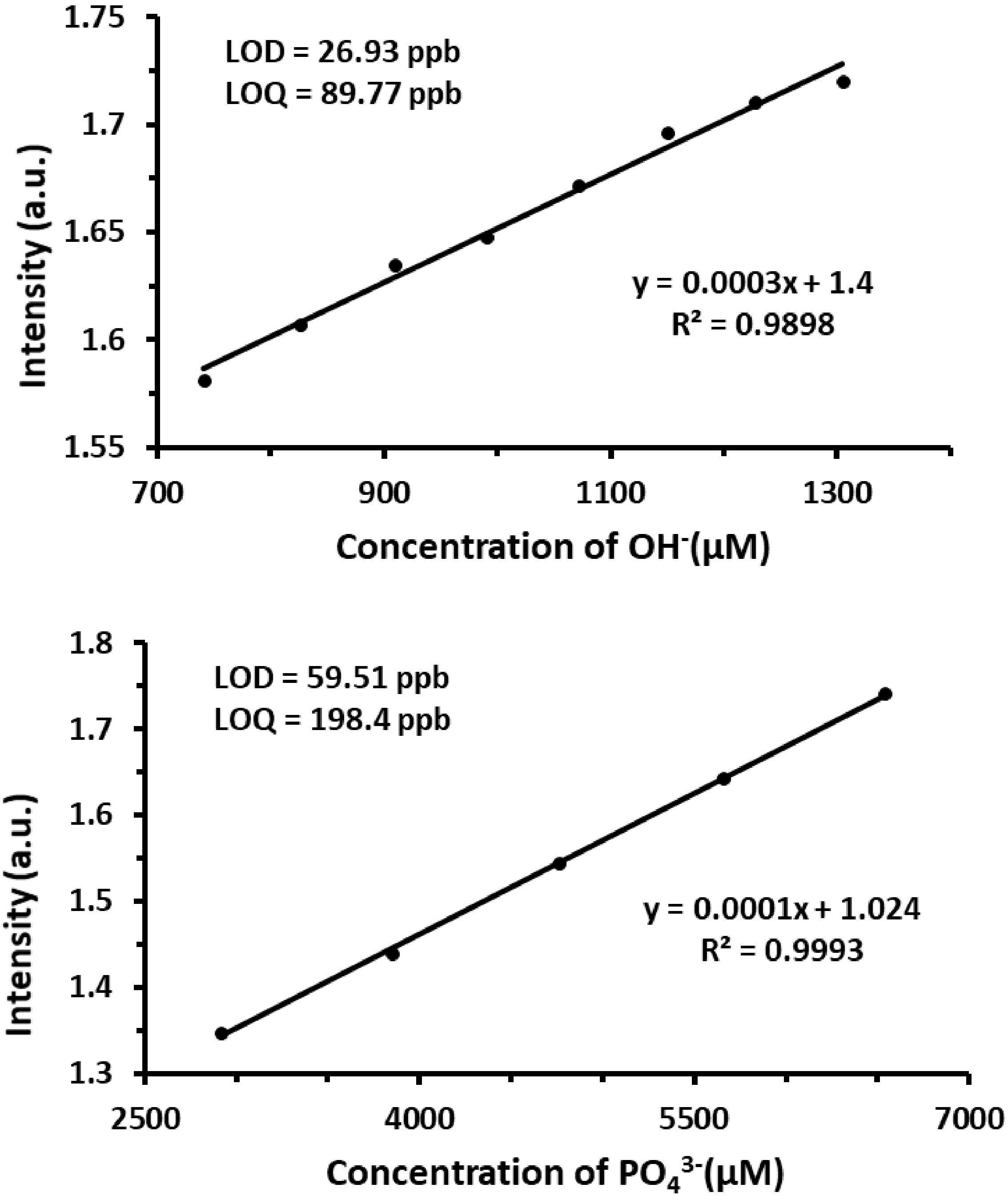


**Figure S10e.** Calibration curve from AnSQ fluorescence upon addition of $OH^-$ and $PO_4^{3-}$ for limit of detection (LOD) and limit of qualitative (LOQ) calculation in DMF

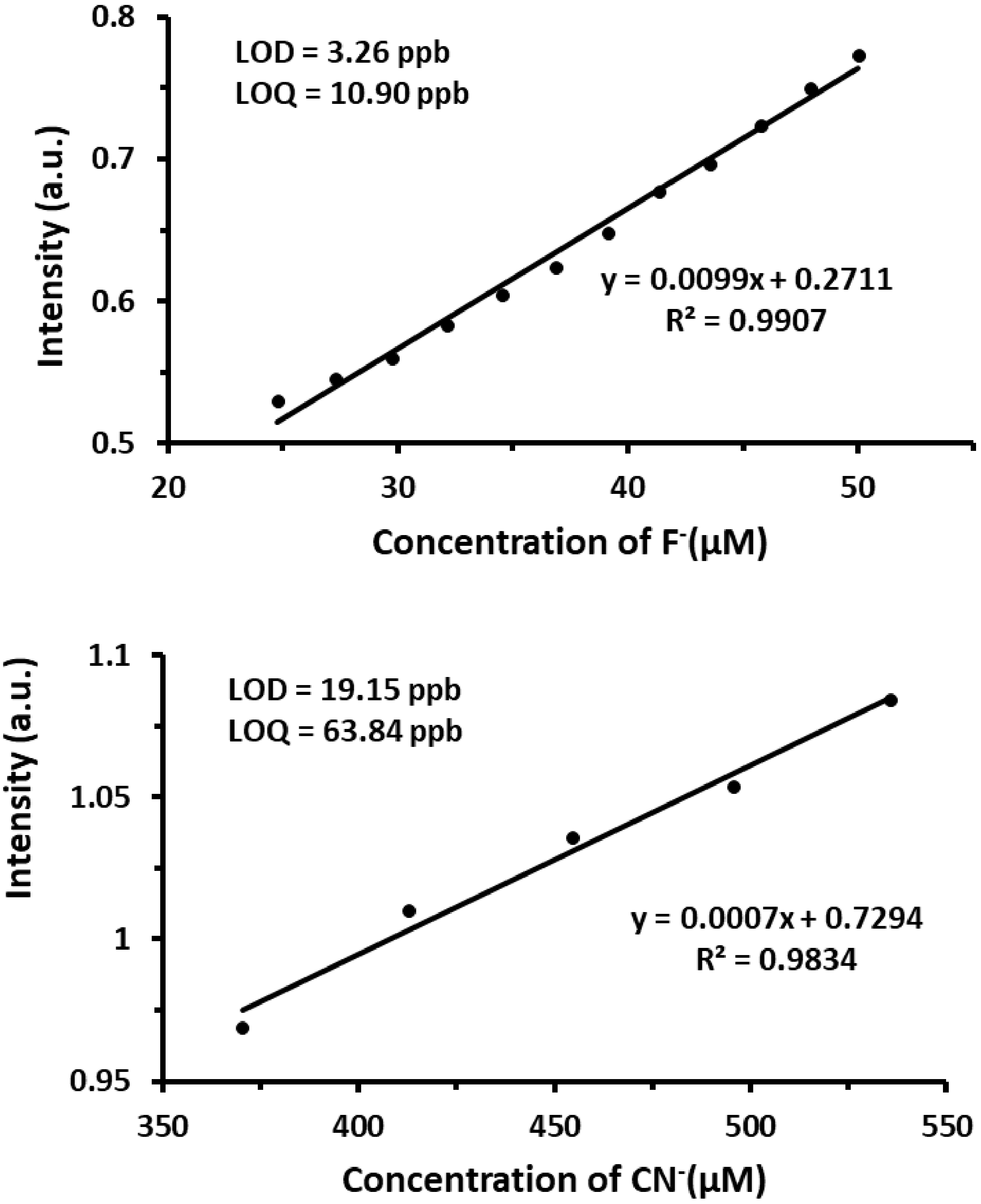


**Figure S10f.** Calibration curve from AnSQ fluorescence upon addition of $F^-$ and $CN^-$ for limit of detection (LOD) and limit of qualitative (LOQ) calculation in acetone

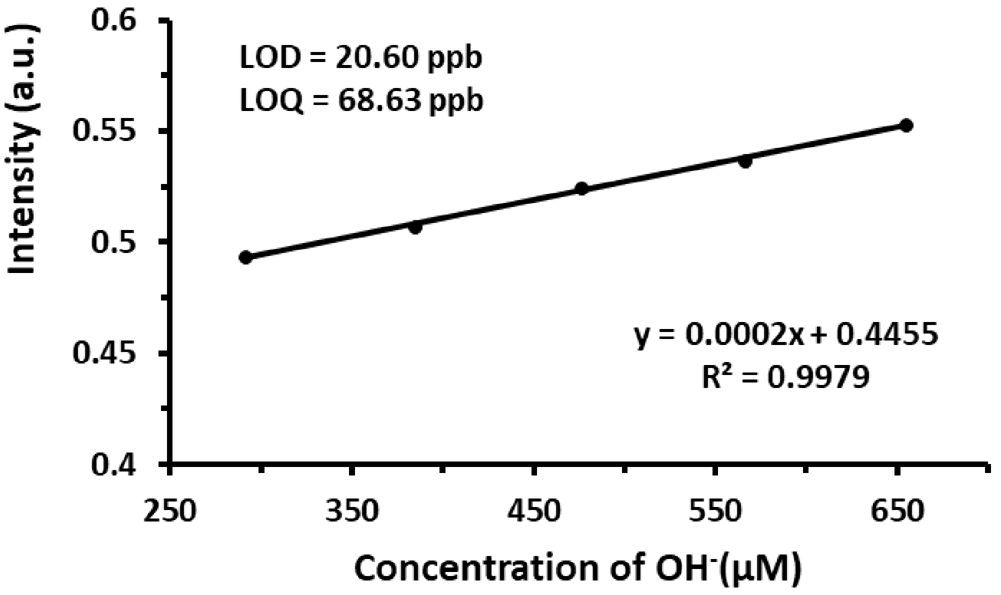


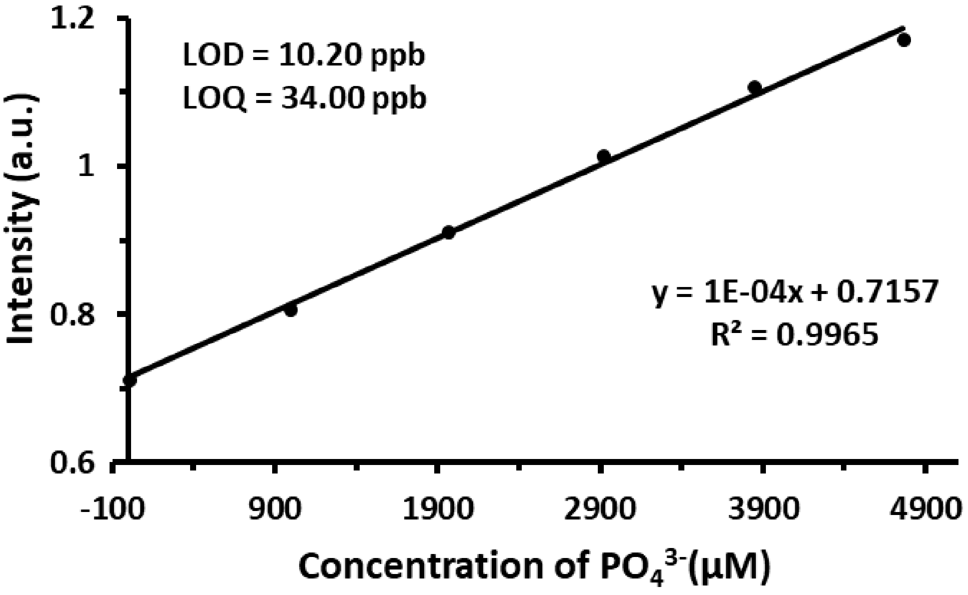


**Figure S10g.** Calibration curve from AnSQ fluorescence upon addition of $OH^-$ and $PO_4^{3-}$ for limit of detection (LOD) and limit of qualitative (LOQ) calculation in acetone

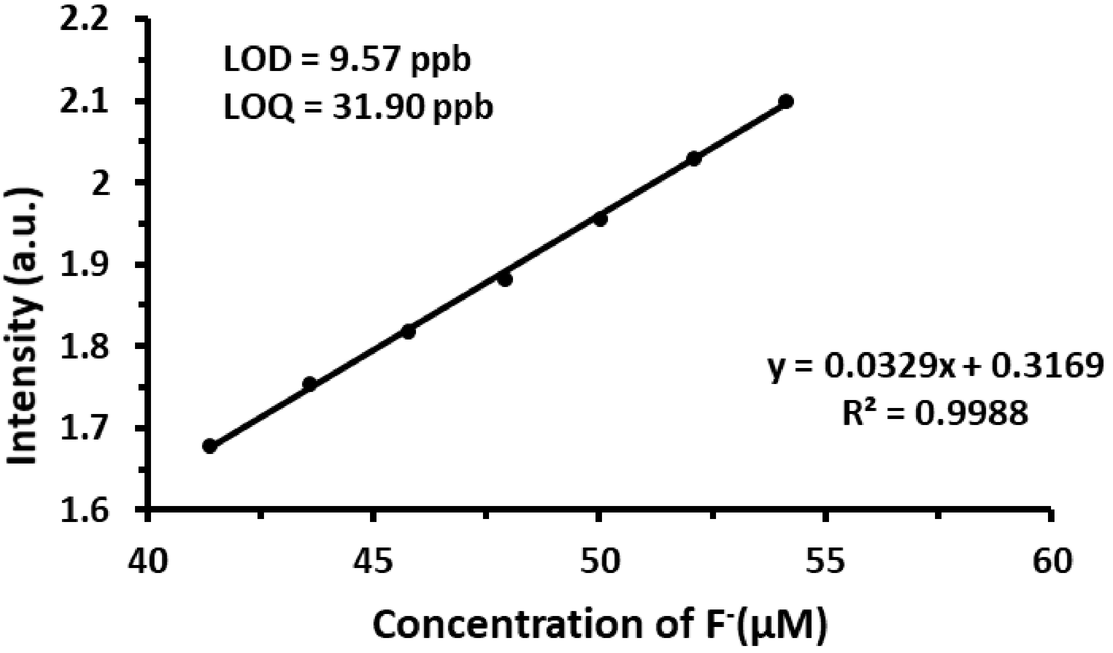


**Figure S10h.** Calibration curve from AnSQ fluorescence upon addition of $F^-$ limit of detection (LOD) and limit of qualitative (LOQ) calculation in toluene

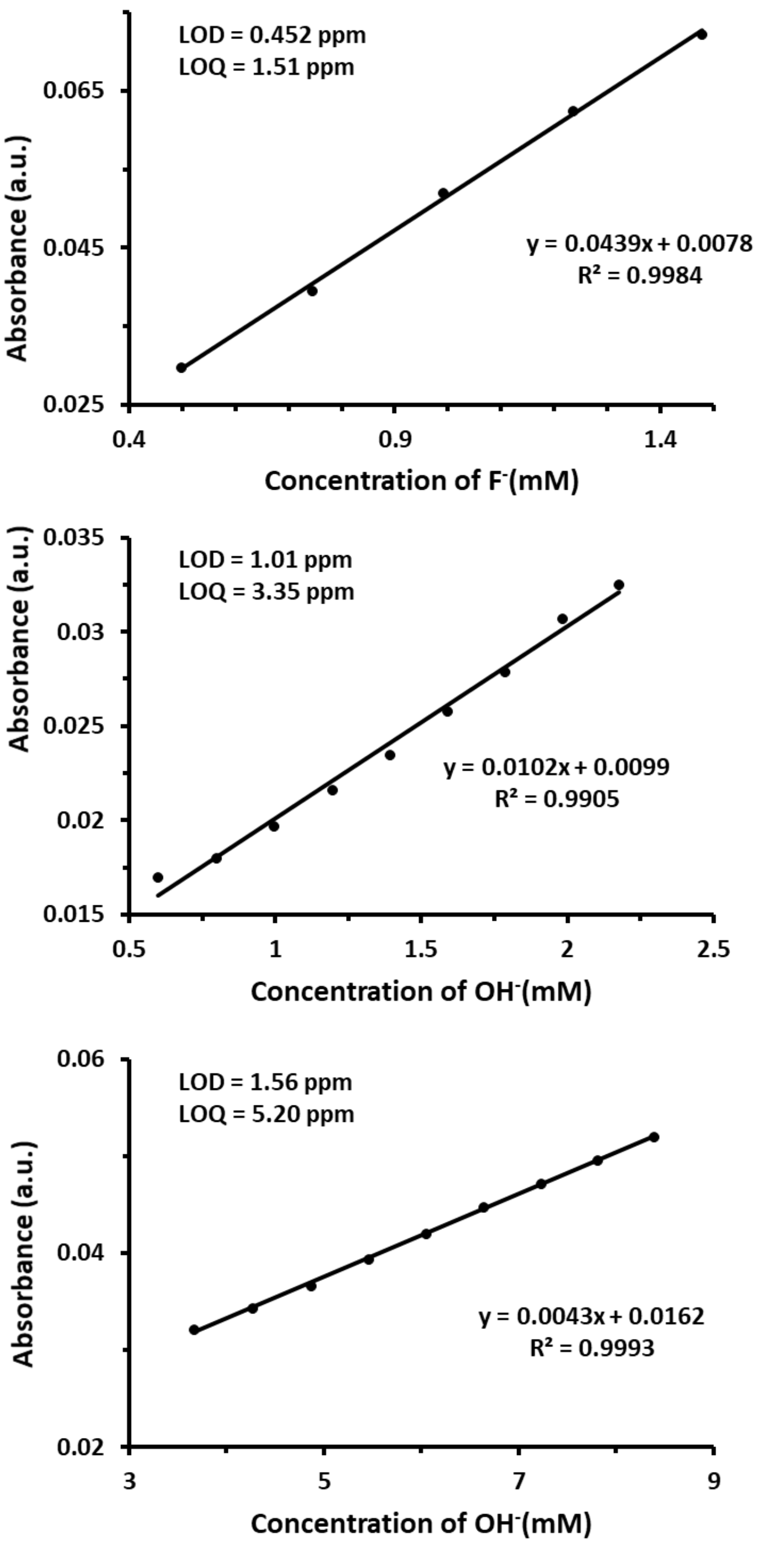


**Figure S10i.** Calibration curve from AnSQ absorbance upon addition of $F^-$, $OH^-$ and $CN^-$ for limit of detection (LOD) and limit of qualitative (LOQ) calculation in THF

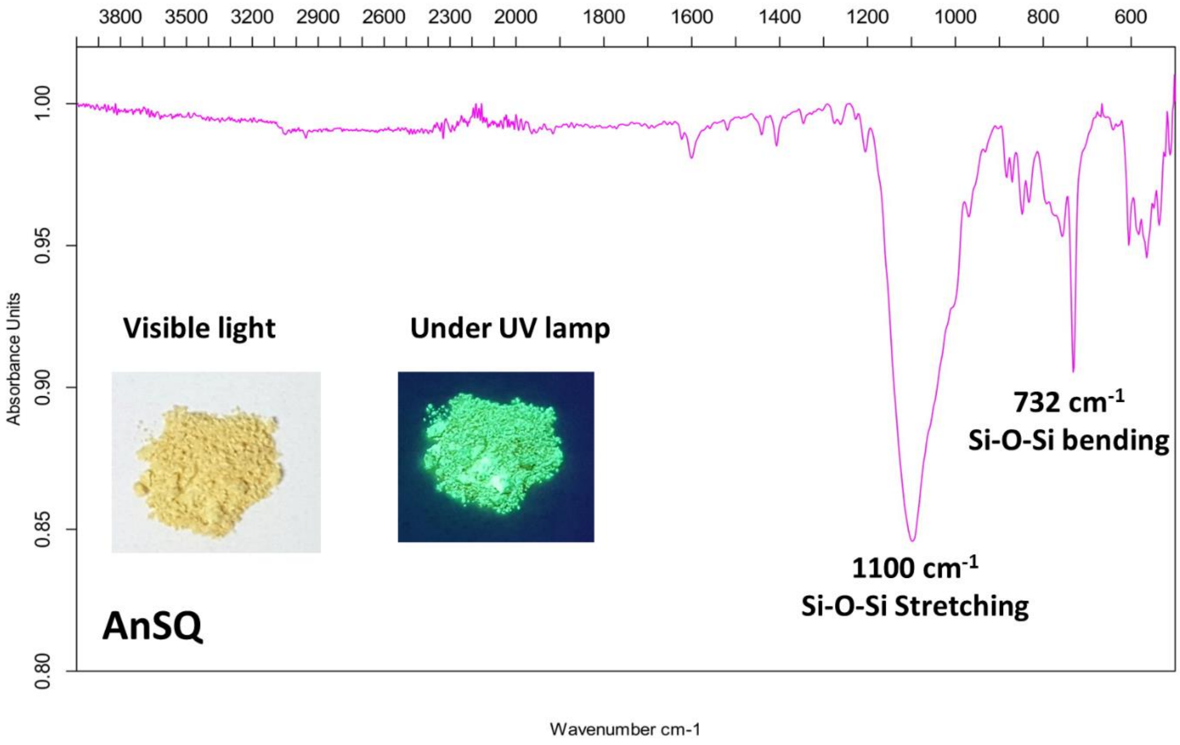


**Figure S11a.** FTIR of solid state AnSQ. The peaks at 1100 cm$^{-1}$ is assigned as Si-O-Si stretching, and the peaks at 732 cm$^{-1}$ is assigned as Si-O-Si bending

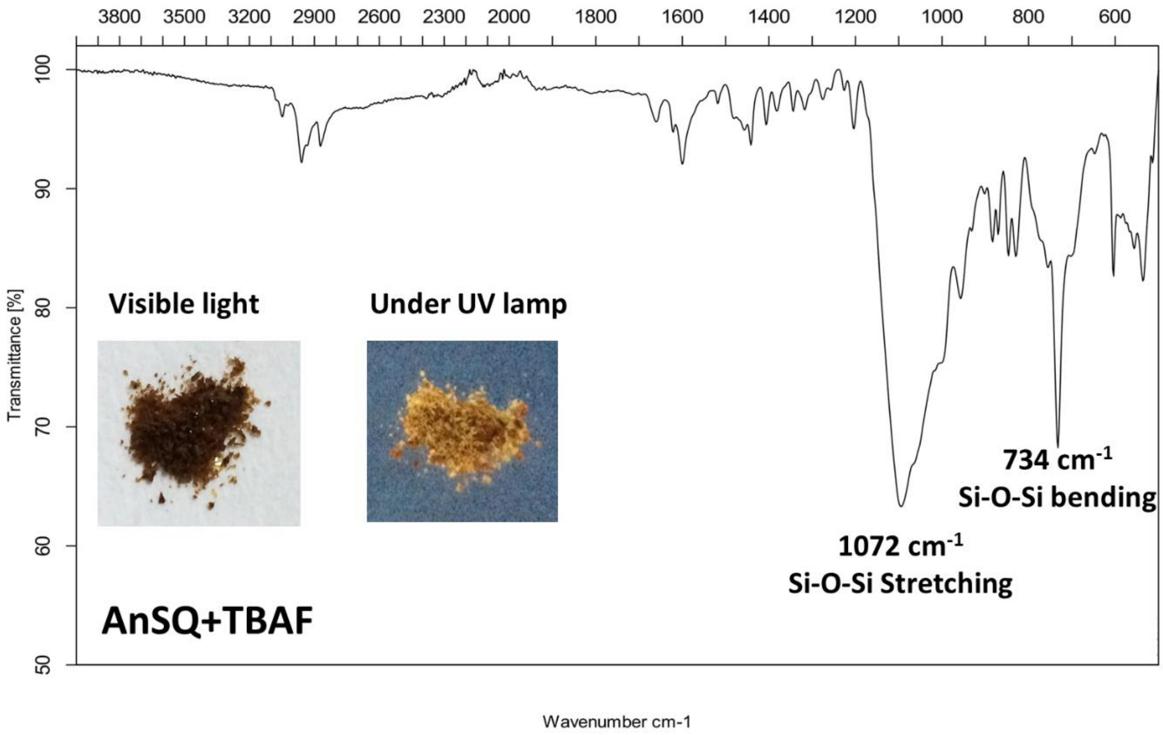


**Figure S11b.** FTIR of solid state AnSQ+TBAF. The peaks at 1072 cm$^{-1}$ is assigned as Si-O-Si stretching, and the peaks at 734 cm$^{-1}$ is assigned as Si-O-Si bending

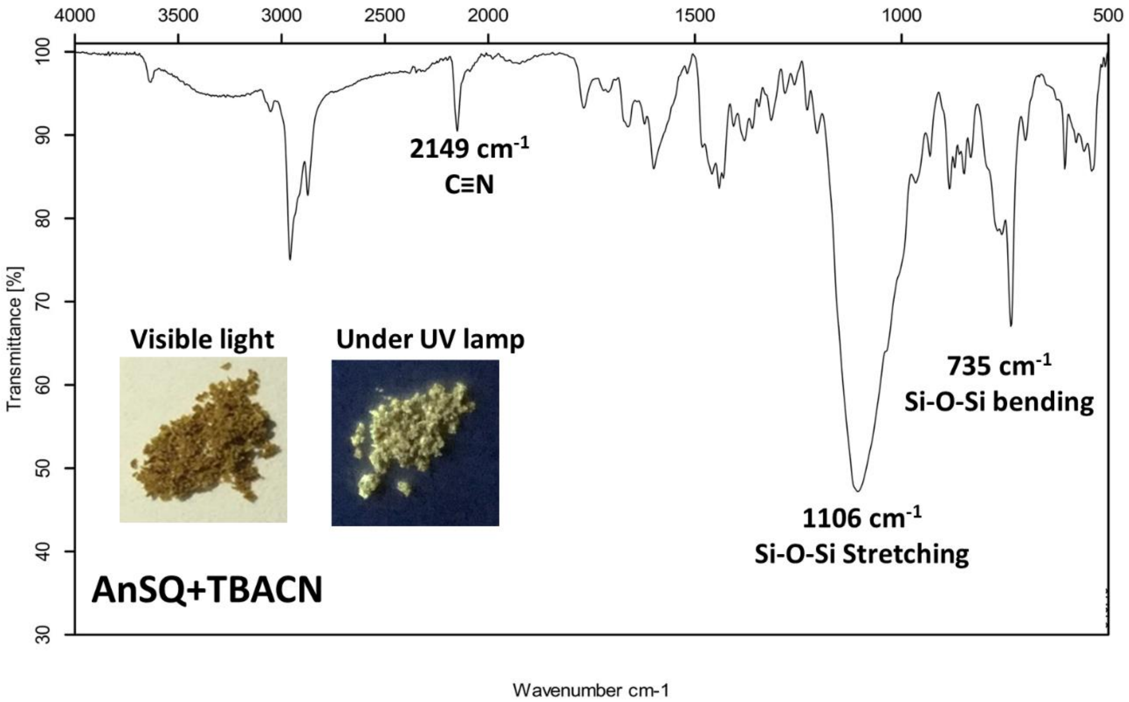


**Figure S11c.** FTIR of solid state AnSQ+TBACN. The peaks at 1106 $cm^{-1}$ is assigned as Si-O-Si stretching, and the peaks at 735 $cm^{-1}$ is assigned as Si-O-Si bending, the peak at 2149 $cm^{-1}$ is assigned as C ≡N stretching

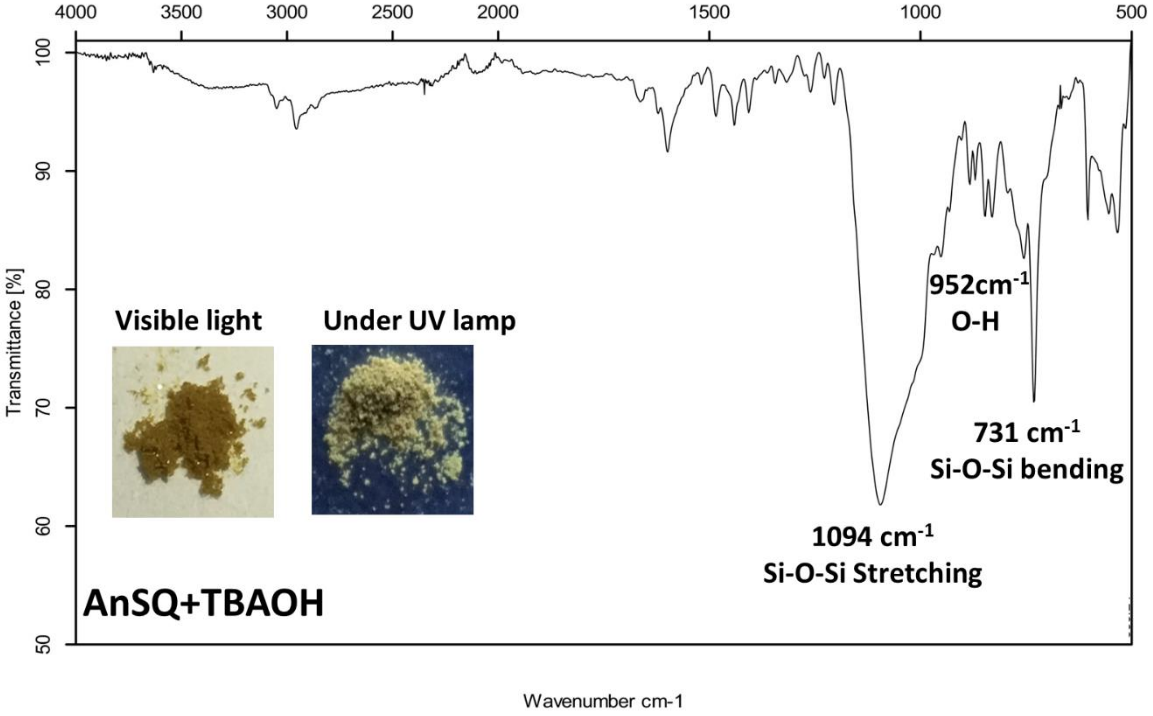


**Figure S11d.** FTIR of solid state AnSQ+TBAOH. The peaks at 1094 $cm^{-1}$ is assigned as Si-O-Si stretching, and the peaks at 731 $cm^{-1}$ is assigned as Si-O-Si bending, the peak at 952 $cm^{-1}$ is assigned as O-H bending

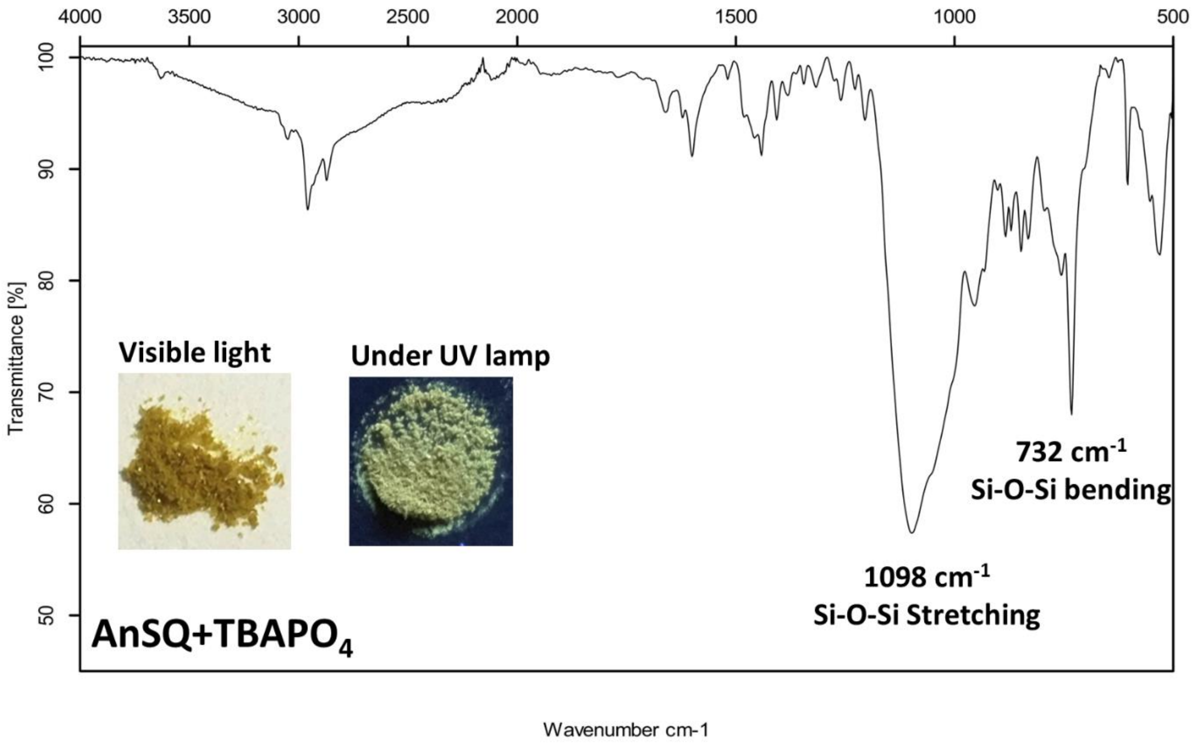


**Figure S11e.** FTIR of solid state AnSQ+TBAPO4. The peaks at 1098 cm$^{-1}$ is assigned as Si-O-Si stretching, and the peaks at 732 cm$^{-1}$ is assigned as Si-O-Si bending, the characteristic peak of phosphate is dominated by Si-O-Si stretching

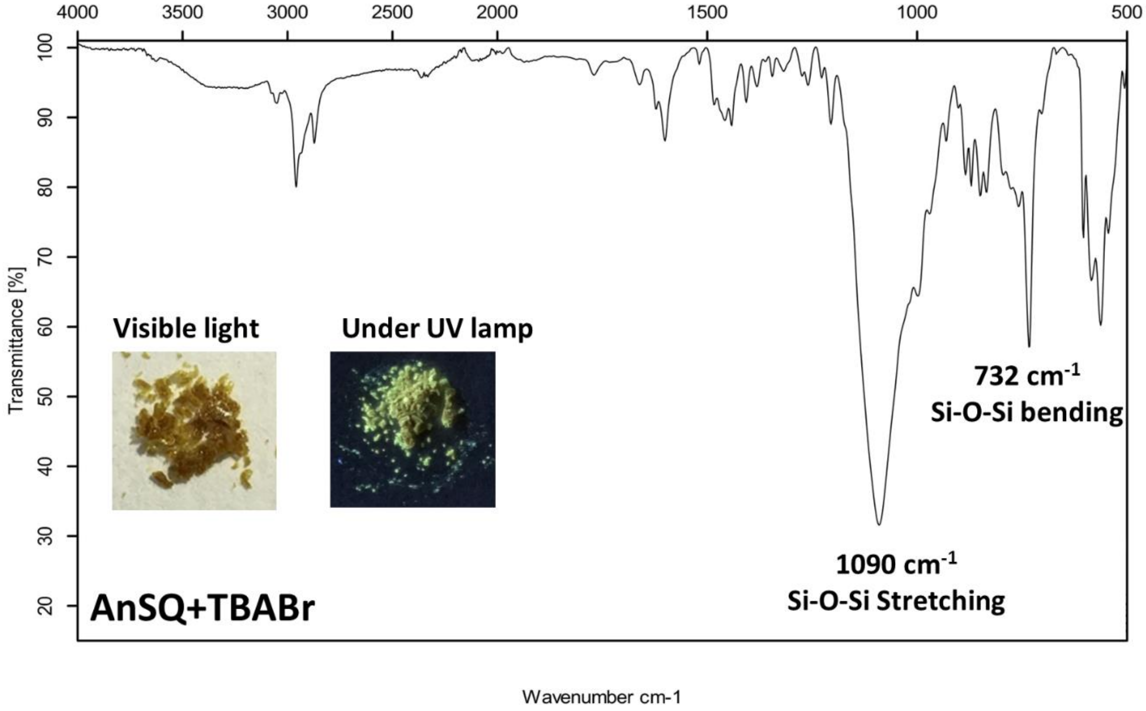


**Figure S11f.** FTIR of solid state AnSQ+TBABr. The peaks at 1090 cm$^{-1}$ is assigned as Si-O-Si stretching, and the peaks at 732 cm$^{-1}$ is assigned as Si-O-Si bending.

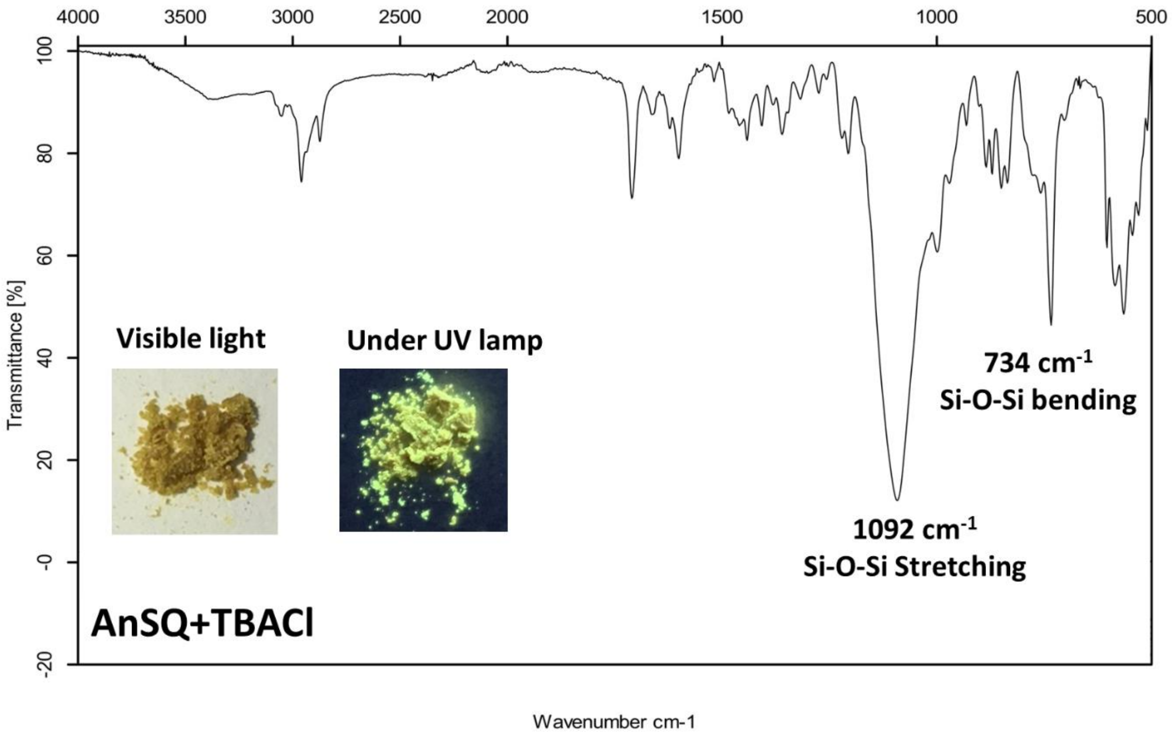


**Figure S11g.** FTIR of solid state AnSQ+TBACl. The peaks at 1092 $cm^{-1}$ is assigned as Si-O-Si stretching, and the peaks at 734 $cm^{-1}$ is assigned as Si-O-Si bending.

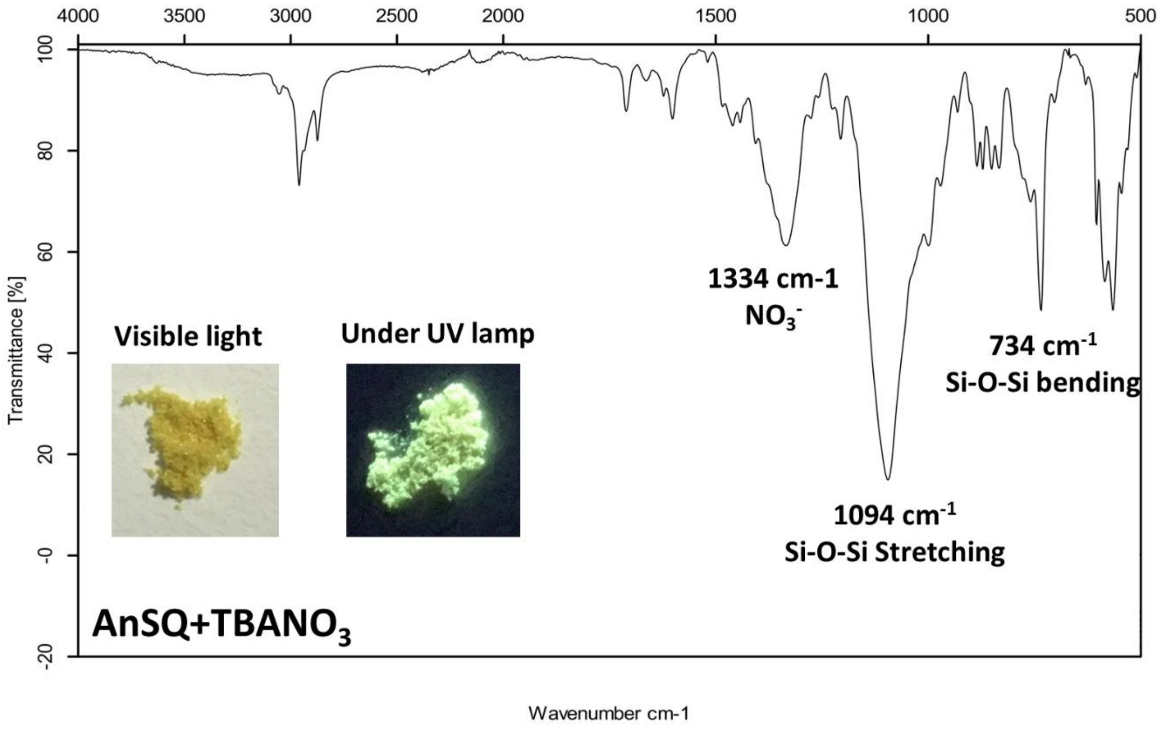


**Figure S11h.** FTIR of solid state $AnSQ+TBANO_3$. The peaks at 1094 $cm^{-1}$ is assigned as Si-O-Si stretching, and the peaks at 734 $cm^{-1}$ is assigned as Si-O-Si bending. The characteristic of nitrate peak is assigned at 1334 $cm^{-1}$
